# Isosarcophytoxide Derivatives with a 2,5-Dihydrofuran Moiety from the Soft Coral *Sarcophyton cinereum*

**DOI:** 10.3390/molecules28020641

**Published:** 2023-01-08

**Authors:** Chih-Hua Chao, Yuan-Jhong Wu, Tzu-Yin Huang, Chi-Jen Tai, Yi-Ju Chen, Chiung-Yao Huang, Chi-Chien Lin, Chang-Feng Dai, Hui-Chi Huang, Jyh-Horng Sheu

**Affiliations:** 1 School of Pharmacy, China Medical University, Taichung 404, Taiwan; 2 Chinese Medicine Research and Development Center, China Medical University Hospital, Taichung 404, Taiwan; 3 Department of Marine Biotechnology and Resources, National Sun Yat-sen University, Kaohsiung 804, Taiwan; 4 Institute of Biological Chemistry, Academia Sinica, Taipei 115, Taiwan; 5 National Museum of Marine Biology and Aquarium, Pingtung 944, Taiwan; 6 Institute of Biomedical Science, National Chung-Hsing University, Taichung 402, Taiwan; 7 Institute of Oceanography, National Taiwan University, Taipei 112, Taiwan; 8 Department of Chinese Pharmaceutical Sciences and Chinese Medicine Resources, China Medical University, Taichung 404, Taiwan; 9 Department of Medical Research, China Medical University Hospital, China Medical University, Taichung 404, Taiwan; 10 Graduate Institute of Natural Products, Kaohsiung Medical University, Kaohsiung 807, Taiwan

**Keywords:** 16-hydroperoxyisosarcophytoxide, 16-methoxyisosarcophytoxide, *Sarcophyton cinereum*, cytotoxicity, DU8, 2,5-dihydrofuran, immunosuppressive

## Abstract

The present chemical investigation on the organic extract of the soft coral *Sarcophyton cinereum* has contributed to the isolation of four new cembranoids: 16*β*- and 16*α*-hydroperoxyisosarcophytoxides (**1** and **2**), 16*β*- and 16*α*-methoxyisosarcophytoxides (**3** and **4**), and a known cembranoid, lobocrasol (**5**). The structures of all isolates were elucidated by detailed spectroscopic analysis. Their structures were characterized by a 2,5-dihydrofuran moiety, of which the relative configuration was determined by DU8-based calculation for long-range coupling constants (^4^*J*_H,H_). The cytotoxicity and immunosuppressive activities of all isolates were evaluated in this study.

## 1. Introduction

**Figure 1:**
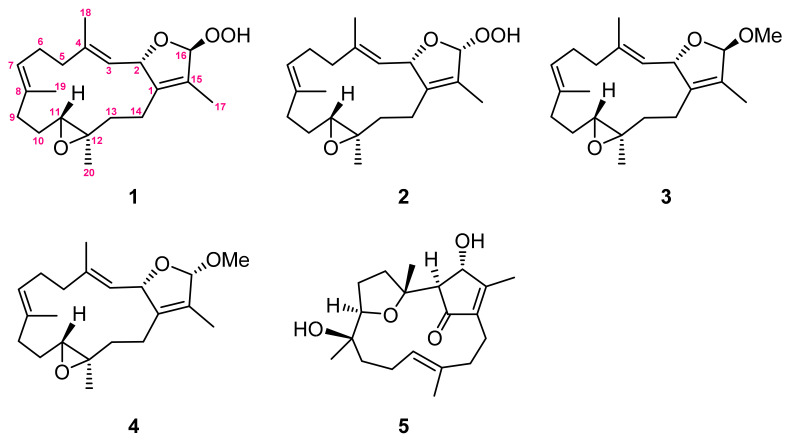
Structures of metabolites **1**–**5**.

Marine soft corals, in particular those of the *Sarcophyton* genus, have been considered to be a valuable reservoir of bioactive natural products, such as sesquiterpenoids, diterpenoids, steroids, and fatty acids [1]. Among them, the cembrane-type diterpenoids and related analogues are the most characteristic and abundant metabolites [2,3,4,5,6,7,8,9,10,11,12,13,14,15,16,17,18,19,20,21]. These structurally diversified metabolites have been reported to possess various biological activities, such as cytotoxic [9,10,11], anti-inflammatory [9,10,11,12,13,14,15,16,17,18], neuroprotective [19], anti-bacterial [19,20,21], and anti-viral activities [19,22]. Also, some of the cembranids have shown the ability to protect the host against predators and stressful environments [7,8]. The chemical investigations of the *Sarcophyton* genus have been widely reported [1], and the high chemical diversity of cembranoids from this genus has been suggested as originating from different genetically distinct clades [4]. Recently, our chemical investigation led to the discovery of a new cembranoid with a novel skeleton, a new sarsolenone [10], and other cembranoids of various types, such as biscembranoids [11]. These results and the notable biological activities further prompted us to explore the unknown natural products from this soft coral. Our present chemical investigation of the *S. cinereum* has led to the isolation of four new cembranoids: 16β- and 16α-hydroperoxyisosarcophytoxides (**1** and **2**) and 16β- and 16α-methoxyisosarcophytoxides (**3** and **4**), as well as a previously reported one, lobocrasol (**5**) [23,24,25] (Figure 1). In this study, the cytotoxicity and immunosuppressive activity of all isolates are reported herein.

## 2. Results and Discussion

The specimen of *S. cinereum* was extracted with EtOAc. The oily residue was repeatedly purified by column chromatography to afford four new compounds **1**–**4** and one known diterpene **5**. The chemical structures of all metabolites were elucidated by analyzing IR, MS, 1D, and 2D NMR spectra, as well as *J*-based DU8 quantum chemical calculations (Supplementary Materials Figures S1–S32).

The HRESIMS of 16*β*-hydroperoxyisosarcophytoxide (**1**), white amorphous powder, showed a sodiated peak at *m*/*z* 357.2039 [M + Na]^+^ (calculated for 357.2037, C_20_H_30_O_4_Na), indicating a molecular formula of C_20_H_30_O_4_ and six degrees of unsaturation. IR spectrum displayed absorption bands at 3462 and 1660 cm^−1^, suggesting the presence of hydroxy and olefinic functional groups. The ^13^C NMR spectra showed 20 signals (Table 1), including four methyl groups, six *sp^3^* methylenes, three *sp^3^* methines, two *sp^2^* methines, one *sp^3^* quaternary carbon, and four *sp^2^* quaternary carbons. The ^1^H and ^13^C NMR spectra revealed the signals characteristic of two tri-substituted double bonds (*δ*_H_ 4.95, d, *J* = 10.3 Hz; *δ*_C_ 126.5, CH; 141.0, C; and *δ*_H_ 4.84, d, *J* = 9.2 Hz; *δ*_C_ 125.9, CH; 133.8, C), one tetra-substituted double bond (*δ*_C_ 125.9, C; 141.9, C), one oxygen-bearing group (*δ*_H_ 5.59, d, *J* = 10.3 Hz; *δ*_C_ 83.2, CH), one acetal (*δ*_H_ 6.10, d, *J* = 3.3 Hz; *δ*_C_ 114.8, CH), and one tri-substituted epoxy group (*δ*_H_ 2.45, dd, *J* = 10.9, 2.7 Hz; *δ*_C_ 61.9, CH; 60.8, C).

**Figure 2:**
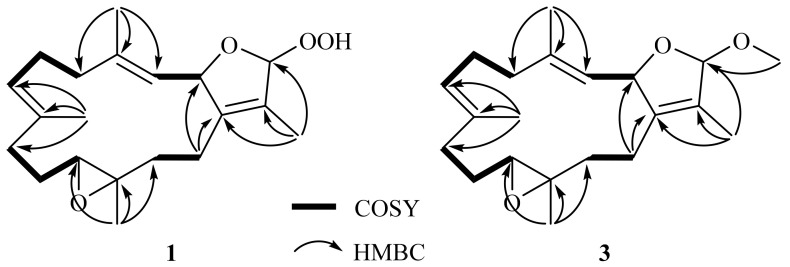
COSY and selective HMBC correlations of **1** and **3**.

The gross structure of **1** was established by 2D NMR spectra, including correlation spectroscopy (COSY) and heteronuclear multiple bond correlation (HMBC) spectroscopic data (Figure 2). Analysis of COSY correlations suggested four partial structures from H-2 to H-3, H_2_-5 to H-7, H_2_-9 to H-11, and H_2_-13 to H_2_-14 (Figure 2). These moieties were connected by HMBC correlations of H_3_-17 to C-1, C-15, and C-16; H_3_-18 to C-3, C-4, and C-5; H_3_-19 to C-7, C-8, and C-9; H_3_-20 to C-11, C-12, and C-13; and H_2_-14 to C-1 (Figure 2). Although the HMBC correlation of H-16 (or H-2) to C-2 (or C-16) was not observed, the downfield shifts of C-2 and C-16 suggested an ether linkage between C-2 and C-16 to generate a 3-methyl-2,5-dihydrofuran moiety (Figure 2) [17]. Furthermore, the hydroperoxy group with the proton resonance appearing at *δ*_H_ 8.36 was assigned to attach at the acetal carbon C-16 due to its downfield chemical shifts.

**Figure 3:**
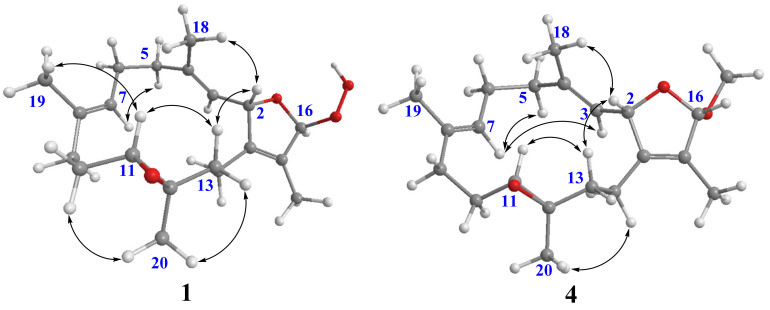
Selective NOE correlations of **1** and **4**.

16*β*-Hydroperoxyisosarcophytoxide (**2**) was also isolated as a white amorphous powder. It was found to have the same molecular formula as that of **1**. A comparison of the NMR data of **2** with those of **1** suggested that they are structurally related and should be epimeric at C-16. The NOE correlations of H-2/H-13*β* and H-11/H-13*β* were observed for both compounds, which were consistent with the related analogues possessing the same 2S*,11R*,12R*-configuration (Figure 3) [14]. The upfield chemical shifts for both C-18 and C-19 (*δ*_C_ 14.90 and *δ*_C_ 14.8, respectively, in compound **1**; *δ*_C_ 14.8 and *δ*_C_ 14.9, respectively, in compound **2**) indicated the *E* geometry of C-3/C-4 and C-7/C-8 double bonds. A comparison of their NMR data coupled with the above analysis supported **2** to be a C-16 epimer of **1**. However, due to the lack of NOE correlations of H-16 with other protons in both compounds, the C-16 configurations of **1** and **2** were unable to be determined.

As compounds **1** and **2** exhibited very similar chemical shifts, the result of DP4+ probability analysis might become occasionally erratic and lead to a wrong assignment [26]. A comparison of their COSY spectra revealed that they showed distinct differences at cross peaks between H-2 and H-16 (Figures S7 and S23), in which the COSY cross peak of H-2/H-16 was observed only for compound **1**. This implied that **1** has a larger long-range proton-proton coupling constant (^4^*J*_2,16_) than **2**, which is also consistent with the experimental data (Table 1). In view of their differences observed in the COSY spectra and coupling patterns, a solution to the stereochemistry assignment was performed using the reported quantum chemical calculations for *J*-value based on the DU8 basis set [27]. The ^4^*J*_2,16_ coupling constants of 16α-H and 16β-H epimers (i.e., *trans*-2,5-dihydrofuran and *cis*-2,5-dihydrofuran analogues, Table 2) were calculated at B3LYP/DU8//B3LYP-D3(BJ)/6-31+G(d,p) level of theory, and calibrated by both empirical scaling parameters and the NBO hybridization coefficients. The resulting data were weighted by Boltzmann distribution using energies calculated at M06-2X/6-31+G(d,p)//B3LYP-D3(BJ)/6-31+G(d,p) level. The result revealed that the structure with *trans*-2,5-dihydrofuran moiety was found to have larger ^4^*J*_2,16_ coupling constants than that of the *cis* analogue (Table 2). Moreover, the calculated result also showed good agreement with the analysis of *cis* and *trans* 2,5-dihydrofuran analogues reported by Barfield et al. [28].

16*β*-Methoxyisosarcophytoxide (**3**), obtained as a white amorphous powder, with a sodiated ion peak at *m*/*z* 355.2245 ${\left[M+\mathrm{Na}\right]}^{+}$ (calculated 355.2244, C_21_H_32_O_3_Na) in the HRESIMS spectrum suggested a molecular formula of C_21_H_32_O_3_. The ^13^C NMR (Table 1) and DEPT spectra showed 21 signals, including five methyl groups, six methylenes, five methines, and five quaternary carbons. In the NMR spectral data (Table 1), the chemical shifts at *δ*_H_ 3.35 and *δ*_C_ 53.8 suggested the presence of a methoxy group. A detailed analysis of the NMR data of **3** suggested its close similarity with those of **1**. Furthermore, the HMBC correlation of OMe/C-16 (*δ*_C_ 111.8) also demonstrated the attachment of the methoxy group to C-16 (Figure 2).

16*α*-Methoxyisosarcophytoxide (**4**), a white amorphous powder, exhibited the same molecular formula as that of **3**, based on the analysis of its HRESIMS spectrum (found: *m*/*z* 355.2241, calculated: *m*/*z* 355.2244, [M + Na]^+^, C_21_H_32_O_3_Na). The detailed analysis of the 1D and 2D NMR spectra revealed the same planar structure for both **3** and **4**.

The H-2 of compound **3**, similar to the cases of compounds **1** and **2**, also showed a COSY cross peak with H-16 but this was not found in compound **4** (Table 2), implying that **3** may have the same C-16 configuration as **1**, while **4** shared the same C-16 configuration with **2**. This was further confirmed by computational calculation for ^4^*J*_2,16_ coupling constants of both *trans* and *cis* isomers (Table 2), which showed a consistent result with the experimental and literature data [28].

In 1990, Kusumi et al. isolated isosarcophine from the Okinawa soft coral, *Sinularia mayi,* and demonstrated its cytotoxicity against the human colorectal carcinoma (HCT-116) cell line [24]. In 1992, Wu et al. also isolated the same metabolite from Formosna soft coral, *Sarcophyton trocheliophorum,* and reported its cytotoxicity toward cancer cells [29]. A related analogue, isosarcophytoxide, was also reported to exhibit significant cytotoxic and moderate anti-inflammatory properties [30]. Up to date, more related derivatives have been discovered from different soft corals, and diversified biological activities of some cembranoids remain unveiled [3]. In the present study, compounds **1**–**5** were also assayed for their cytotoxicity toward human small cell lung cancer (H1688) cells, and the result showed that **3** possessed a moderate cytotoxicity (IC_50_ = 27.5 ± 6.4 μM) and **4** exhibited weak activity (IC_50_ = 85.8 μM) toward the H1688 cell line, while the other compounds were nontoxic with IC_50_ values over 100 μM. In addition, the isolates were evaluated for their immunosuppressive effect by measuring TNF-α expression in LPS-stimulated murine dendritic cells (DCs). However, none of the isolates could reduce the TNF-α expression (IC_50_ > 100 μM) in LPS-stimulated DCs.

## 3. Materials and Methods

### 3.1. General Experimental Procedures

The NMR experiments with reference to residue signals of C_6_D_6_ (*δ*_H_ 7.16; *δ*_C_ 128.4) were carried out on a Varian Unity INOVA 500 FT-NMR (Varian Inc., Palo Alto, CA, USA). Specific optical rotations were measured in MeOH on the Jasco P-1020 polarimeter (JASCO Corporation, Tokyo, Japan). The IR spectra were recorded on an FT/IR-4100 infrared spectrophotometer (JASCO Corporation, Tokyo, Japan). Measurements of circular dichroism spectra were performed on Jasco J-715 CD spectrometer (JASCO Corporation, Tokyo, Japan). HRESIMS were measured on the Impact HD Q-TOF (Bruker, Bremen, Germany) mass spectrometer. The ESI mode (spray potential 4.0 kV) was used. Data analysis was controlled by Bruker DataAnalysis. Full scan spectra were acquired in the ion peak centroid or profile modes over the mass/charge range of 50–1000. The mass spectra were obtained by direct infusion method. Thin-layer chromatography (TLC) analysis was performed on pre-coated silica gel plates (Silica gel 60 F254, 100 μm, Merck, Darmstadt, Germany) or C18 gel plates (Silica gel 60 RP-18 F_254_s, 100 μm, Merck, Darmstadt, Germany). The open column chromatography was performed on a glass column using adsorbents of silica gel (40–63 μm, Merck, Billerica, USA) or reversed-phase silica gel (RP-18, 40–63 μm, Merck, Darmstadt, Germany). The Hitachi L-2455 HPLC apparatus (Hitachi, Tokyo, Japan) with a Supelco C18 column (250 × 21.2 mm, 5 μm, Supelco, Bellefonte, PA, USA) were used for HPLC purification.

### 3.2. Animal Material

Soft coral *S. cinereum*, collected at a depth of 5–10 m by hand using scuba off the coast of the Xiao Liuqiu islands of Taiwan in 2012, was stored in a freezer until extraction. The species was identified based on its colony shape, the distribution of autozooids and siphonozooids on capitulum, and the morphology of sclerites in capitulum and stalk. These morphological features fit well with those described in literature [31]. A sample was deposited at the Department of Marine Biotechnology and Resources, National Sun Yat-sen University.

### 3.3. Extraction and Separation

The freeze-dried soft coral *S. cinereum* (wet weight: 281.5 g; dry weight: 107.4 g) was minced and subjected for extraction using EtOAc. An oily residue (14.6 g) obtained by vacuum evaporation of the EtOAc extract was chromatographed by a silica gel column with the gradient elution of EtOAc in *n*-hexane (0–100%), acetone in ethyl acetate (0–100%), methanol in acetone (0–100%) to afford 22 fractions. Fraction 7 (1.0 g), eluted by *n*-hexane-EtOAc (8:1), was further purified over silica gel (elution: *n*-hexane-acetone = 6.5:1) to obtain five subfractions (7A–7E). Subfraction 7D was purified by reversed-phase HPLC with the elution of methanol-H_2_O (3:1) to afford **1** (8.8 mg), **2** (7.0 mg), **3** (2.0 mg), and **4** (3.3 mg). Subfraction 7E was purified by reversed-phase HPLC with the elution of methanol-H_2_O (1.3:1) to afford **5** (1.0 mg).

16*α*-Hydroperoxyisosarcophytoxide (**1**): white amorphous powder, ${\left[\alpha \right]}_{D}^{26}$ + 89.8 (*c* 0.4, MeOH), IR (KBr) *v*_max_ 3463, 2922, 1595, 1444, 1380, 1012, 958 cm^−1^; for ^13^C and ^1^H data see Table 1; electrospray ionization mass spectrometry (ESIMS) *m*/*z* 357; HRESIMS *m*/*z* 357.2039 [M + Na]^+^ (calculated for C_20_H_30_O_4_Na: 357.2037).

16*β*-Hydroperoxyisosarcophytoxide (**2**): white amorphous powder, ${\left[\alpha \right]}_{D}^{26}$ + 23.2 (*c* 0.33, MeOH), IR (KBr) *v*_max_ 3391, 2921, 2927, 1445, 1384, 1226, 1000 cm^−1^; for ^13^C and ^1^H data see Table 1; ESIMS *m*/*z* 357; HRESIMS *m*/*z* 357.2036 [M + Na]^+^ (calculated for C_20_H_30_O_4_Na: 357.2037).

16*α*-Methoxyisosarcophytoxide (**3**): white amorphous powder, ${\left[\alpha \right]}_{D}^{26}$ + 121.7 (*c* 0.3, MeOH), IR (KBr) *v*_max_ 2923, 1661, 1593, 1445, 1378, 1090, 1012 cm^−1^; for ^13^C and ^1^H data (see Table 1); ESIMS *m*/*z* 355; HRESIMS 355.2245 *m*/*z* [M + Na]^+^ (calculated for C_21_H_32_O_3_Na: 355.2244).

16*β*-Methoxyisosarcophytoxide (**4**): white amorphous powder, ${\left[\alpha \right]}_{D}^{26}$ + 21.5 (*c* 0.26, MeOH), IR (KBr) *v*_max_ 2923, 1661, 1593, 1443, 1377, 1090, 1011 cm^−1^; for ^13^C and ^1^H data (see Table 1); ESIMS *m*/*z* 355; HRESIMS *m*/*z* 355.2241 [M + Na]^+^ (calculated for C_21_H_32_O_3_Na: 355.2244).

### 3.4. Proton-Proton Coupling Constant Calculation

The conformers within the 10 Kcal/mol energy window were computed at the MMFF94 force field with the aid of a GMMX package implemented in Gaussian 16 [32]. After the removal of duplicates, the conformers of candidate structures were subjected to geometry optimizations at the B3LYP/6-31G(d) level in gas phase with Natural Bond Orbital (NBO) analysis. Further optimization and frequency calculations were performed at B3LYP-D3(BJ)/6-31+G(d,p) level using the polarizable continuum model (PCM) in benzene. The proton-proton coupling constants were calculated at B3LYP/DU8 level and the resulting data were scaled using both empirical scaling parameters for ^4^*J*_(1,3)_-type and the NBO hybridization coefficients [27], and the data were weighted according to Boltzmann populations using SCF energies refined at M06-2X/6-31+G(d,p)//B3LYP-D3(BJ)/6-31+G(d,p) level in benzene with solvation model based on density (SMD).

### 3.5. Cytotoxicity Assay

The cytotoxicities of compounds were analyzed by using the human small cell lung cancer H1688 cell line and the 3-(4,5-dimethylthiazol-2-yl)-2,5-diphenyl tetrazolium bromide (MTT, Sigma-Aldrich, St. Louis, MO, USA) assay [33,34] at 37 °C for 24 h. (3R*,4S*,5*E*,7*Z*)-7-dichlorothyl-3,4,8-trichloro-3-methylocta-1,5,7-triene was used as a positive control with IC_50’s_ of 4.2 ± 2.1 μM [35].

### 3.6. Immunosupressive Activity

#### 3.6.1. Cell Viability of Dendritic Cells (DCs)

The DCs (1 × 10^6^) were treated with tested compounds (100 μg/mL) and cultured at 37 °C for 24 h. Cytotoxicity was examined using a Cell Counting Kit-8 (CCK-8; Dojindo Molecular Technologies, Inc., Kumamoto, Japan) according to the manufacturer’s protocol. The absorbance was recorded on a microplate reader (Tecan Group Ltd., Männedorf, Switzerland) at a wavelength of 450 nm [36,37]. Quercetin (50 μM) was used as a positive control, which showed cytotoxicity against DCs with IC_50’s_ of 78.8 ± 7.3 μM.

#### 3.6.2. Measurement of TNF-α Expression by LPS-Induced DCs

The TNF-α expressions of LPS-induced DCs were measured by enzyme-link immunosorbent assay (ELISA), in the same way as the previously reported method [38,39]. The DCs were treated with 100 ng/mL lipopolysaccharide (LPS) and the isolated compounds for 24 h. The optical density of the production of TNF-α was measured at 450 nm by the ELISA reader. Quercetin (50 μM) was used as a positive control, which inhibited TNF-α expressions on LPS-induced DCs with IC_50’s_ of 23.1 ± 5.2 μM.

## 4. Conclusions

Our present chemical investigation on the soft coral *S. cinereum* resulted in the isolation of four new isosarcophytoxide derivatives (**1**–**4**) and one previous metabolite, lobocrasol (**5**), in which compounds **3** and **4** were found to show moderate and weak cytotoxicity, respectively, against H1688 cells. The results of this investigation, along with our previous studies [6,9], further evidenced that the soft corals of *Sarcophyton* genus are important sources of structurally diversified cembranoid derivatives. Structurally, the lack of NOE correlations for C-16 substituted 2,5-dihydrofuran analogues **1**–**4** could hinder the elucidation of the relative configuration. Our present work successfully resolved this problem using quantum chemical calculations, which might provide a useful strategy for the determination of relative configurations of other similar cases in the future.

## Figures and Tables

**Table 1:** ^1^H (400 MHz) and ^13^C NMR (100 MHz) spectroscopic data of **1**–**4** in C_6_D_6_.

Position	1		2		3		4	
	*δ*_H_ (Hz)	*δ*_C_	*δ*_H_ (Hz)	*δ*_C_	*δ*_H_ (Hz)	*δ*_C_	*δ*_H_ (Hz)	*δ*_C_
1		141.9 (C)		140.9 (C)		139.3 (C)		138.9 (C)
2	5.59 d (10.3)	83.2 (CH)	5.30 d (10.4)	82.9 (CH)	5.58, d (10.0)	82.9 (CH)	5.37, d (11.2)	82.5 (CH)
3	4.95 d (10.3)	126.5 (CH)	5.11 d (10.4)	127.1 (CH)	5.01, d (10.0)	127.4 (CH)	5.21, d (10.4)	128.2 (CH)
4		141.0 (C)		140.5 (C)		140.0 (C)		139.5 (C)
5	2.07 m; 2.00 m	39.3 (CH_2_)	2.07 m; 2.00 m	39.2 (CH_2_)	2.09 m; 1.95 m	39.3 (CH_2_)	2.01 m; 1.96 m	39.2 (CH_2_)
6	2.14 m; 1.82 m	24.8 (CH_2_)	2.15 m; 1.85 m	24.8 (CH_2_)	2.13 m; 1.84 m	24.8 (CH_2_)	2.12 m; 1.83 m	24.8 (CH_2_)
7	4.84 d (9.2)	125.9 (CH)	4.84 d (9.2)	126.0 (CH)	4.86 d (8.7)	126.0 (CH)	4.85 d (9.4)	126.2 (CH)
8		133.8 (C)		133.7 (C)		133.7 (C)		133.5 (C)
9	2.03 m; 1.83 m	37.3 (CH_2_)	2.05 m; 1.85 m	37.3 (CH_2_)	2.02 m; 1.85 m	37.4 (CH_2_)	2.01 m; 1.85 m	37.3 (CH_2_)
10	1.95 m; 1.11 m	24.6 (CH_2_)	1.96 m; 1.12 m	24.6 (CH_2_)	1.99 m; 1.11 m	24.6 (CH_2_)	2.00 m; 1.13 m	24.7 (CH_2_)
11	2.45 dd (10.9, 2.7)	61.9 (CH)	2.45 dd (10.7, 2.9)	61.9 (CH)	2.45, dd (10.8, 2.8)	61.9 (CH)	2.48, dd (10.7, 2.8)	61.9 (CH)
12		60.8 (C)		60.9 (C)		60.9 (C)		61.0 (C)
13	1.83 m; 1.01 m	38.2 (CH_2_)	1.79 m; 0.99 m	38.3 (CH_2_)	1.84 m; 1.04 m	38.3 (CH_2_)	1.83 m; 1.06 m	38.5 (CH_2_)
14	2.06 m; 1.66 m	23.3 (CH_2_)	2.09 m; 1.64 m	23.4 (CH_2_)	2.12 m; 1.71 m	23.3 (CH_2_)	2.14 m; 1.71 m	23.4 (CH_2_)
15		125.9 (C)		126.1 (C)		128.6 (C)		128.8 (C)
16	6.10 d (3.3)	114.8 (CH)	5.97 brs	114.8 (CH)	5.67 d (3.4)	111.8 (CH)	5.58 brs	111.9 (CH)
17	1.55 s	10.3 (CH_3_)	1.55 s	10.3 (CH_3_)	1.62 s	10.3 (CH_3_)	1.62 s	10.3 (CH_3_)
18	1.38 s	14.90 (CH_3_)	1.34 s	14.8 (CH_3_)	1.30 s	14.8 (CH_3_)	1.34 s	14.7 (CH_3_)
19	1.37 s	14.87 (CH_3_)	1.38 s	14.9 (CH_3_)	1.36 s	14.9 (CH_3_)	1.37 s	14.8 (CH_3_)
20	1.09 s	16.2 (CH_3_)	1.10 s	16.3 (CH_3_)	1.12 s	16.3 (CH_3_)	1.13 s	16.4 (CH_3_)
OOH	8.36 brs		8.00 brs					
OMe					3.35 brs	53.8 (CH_3_)	3.36 brs	53.9 (CH_3_)

**Table 2:** The calculated and experimental ^4^*J*_H,H_ coupling constants, as well as experimental COSY correlations between H-2 and H-16 of compounds **1**–**4**.

Furan Fragment	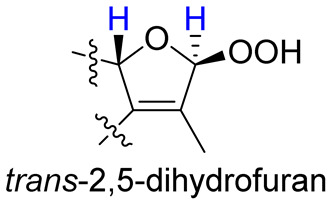	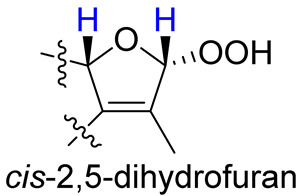	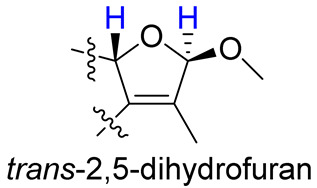	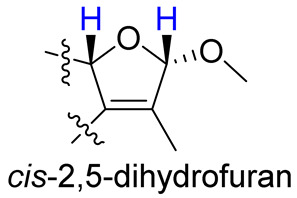
cpd no.	1	2	3	4
Exptl. COSY signal	yes	no	yes	no
Exptl. *J*_2,16_ (Hz)	3.3	– ^a^	3.4	– ^a^
Calc. *J*_2,16_ (Hz) ^b^	4.1	1.5	4.3	1.4

^a^ A sharp singlet was observed; ^b^ *J* values were calculated using DU8 method.

## Data Availability

Data of the present study are available in the article and Supplementary Materials. Samples of the compounds are not available from the authors.

## Supplementary Materials

The supporting information can be downloaded at: https://www.mdpi.com/article/10.3390/molecules28020641/s1, Figures S1–S32: HRESIMS and NMR spectra of compounds **1**–**4**, Tables S1–S4: Cartesian coordinates and Boltzmann populations of compounds **1**–**4**.
