# Supplementary material for: Isosarcophytoxide Derivatives with a 2,5-Dihydrofuran Moiety from the Soft Coral Sarcophyton cinereum
Source: Molecules. 2023 Jan 8;28(2):641. doi: 10.3390/molecules28020641 (PMC9862023; doi:10.3390/molecules28020641)
Supplement: Supplementary file 1 [file molecules-28-00641-s001.zip › molecules-2079288-supplementary.pdf]

Isosarcophytoxide Derivatives with 2,5-Dihydrofuran Moiety from the Soft Coral *Sarcophyton cinereum*

Chih-Hua Chao [1,2,†], Yuan-Jhong Wu [3,†], Tzu-Yin Huang [4,†], Chi-Jen Tai [3,5], Yi-Ju Chen [3], Chiung-Yao Huang [3], Chi-Chien Lin [6], Chang-Feng Dai [7], Hui-Chi Huang [8] and Jyh-Horng Sheu [3,9,10,*]

1 School of Pharmacy, China Medical University, Taichung 404, Taiwan
2 Chinese Medicine Research and Development Center, China Medical University Hospital, Taichung 404, Taiwan
3 Department of Marine Biotechnology and Resources, National Sun Yat-sen University, Kaohsiung 804, Taiwan
4 Institute of Biological Chemistry, Academia Sinica, Taipei, Taiwan
5 National Museum of Marine Biology and Aquarium, Pingtung 944, Taiwan
6 Institute of Biomedical Science, National Chung-Hsing University, Taichung 402, Taiwan
7 Institute of Oceanography, National Taiwan University, Taipei 112, Taiwan
8 Department of Chinese Pharmaceutical Sciences and Chinese Medicine Resources, China Medical University, Taichung 404, Taiwan
9 Department of Medical Research, China Medical University Hospital, China Medical University, Taichung 404, Taiwan
10 Graduate Institute of Natural Products, Kaohsiung Medical University, Kaohsiung 807, Taiwan
* Correspondence: sheu@mail.nsysu.edu.tw; Tel.: +886-7-525-2000 (ext. 5030); Fax: +886-7-525-5020.
† These authors contributed equally to this work.

Figure S1. (+)-HRESIMS spectrum of **1**.
Figure S2. ^{1}H NMR spectrum of **1**.
Figure S3. ^{13}C NMR spectrum of **1**.
Figure S4. DEPT spectrum of **1**.
Figure S5. HSQC spectrum of **1**.
Figure S6. HMBC spectrum of **1**.
Figure S7. COSY spectrum of **1**.
Figure S8. NOESY spectrum of **1**.
Figure S9. (+)-HRESIMS spectrum of **2**.
Figure S10. ^{1}H NMR spectrum of **2**.
Figure S11. ^{13}C NMR spectrum of **2**.
Figure S12. DEPT spectrum of **2**.
Figure S13. HSQC spectrum of **2**.
Figure S14. HMBC spectrum of **2**.
Figure S15. COSY spectrum of **2**.
Figure S16. NOESY spectrum of **2**.
Figure S17. (+)-HRESIMS spectrum of **3**.
Figure S18. ^{1}H NMR spectrum of **3**.
Figure S19. ^{13}C NMR spectrum of **3**.
Figure S20. DEPT spectrum of **3**.
Figure S21. HSQC spectrum of **3**.
Figure S22. HMBC spectrum of **3**.
Figure S23. COSY spectrum of **3**.
Figure S24. NOESY spectrum of **3**.
Figure S25. (+)-HRESIMS spectrum of **4**.
Figure S26. ^{1}H NMR spectrum of **4**.
Figure S27. ^{13}C NMR spectrum of **4**.
Figure S28. DEPT spectrum of **4**.
Figure S29. HSQC spectrum of **4**.
Figure S30. HMBC spectrum of **4**.
Figure S31. COSY spectrum of **4**.
Figure S32. NOESY spectrum of **4**.
Table S1. Conformers and Boltzmann populations of compound **1**.
Table S2. Conformers and Boltzmann populations of compound **2**.
Table S3. Conformers and Boltzmann populations of compound **3**.
Table S4. Conformers and Boltzmann populations of compound **4**

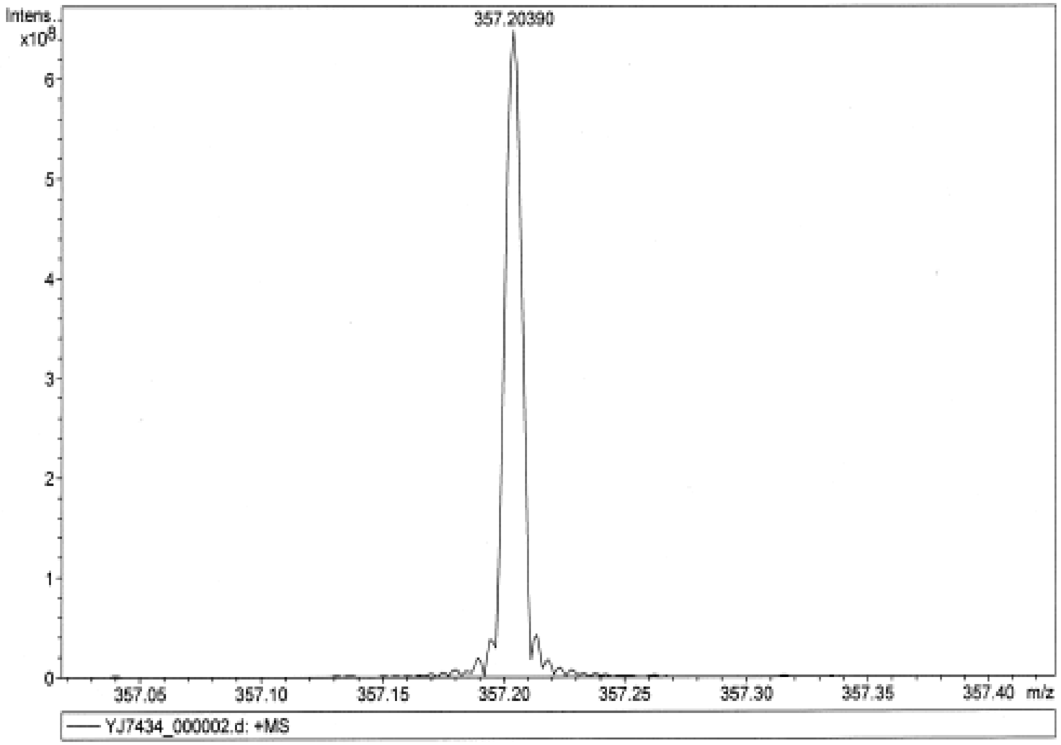

Figure S1. (+)-HRESIMS spectrum of **1**.

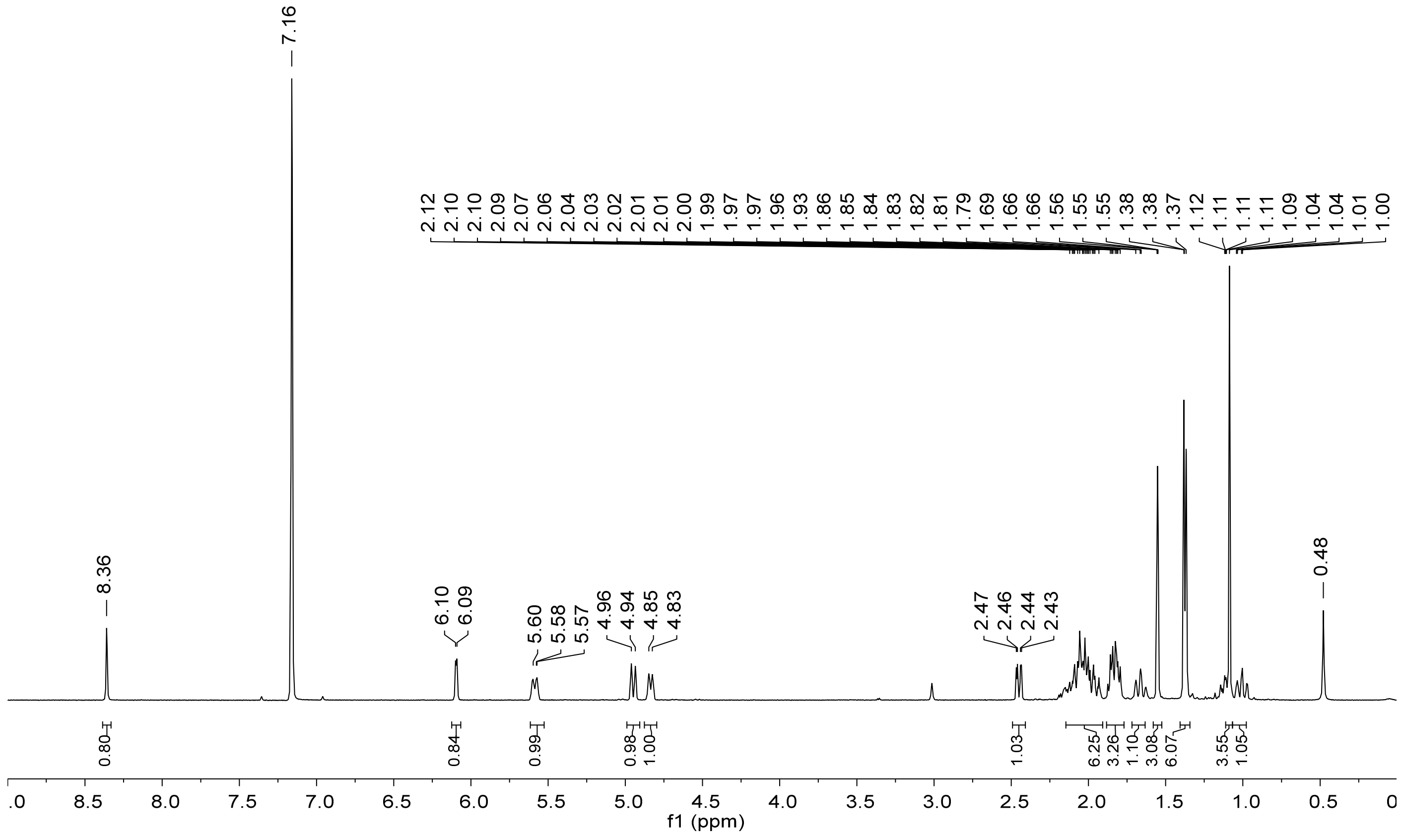

Figure S2. ^{1}H NMR spectrum of **1**.

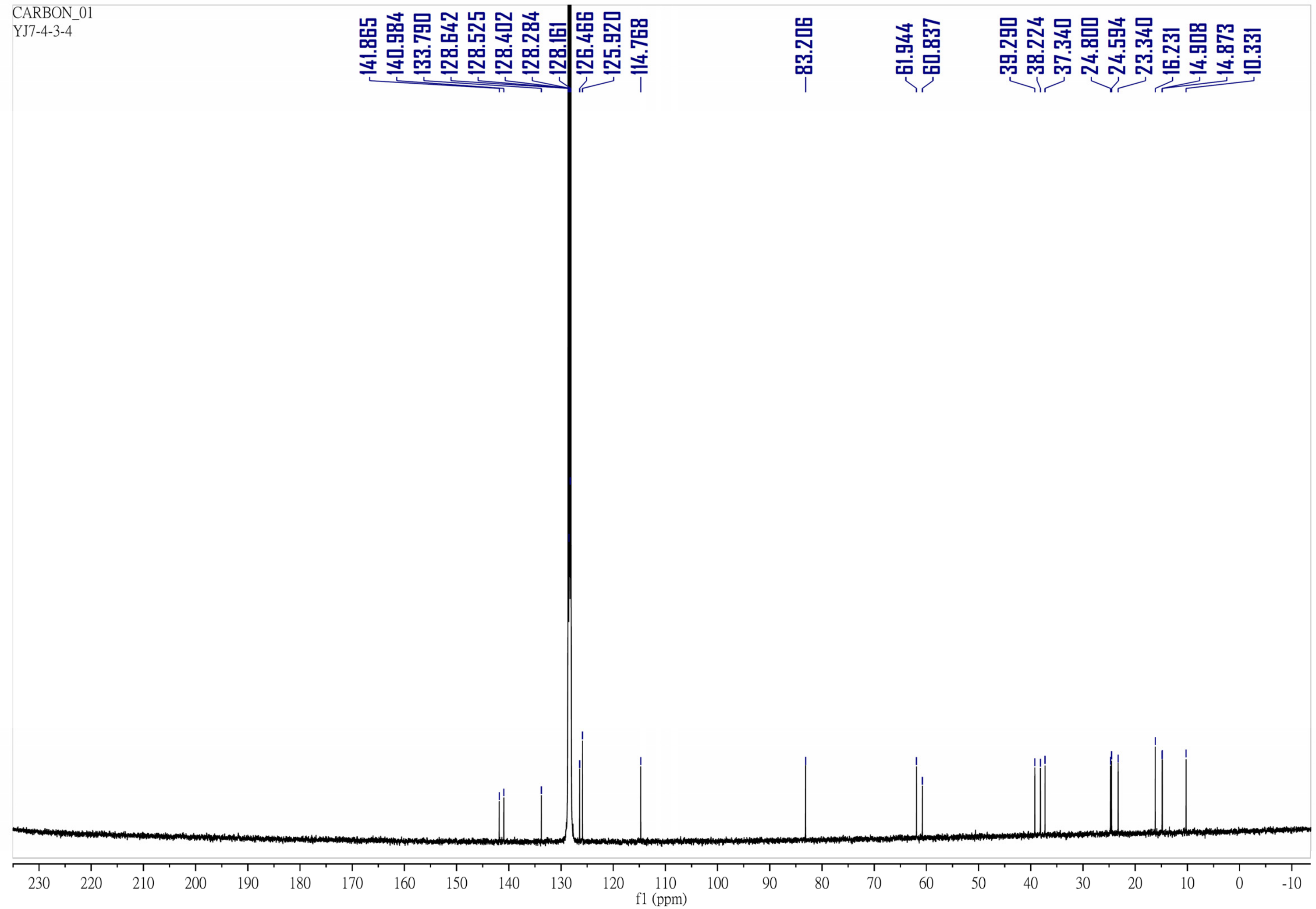

Figure S3. ^{13}C NMR spectrum of **1**.

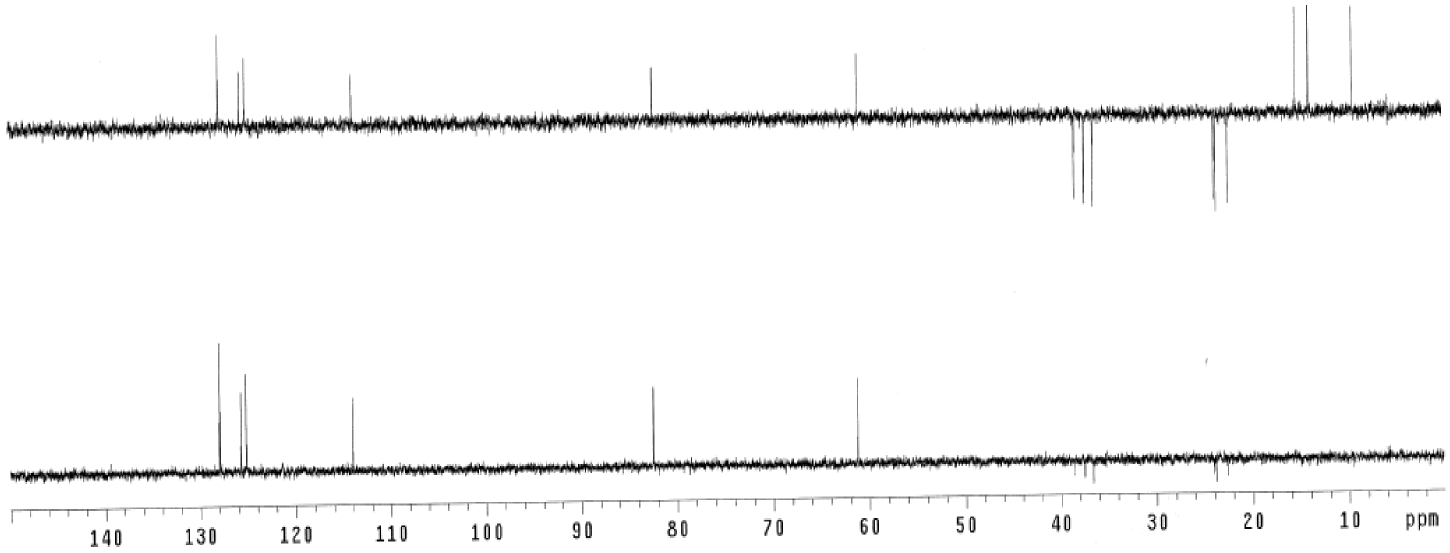

Figure S4. DEPT spectrum of **1**.

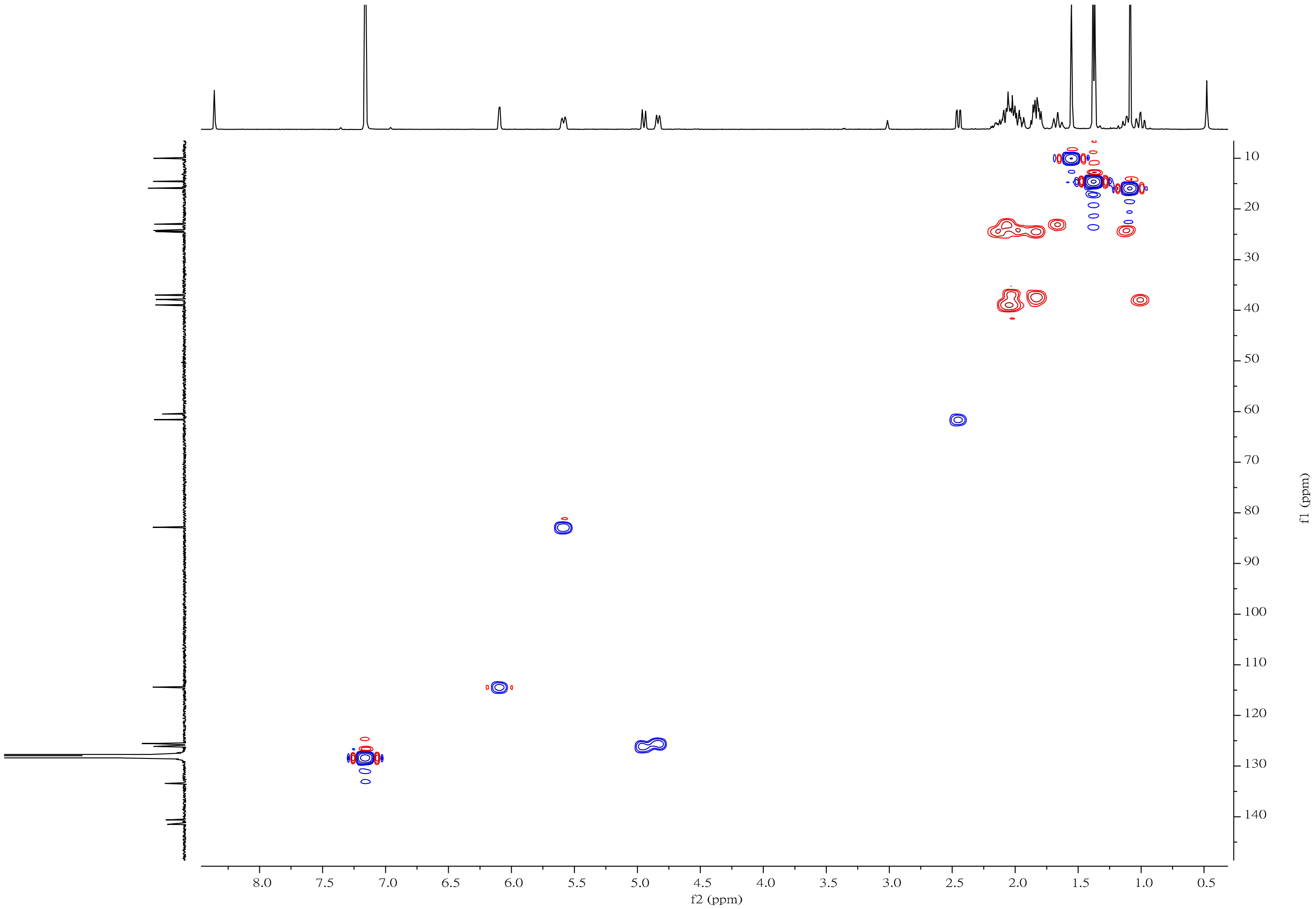

Figure S5. HSQC spectrum of **1**.

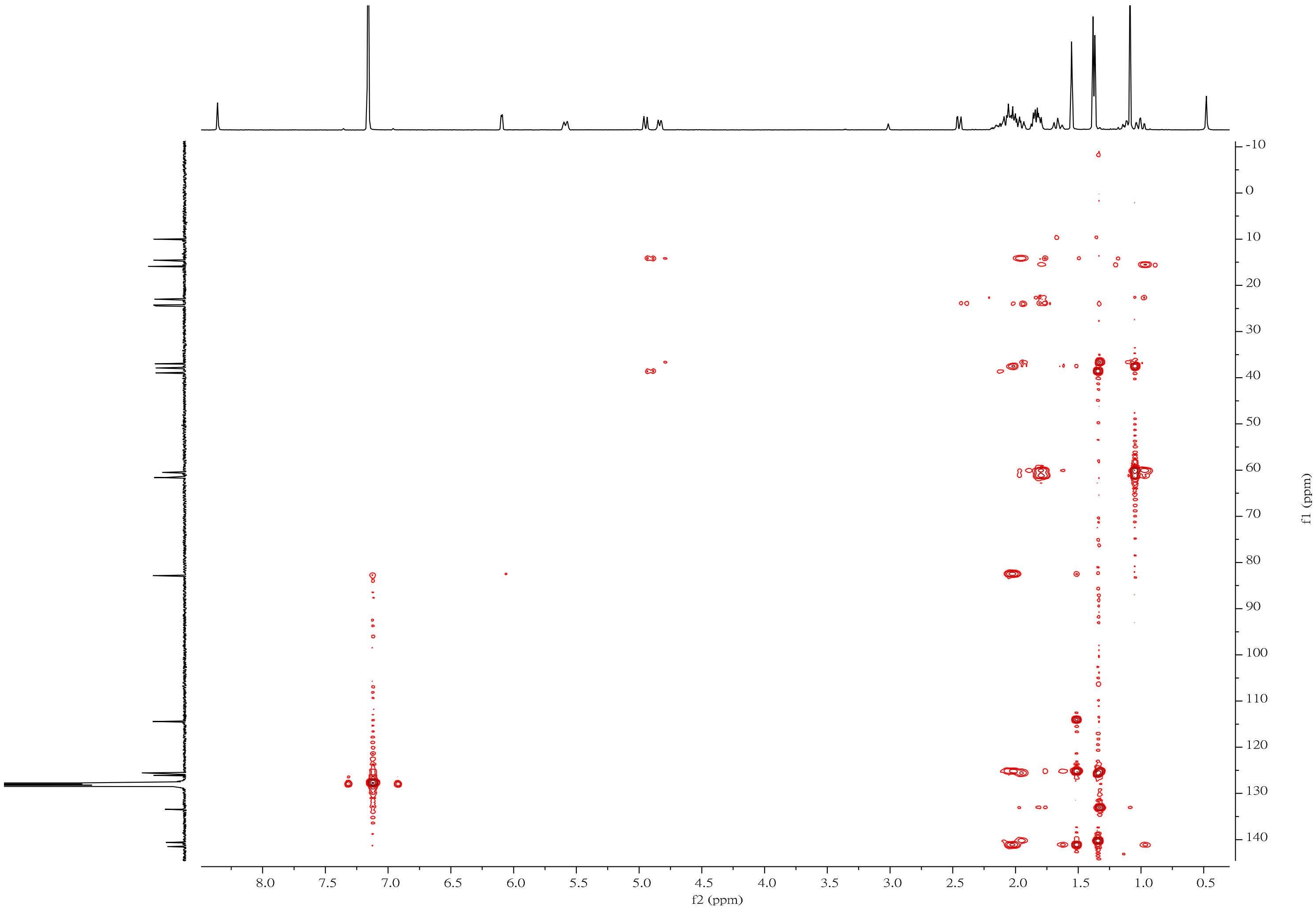

Figure S6. HMBC spectrum of **1**.

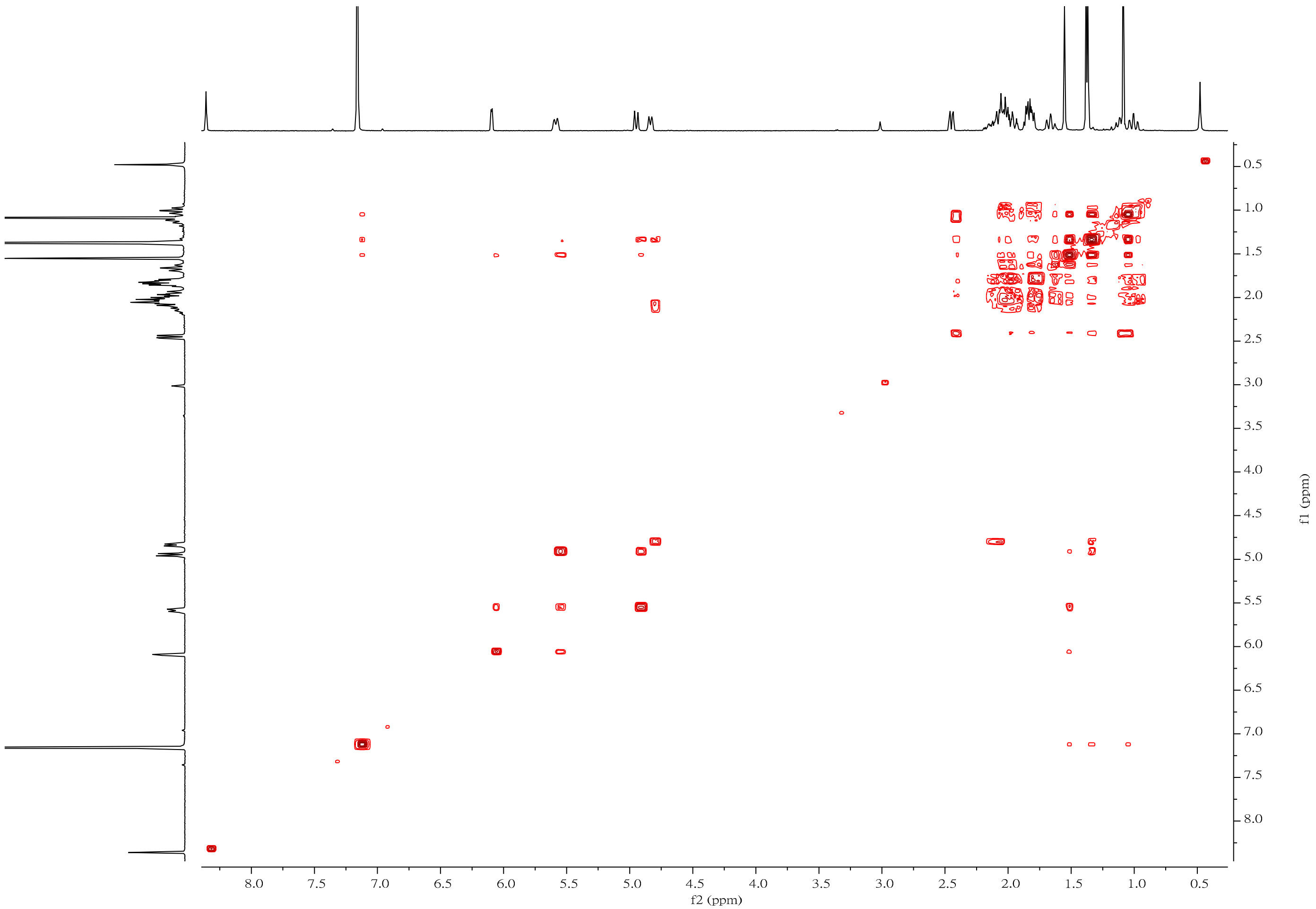

Figure S7. COSY spectrum of **1**.

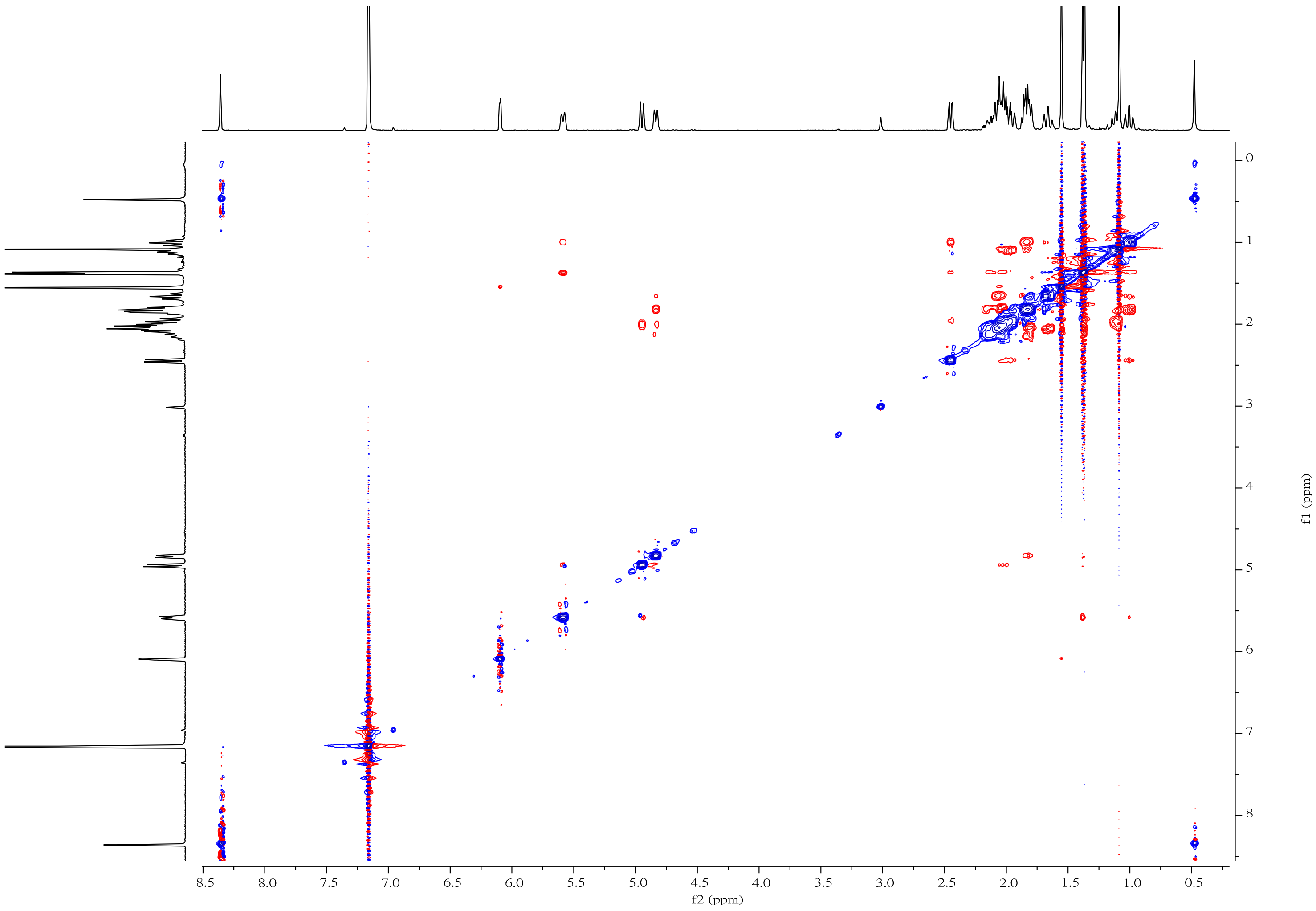

Figure S8. NOESY spectrum of **1**.

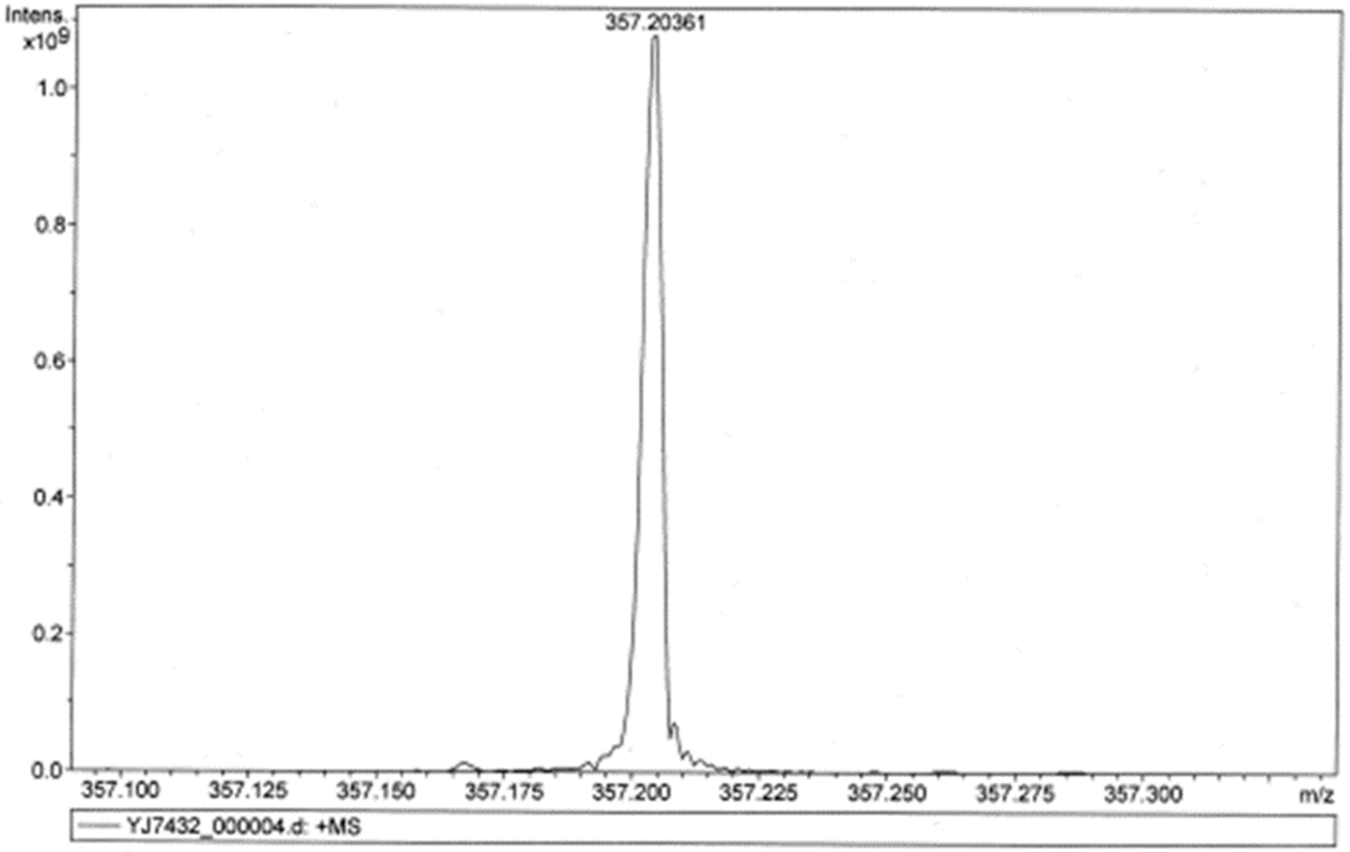

Figure S9. (+)-HRESIMS spectrum of **2**.

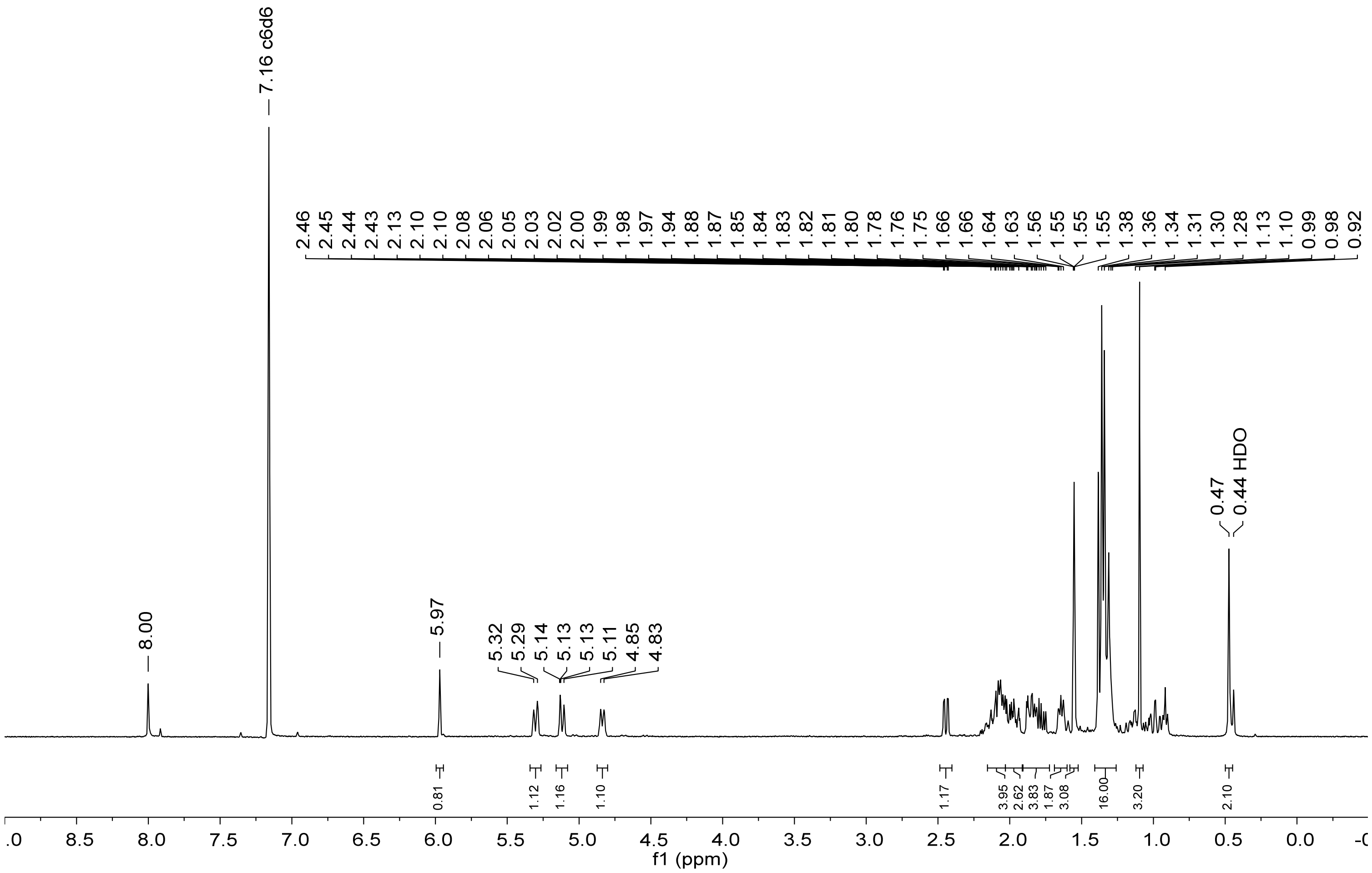

Figure S10. ^{1}H NMR spectrum of **2**.

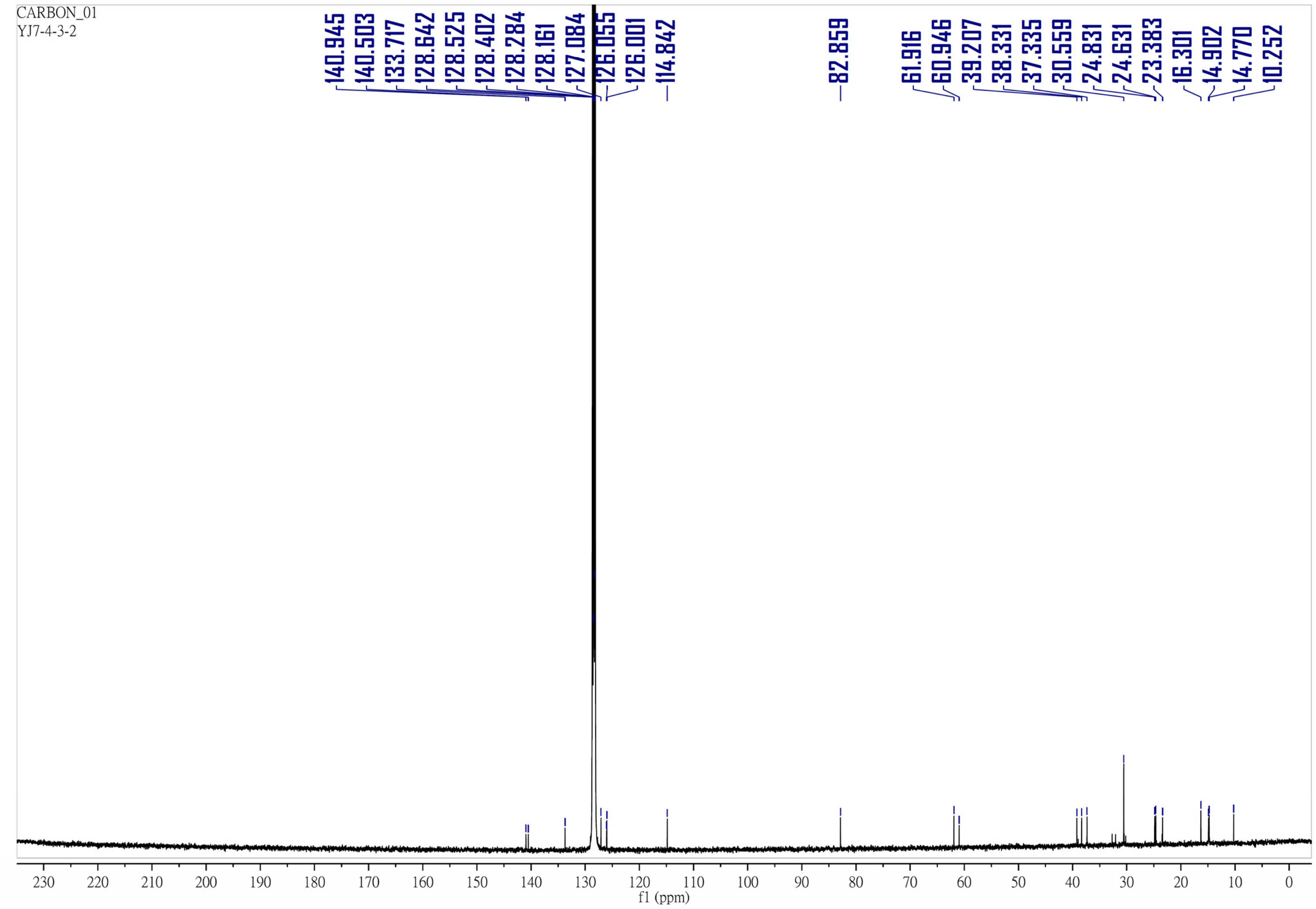

Figure S11. ^{13}C NMR spectrum of **2**.

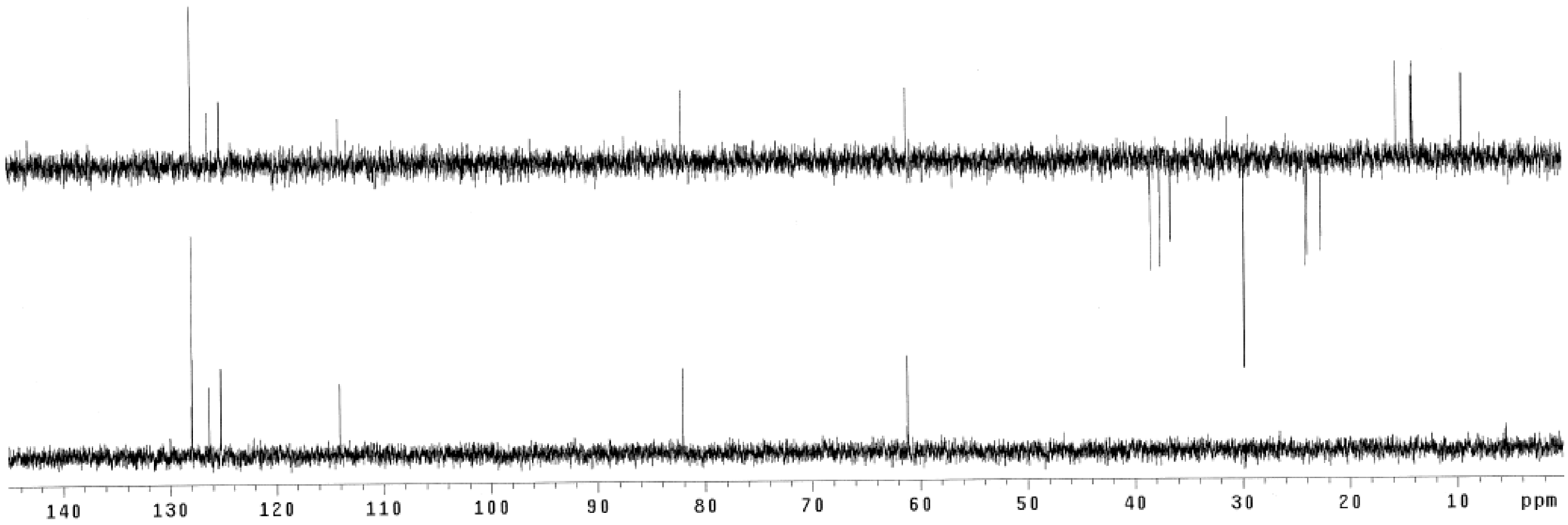

Figure S12. DEPT spectrum of **2**.

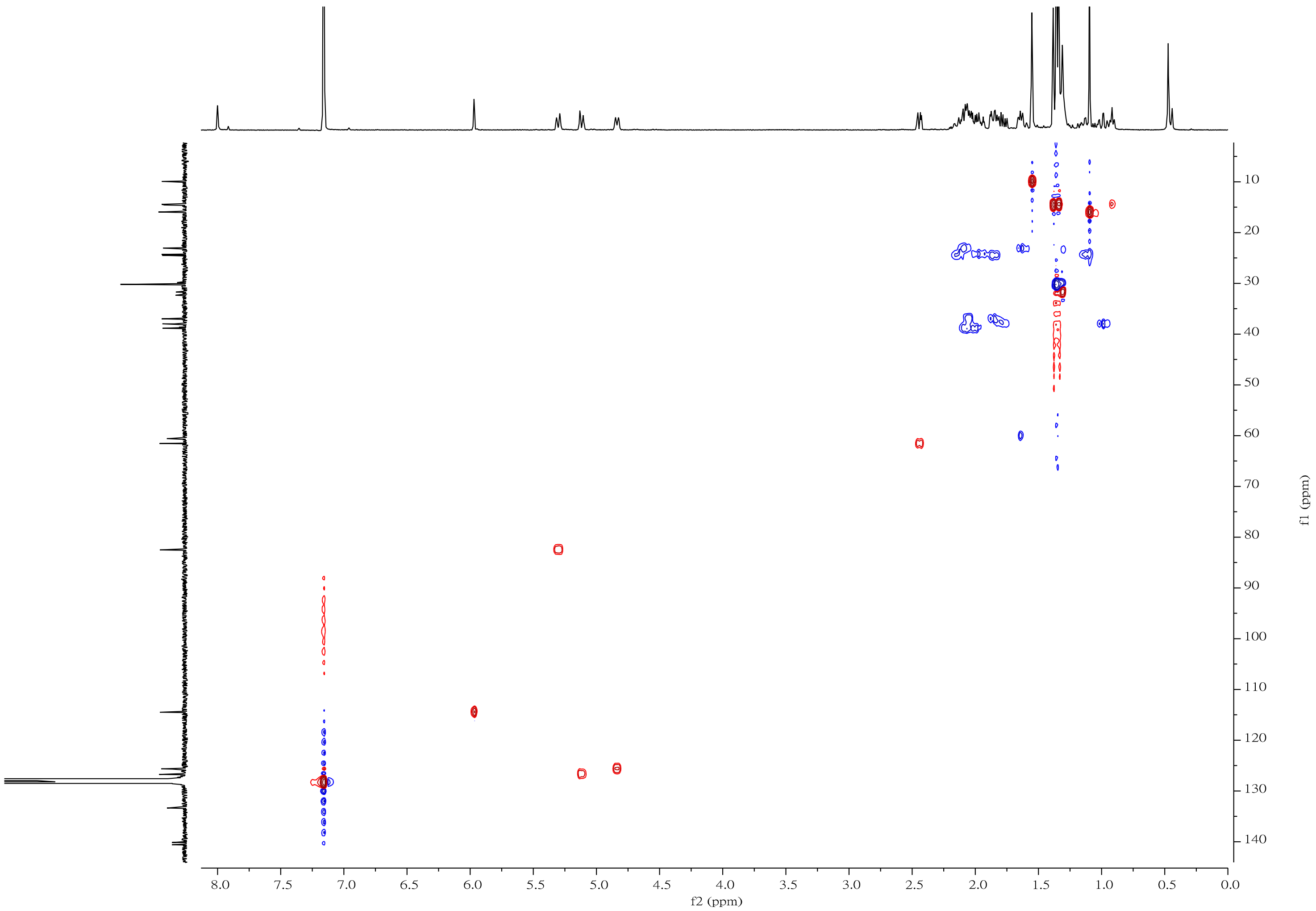

Figure S13. HSQC spectrum of **2**.

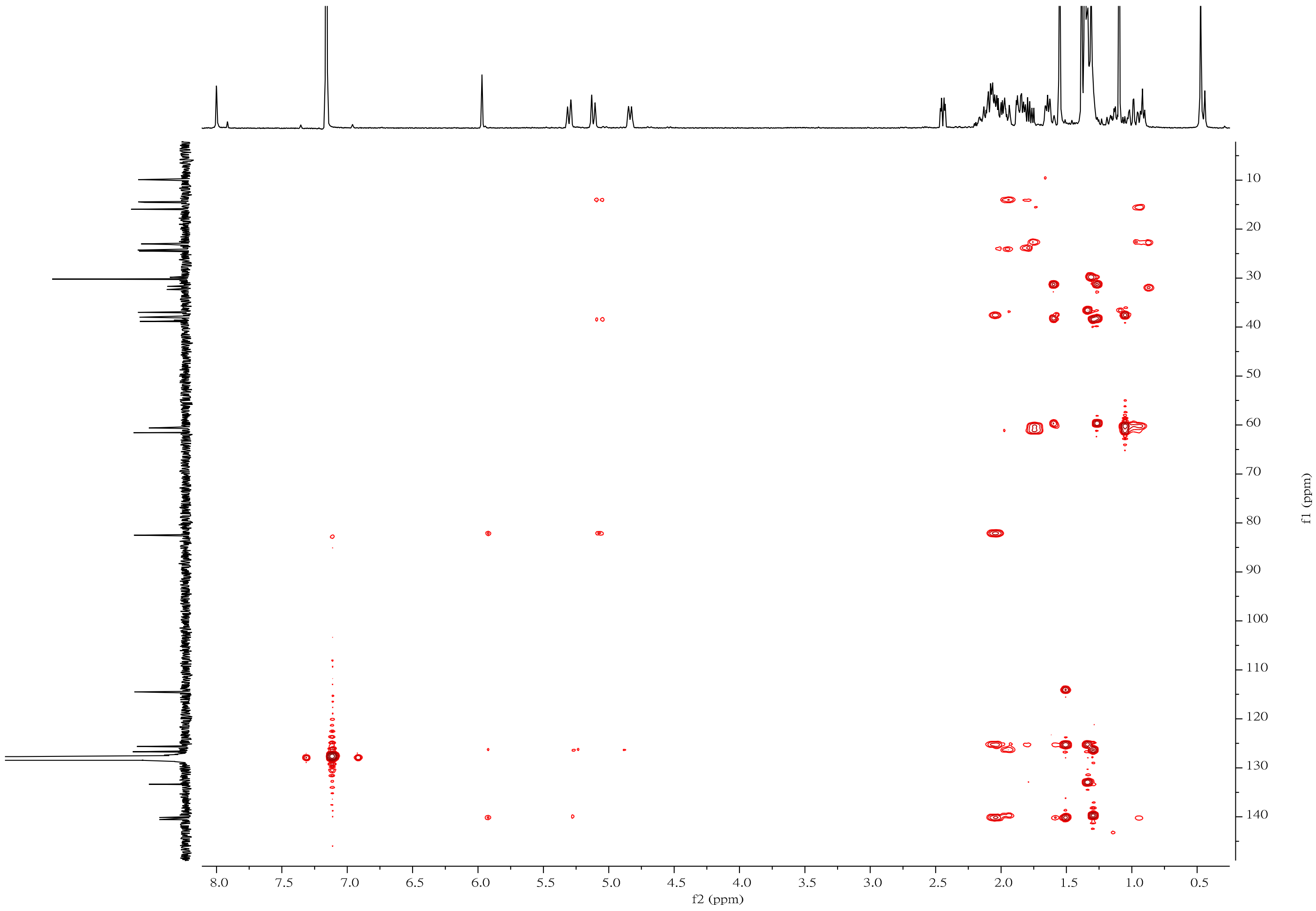

Figure S14. HMBC spectrum of **2**.

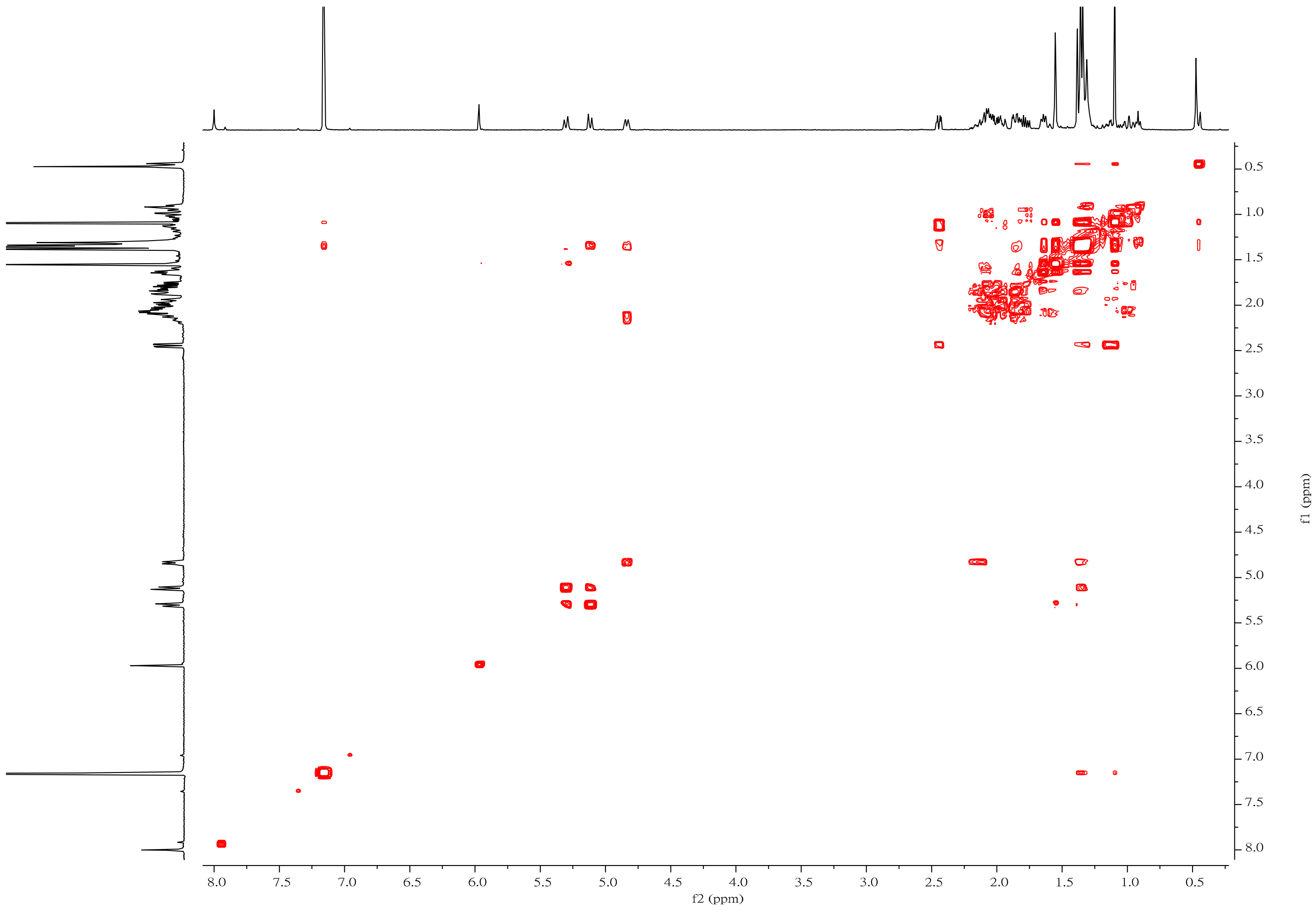

Figure S15. COSY spectrum of **2**.

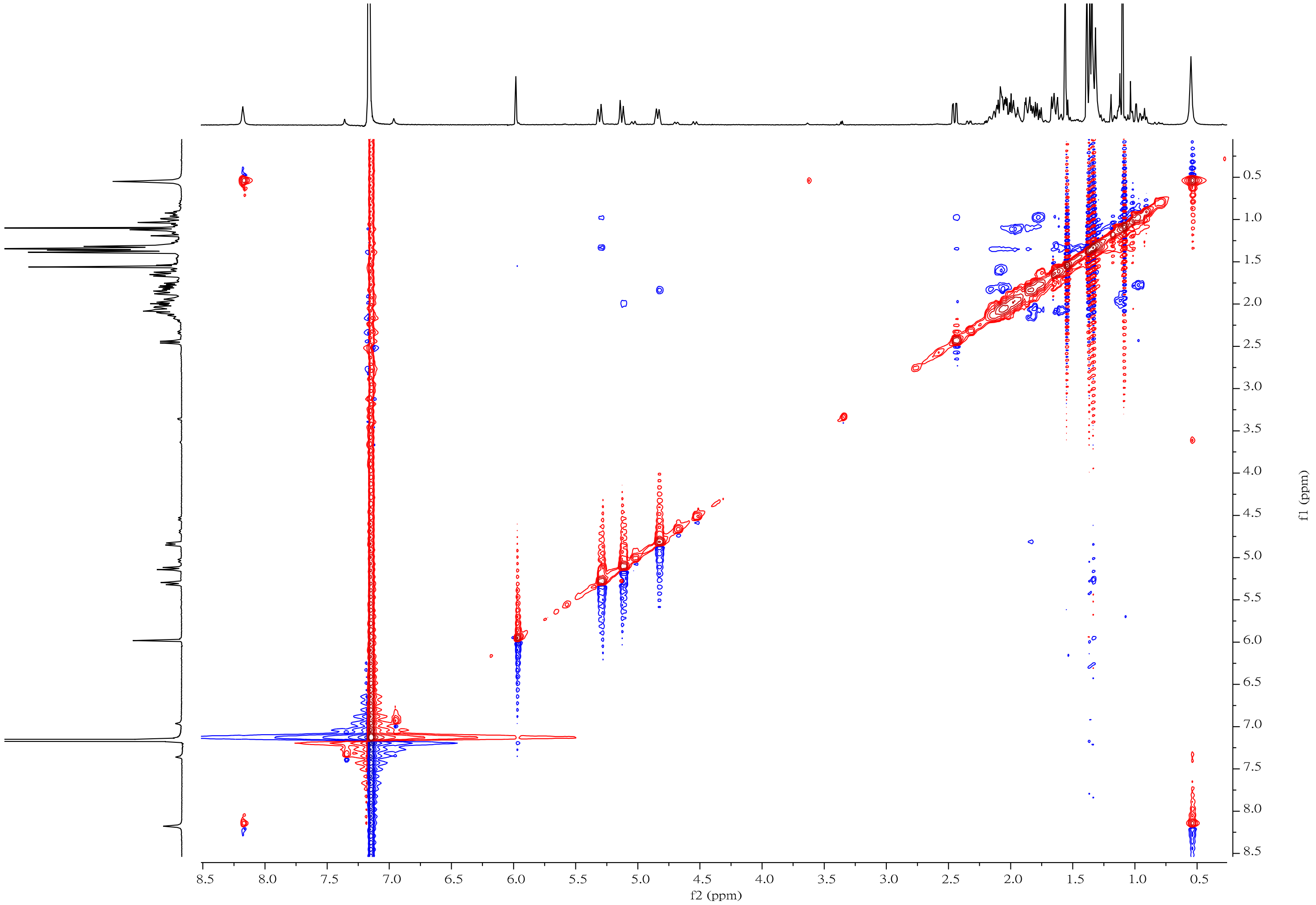

Figure S16. NOESY spectrum of **2**.

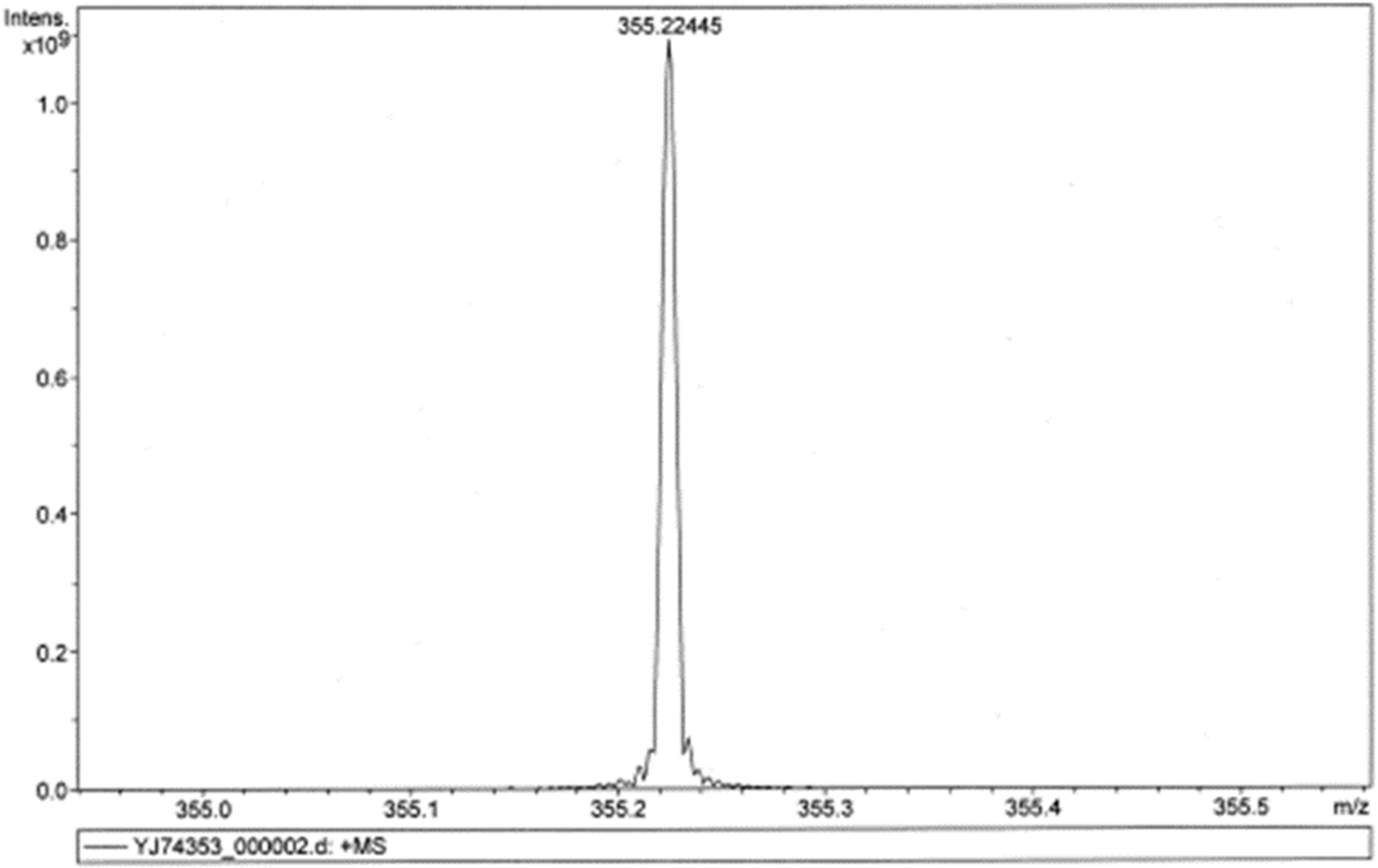

Figure S17. (+)-HRESIMS spectrum of **3**.

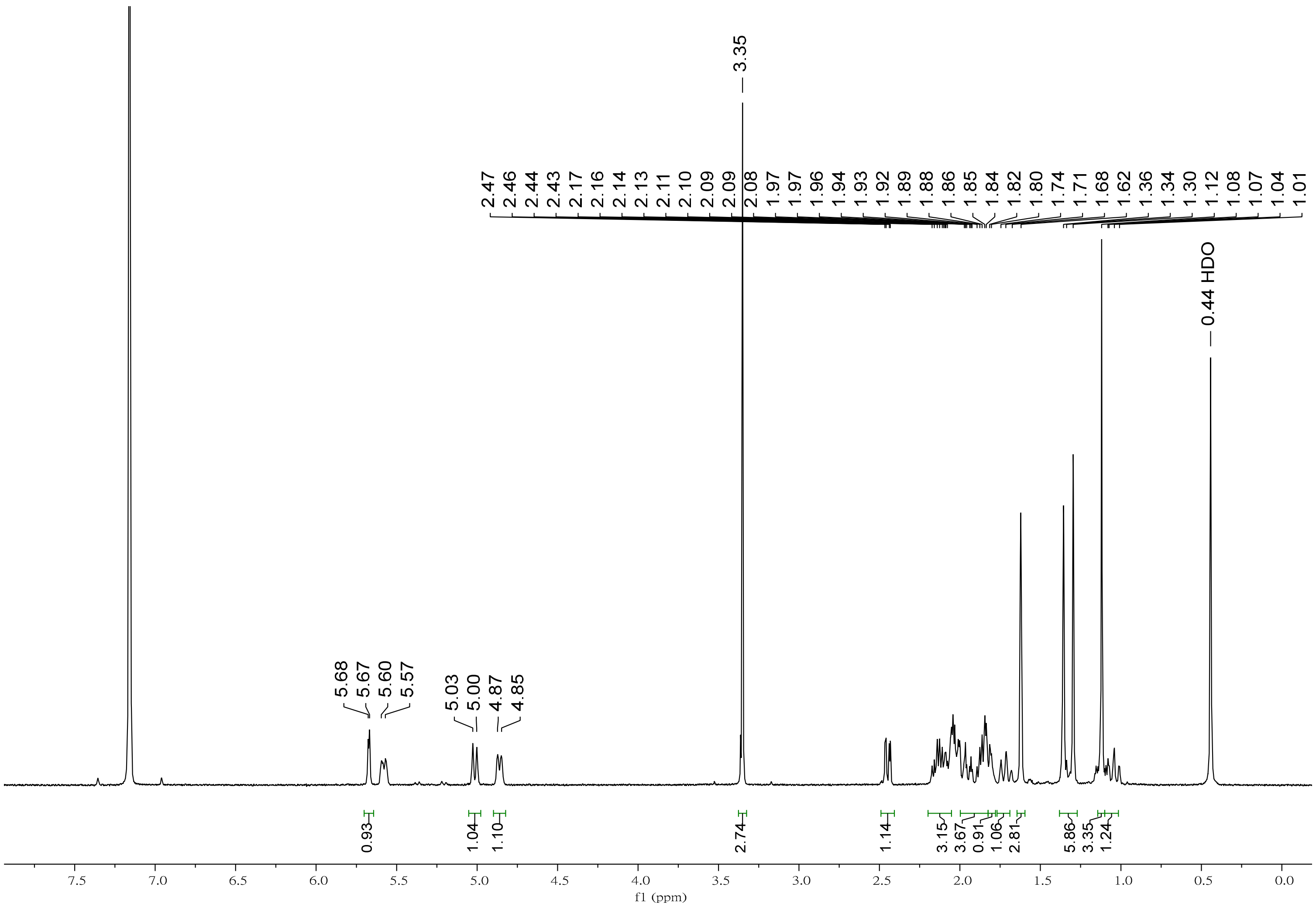

Figure S18. ^{1}H NMR spectrum of **3**.

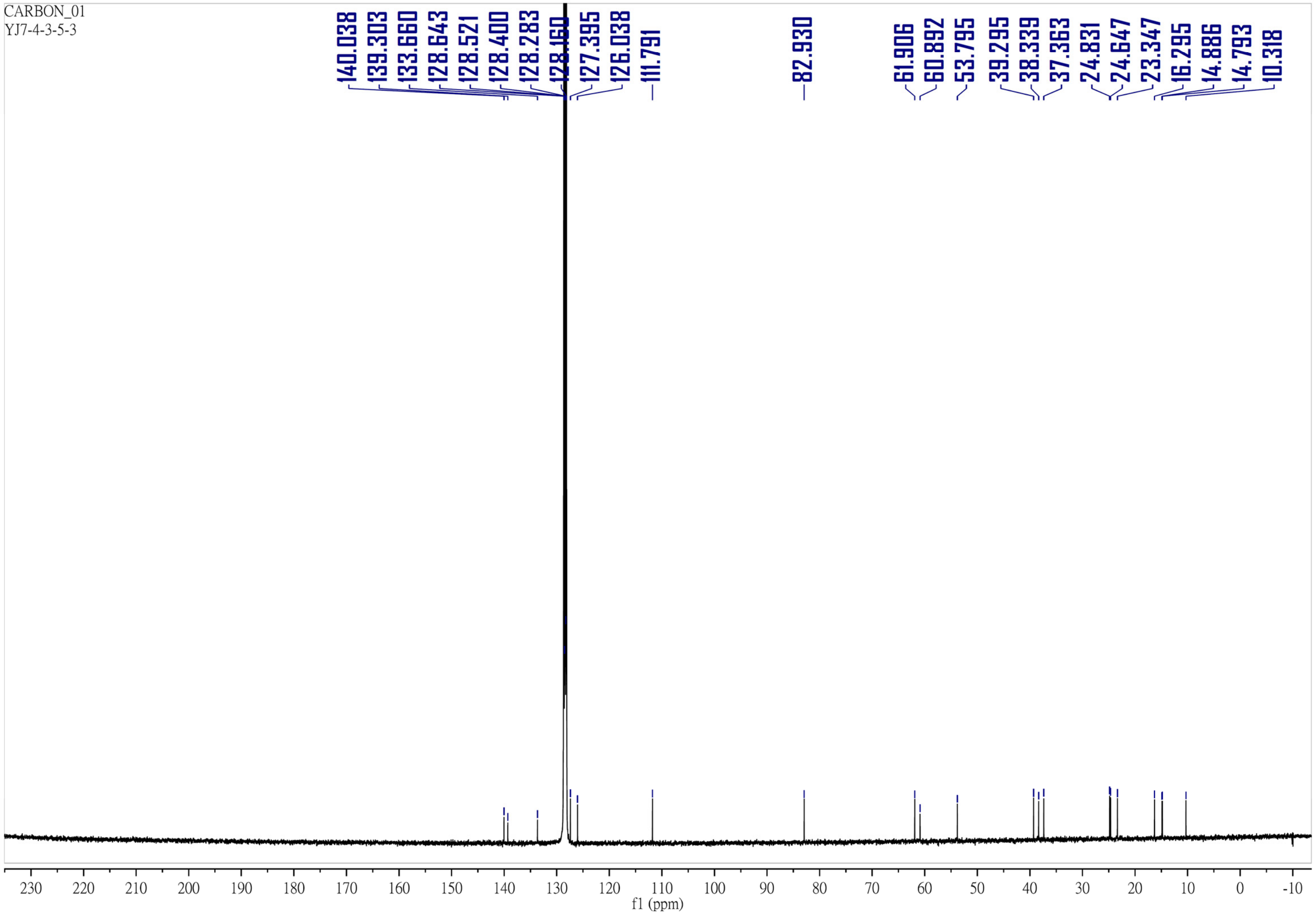

Figure S19. ^{13}C NMR spectrum of **3**.

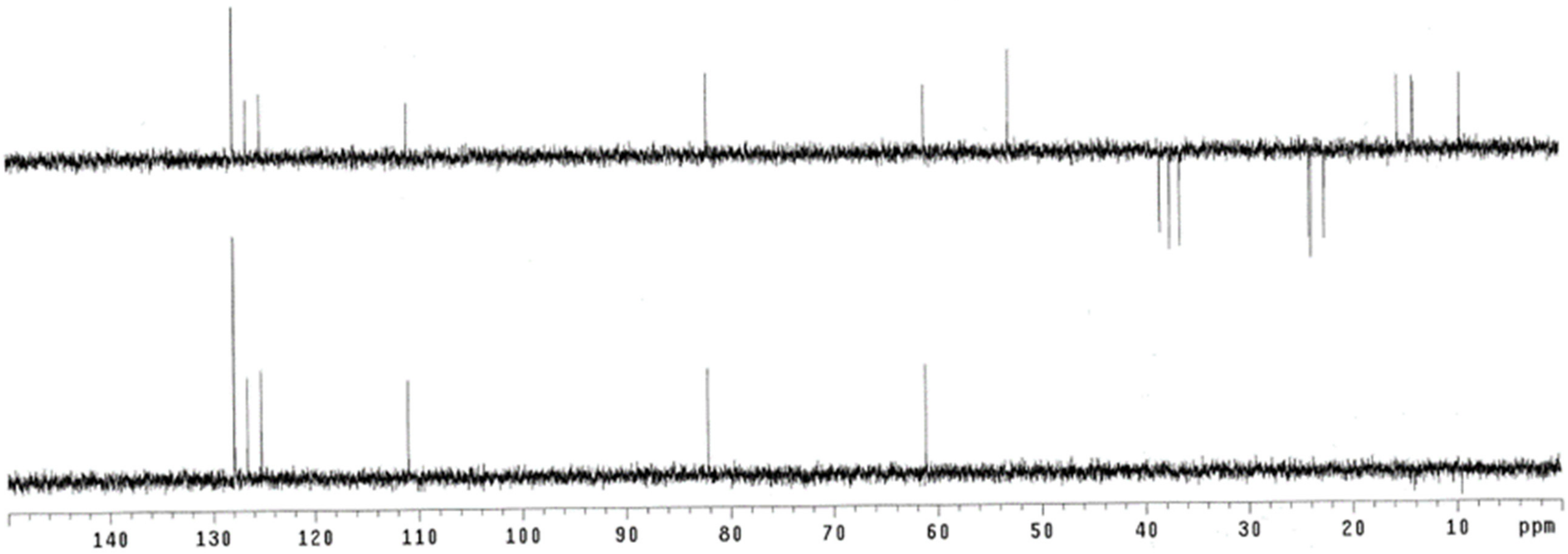

Figure S20. DEPT spectrum of **3**.

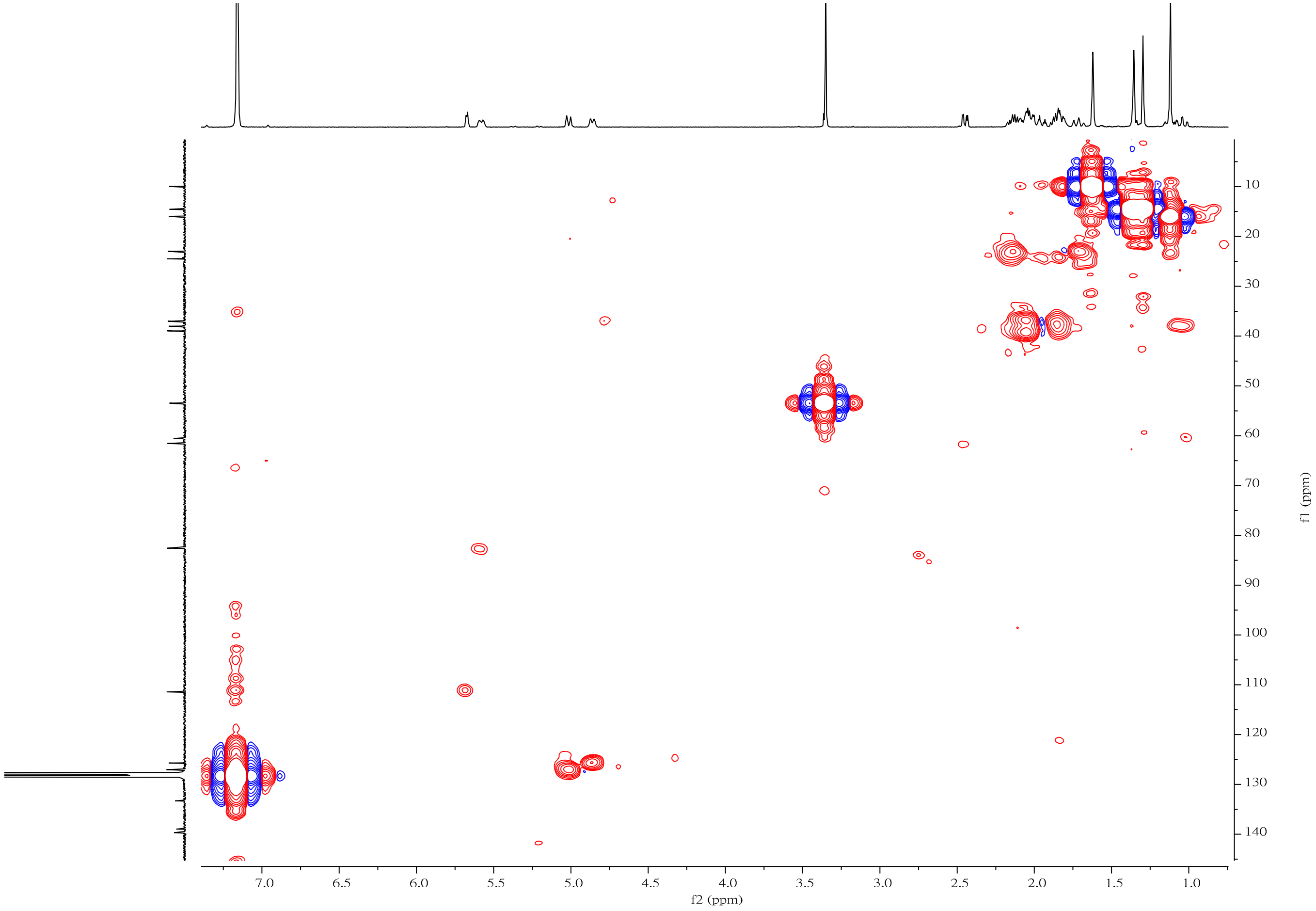

Figure S21. HSQC spectrum of **3**.

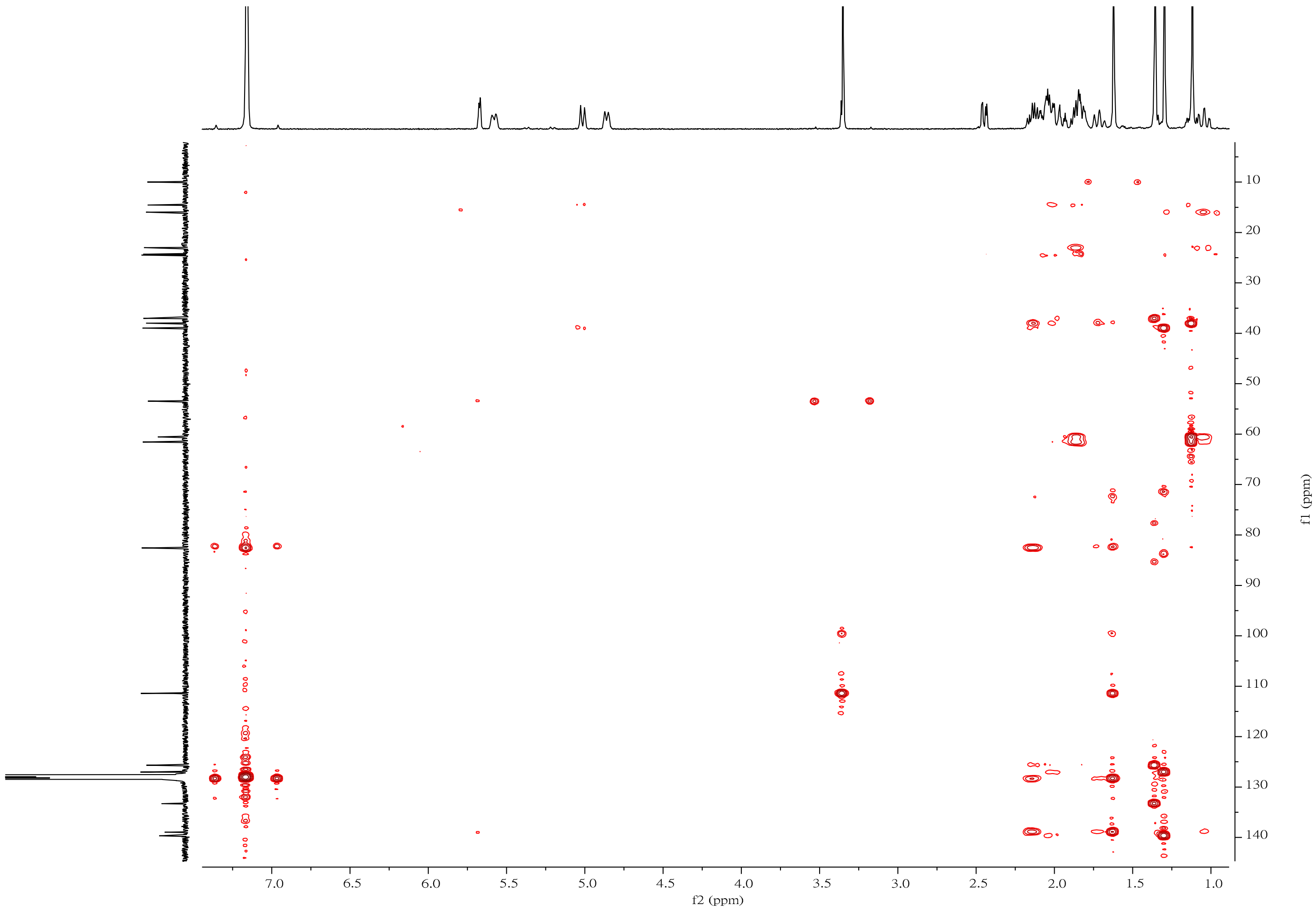

Figure S22. HMBC spectrum of **3**.

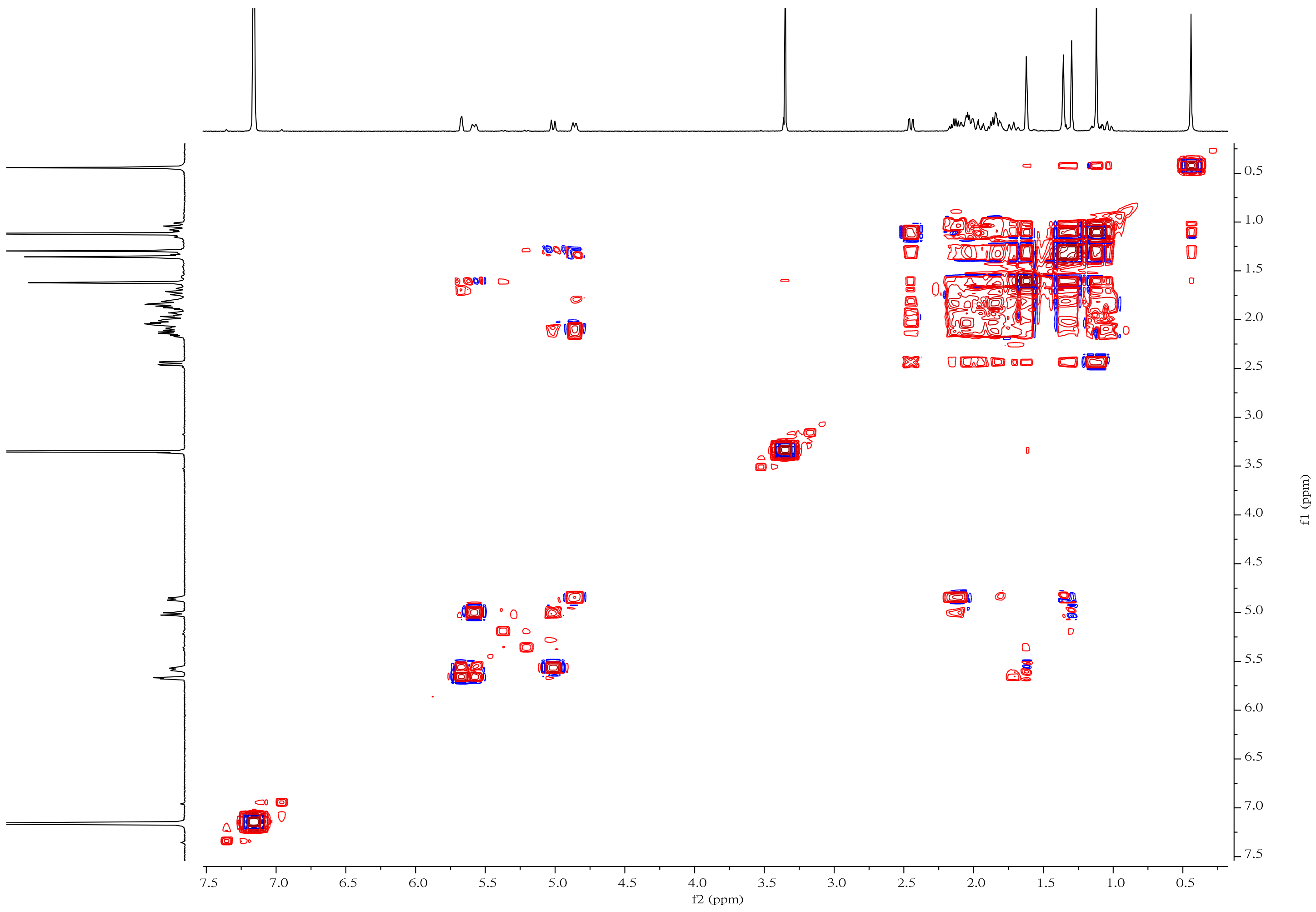

Figure S23. COSY spectrum of **3**.

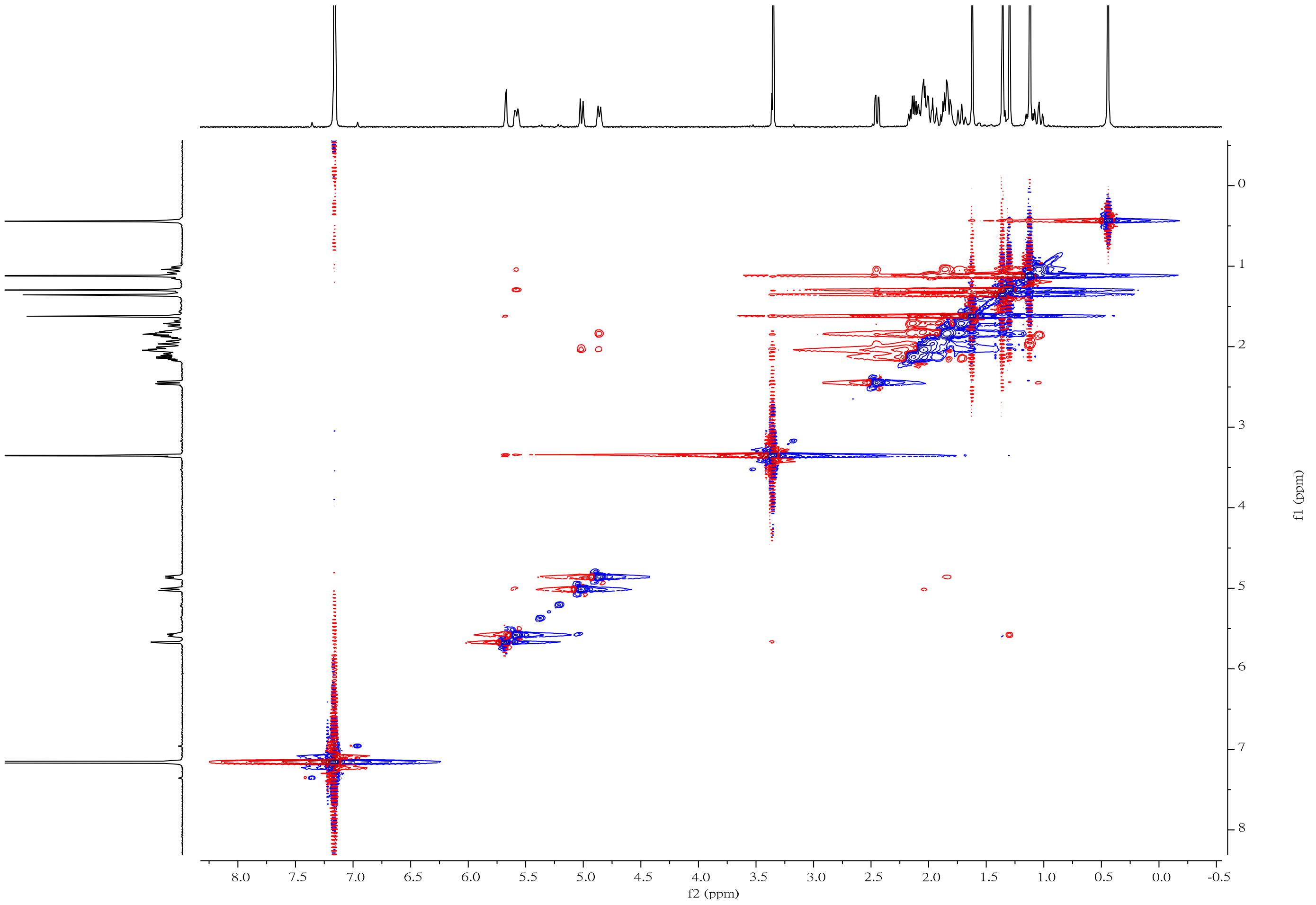

Figure S24. NOESY spectrum of **3**.

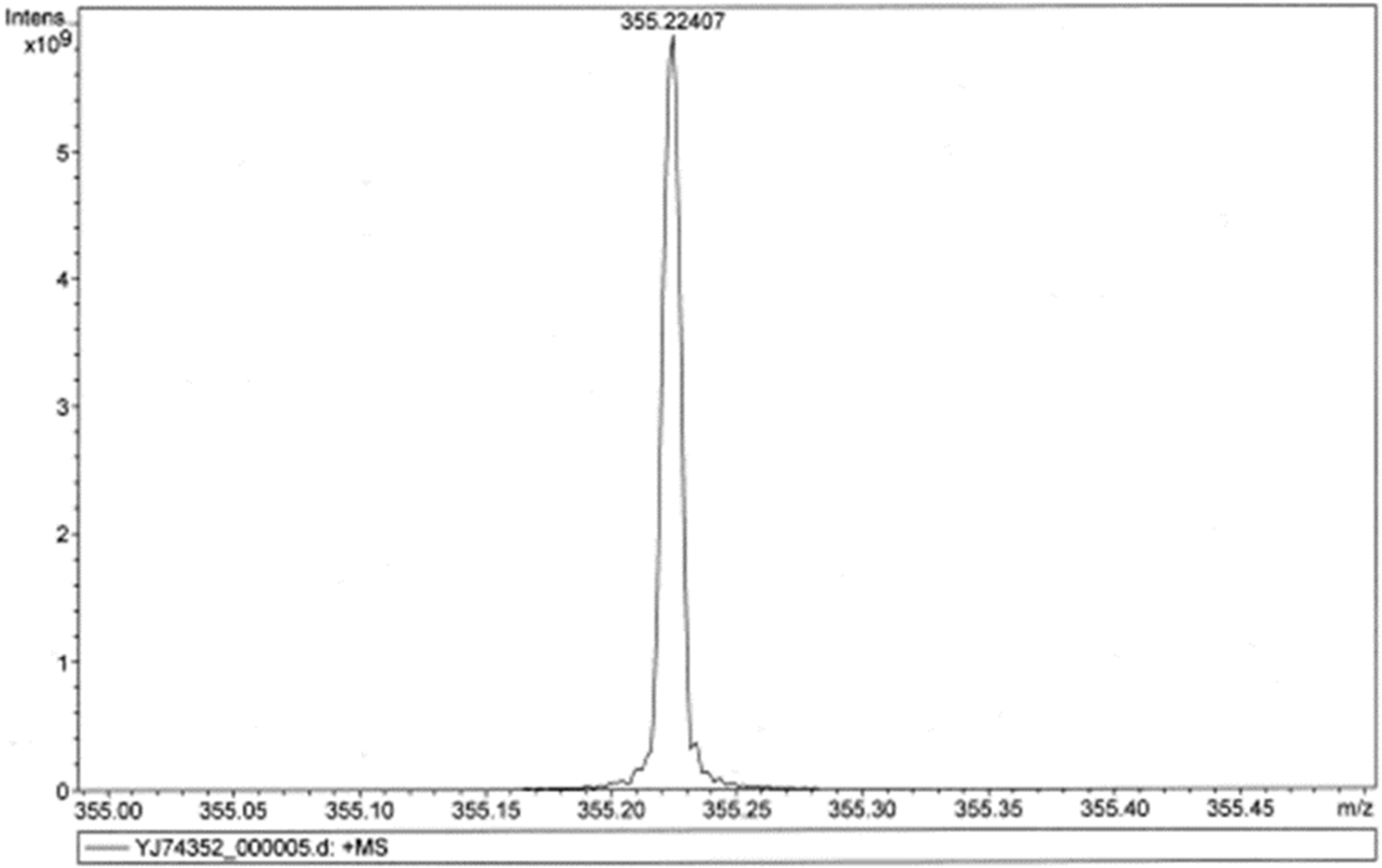

Figure S25. (+)-HRESIMS spectrum of **4**.

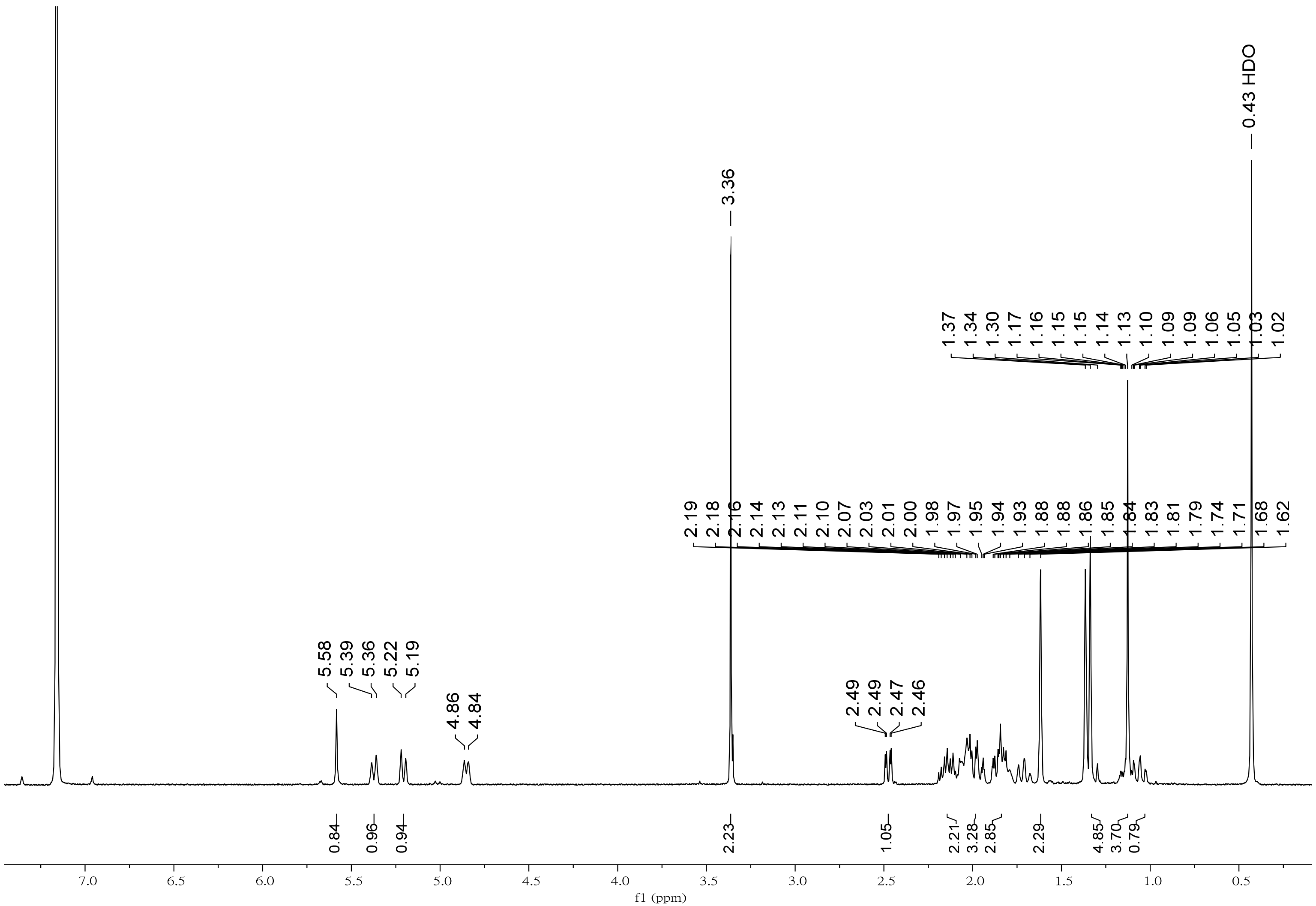

Figure S26. ^{1}H NMR spectrum of **4**.

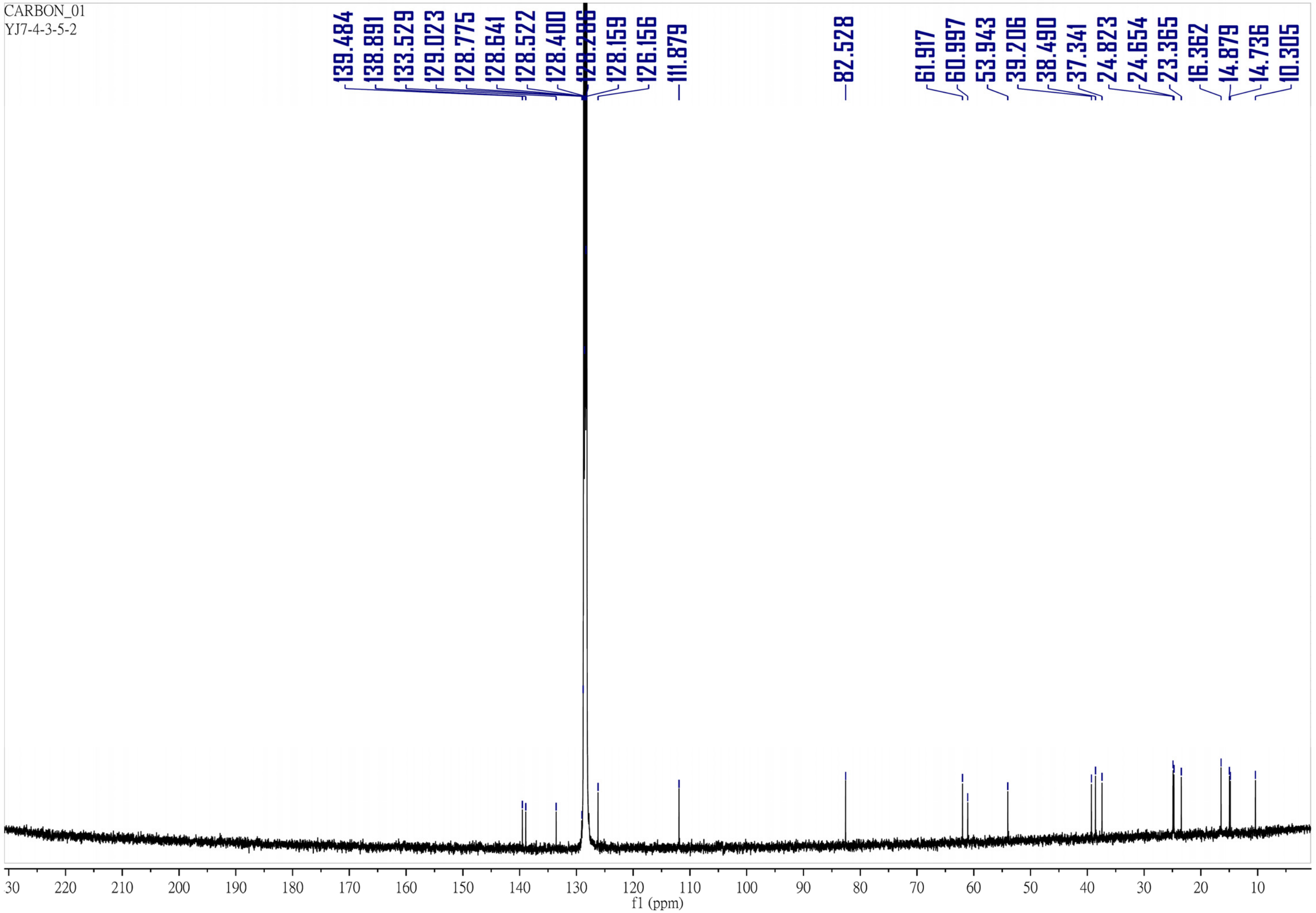

Figure S27. ^{13}C NMR spectrum of **4**.

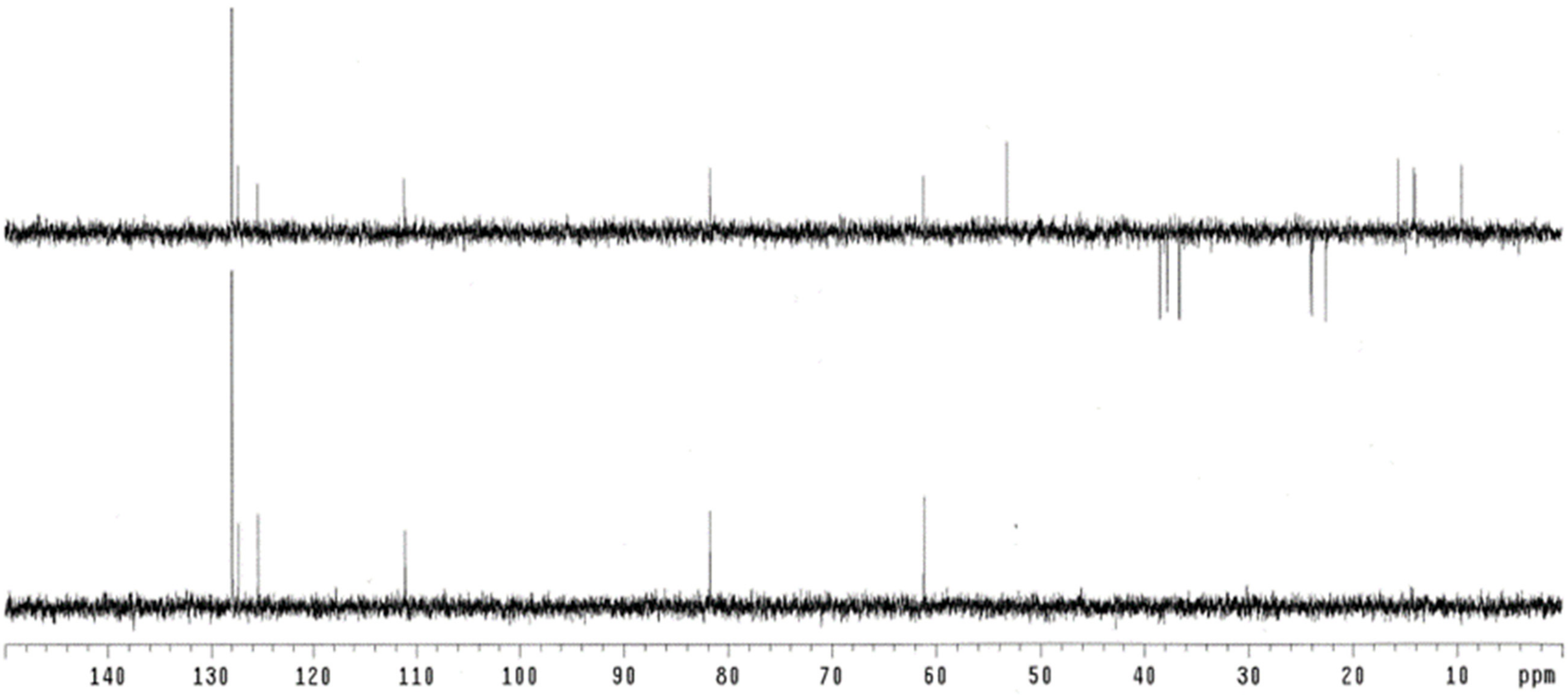

Figure S28. DEPT spectrum of **4**.

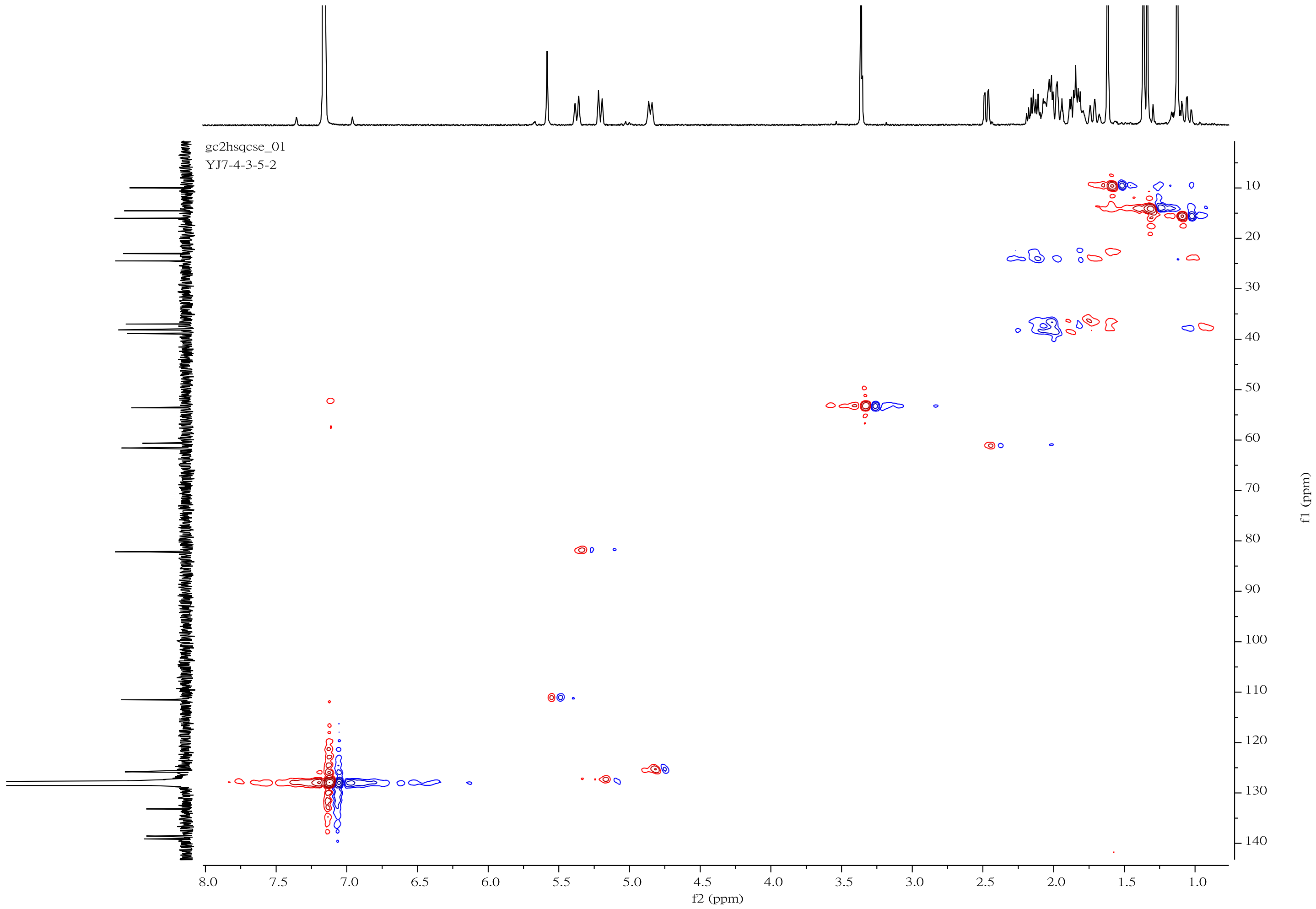

Figure S29. HSQC spectrum of **4**.

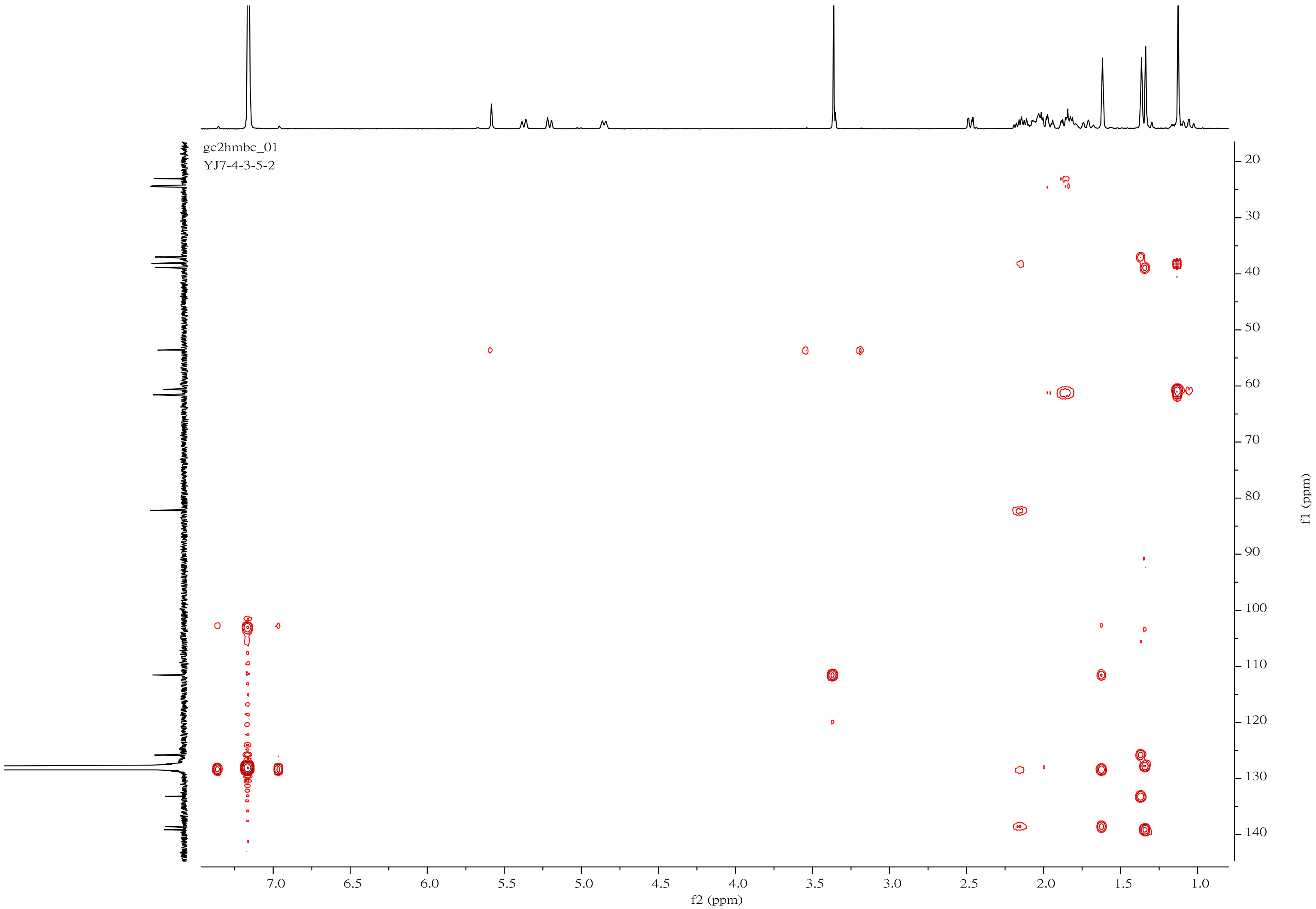

Figure S30. HMBC spectrum of **4**.

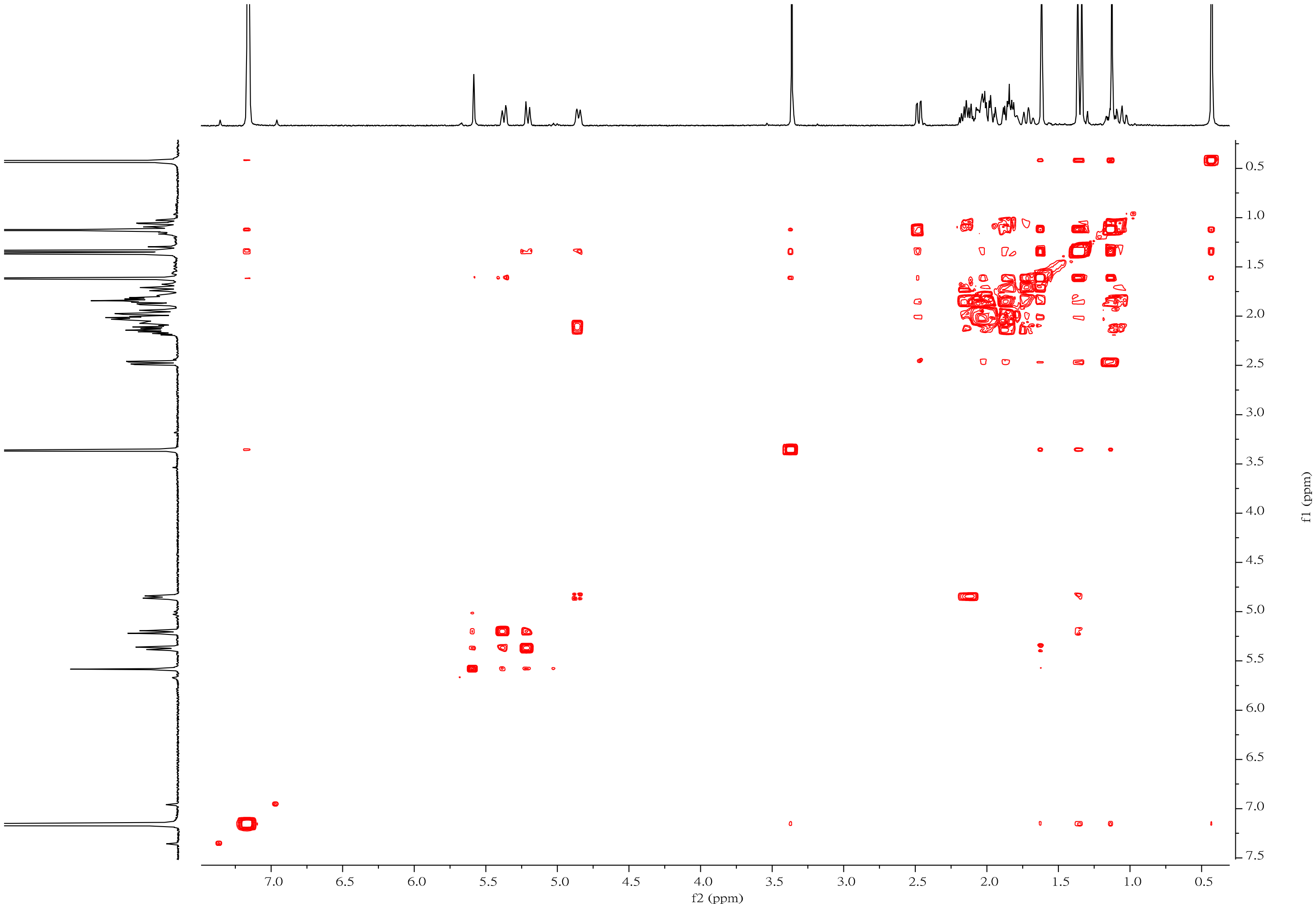

Figure S31. COSY spectrum of **4**.

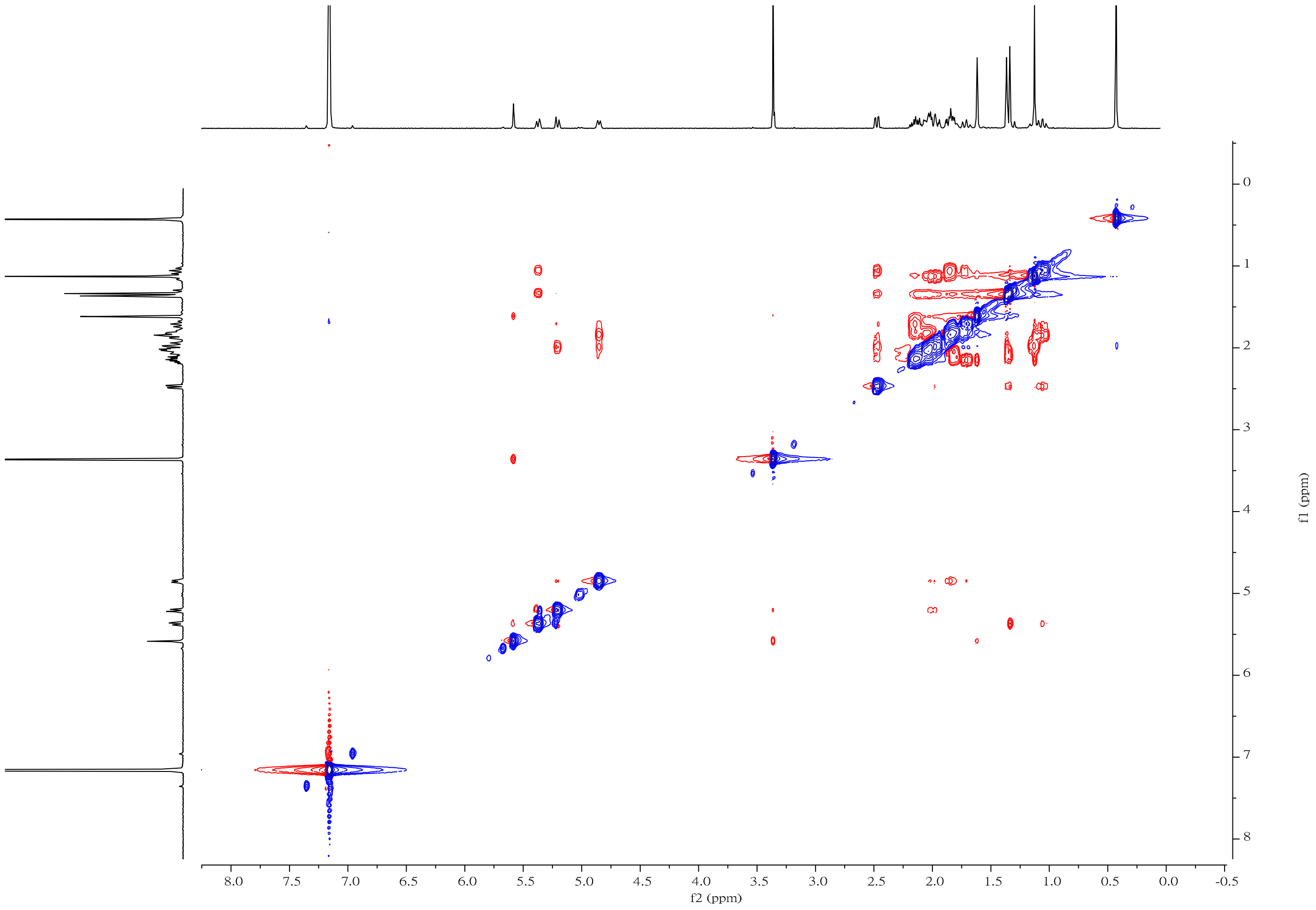

Figure S32. NOESY spectrum of **4**.

Table S1. Conformers and Boltzmann populations of compound **1**.

1a (46.01%)

C	2.846608	-1.60779	-1.0185
C	3.745778	-0.81057	-0.41779
C	1.006842	-3.34333	-0.74593
C	2.520789	-3.03952	-0.68417
C	-0.90559	-1.80631	-0.45
C	0.150716	-2.42597	0.101106
C	3.954397	0.616056	-0.88665
C	3.771472	1.68088	0.2164
C	2.38533	1.634017	0.806654
C	1.261715	2.508516	0.418462
C	-0.14233	1.958857	0.583724
C	-0.65852	1.296207	-0.71197
C	-1.9048	0.499289	-0.49187
C	-1.85078	-0.84774	0.205611
C	4.625419	-1.24398	0.73071
C	0.572497	-2.2933	1.541978
O	1.96792	2.717857	1.669104
C	-3.16756	0.789111	-0.83405
C	-4.0612	-0.31806	-0.33782
O	-3.20743	-1.35589	0.087856
H	2.105834	0.654086	1.198199
C	-3.72695	1.987365	-1.53153
C	1.419551	3.61574	-0.59996
O	-4.79662	0.231787	0.760657
O	-5.78825	-0.75332	1.167944
H	2.273884	-1.18646	-1.84611
H	0.666728	-3.29371	-1.78626
H	0.856786	-4.37993	-0.41182
H	3.020846	-3.71288	-1.39497
H	2.904673	-3.30441	0.304529
H	-1.11929	-1.97711	-1.50478
H	4.974764	0.716629	-1.28257
H	3.26907	0.832774	-1.7142
H	3.979821	2.671334	-0.1955
H	4.500258	1.52099	1.020019

1b (34.35%)

C	2.887309	-1.52174	-0.99446
C	3.732107	-0.64496	-0.42645
C	1.168212	-3.36327	-0.64053
C	2.660006	-2.95978	-0.60949
C	-0.8343	-1.9379	-0.38066
C	0.26376	-2.46928	0.180929
C	3.841347	0.775779	-0.94466
C	3.596201	1.863388	0.123746
C	2.219126	1.747453	0.725624
C	1.042293	2.54513	0.329681
C	-0.32349	1.9116	0.513435
C	-0.79027	1.174002	-0.76002
C	-1.97407	0.292299	-0.51894
C	-1.82492	-1.00626	0.24749
C	4.643744	-0.97757	0.73081
C	0.689809	-2.25623	1.611037
O	1.743579	2.821311	1.570402
C	-3.24879	0.4564	-0.88975
C	-4.06365	-0.70399	-0.38433
O	-3.15132	-1.59765	0.209663
H	2.001139	0.760675	1.138464
C	-3.89952	1.57728	-1.63263
C	1.125604	3.640467	-0.70996
O	-5.1088	-0.34354	0.525935
O	-4.5342	0.396571	1.63885
H	2.283666	-1.16875	-1.83208
H	0.81526	-3.376	-1.6777
H	1.089412	-4.39364	-0.26531
H	3.194901	-3.62243	-1.30482
H	3.071616	-3.16609	0.38196
H	-1.0475	-2.16433	-1.42517
H	4.849893	0.930286	-1.35332
H	3.137406	0.917923	-1.77276
H	3.738658	2.850621	-0.32252
H	4.338207	1.777323	0.926543

1c (2.54%)

C	2.928724	2.035501	0.340503
C	3.438323	0.852799	0.724629
C	0.852608	3.509904	0.138656
C	1.942693	2.912251	1.062774
C	-1.05368	1.936348	0.092455
C	-0.00116	2.462949	-0.55311
C	4.429701	0.131016	-0.16723
C	4.021339	-1.30973	-0.54989
C	2.602294	-1.37338	-1.0737
C	1.535756	-2.26045	-0.56004
C	0.096034	-1.79635	-0.68352
C	-0.38632	-1.07723	0.595439
C	-1.73932	-0.458	0.4428
C	-1.93044	0.812713	-0.36707
C	3.122031	0.172335	2.03234
C	0.438968	2.072826	-1.94082
O	2.192594	-2.54259	-1.81982
C	-2.91533	-0.86181	0.943075
C	-3.98809	0.077683	0.455394
O	-3.32034	1.16437	-0.14469
H	2.2637	-0.44718	-1.54077
C	-3.24693	-2.04649	1.792898
C	1.8034	-3.28722	0.518536
O	-4.7484	-0.65969	-0.50792
O	-5.89178	0.156801	-0.88948
H	3.274718	2.435888	-0.61391
H	1.334657	4.146849	-0.61351
H	0.215015	4.161548	0.745993
H	2.475482	3.754182	1.529538
H	1.451189	2.366295	1.873248
H	-1.29282	2.29765	1.092818
H	5.406774	0.081685	0.333213
H	4.574226	0.716349	-1.08232
H	4.700742	-1.67166	-1.33047
H	4.148461	-1.97977	0.305143

H -0.82527 2.763245 0.883057

H -0.13035 1.229814 1.399809

H 0.12349 0.629034 -1.0968

H -0.83494 2.060378 -1.47417

H -1.61403 -0.72244 1.268468

H 4.345078 -0.73834 1.663467

H 4.588381 -2.31879 0.911769

H 5.670042 -0.97355 0.532456

H 1.517271 -1.74217 1.615549

H 0.748414 -3.28416 1.977998

H -0.16434 -1.77859 2.160284

H -4.78406 -0.7251 -1.05246

H -2.93762 2.67157 -1.85195

H -4.30388 1.690376 -2.41584

H -4.40937 2.536492 -0.87341

H 0.685182 4.405793 -0.40778

H 2.414021 4.06086 -0.55348

H 1.254395 3.240178 -1.61526

H -5.24101 -1.36628 1.690808

1d (2.80%)

C 2.302323 -1.37 -1.4426

C 3.479946 -0.89094 -1.00895

C 1.309647 -3.04401 0.194971

C 1.781282 -2.77496 -1.24811

C -0.71865 -1.59585 -0.07346

C 0.185491 -2.17541 0.731419

C 3.827398 0.574747 -1.16515

C 3.857962 1.344161 0.176959

C 2.513194 1.300504 0.858531

C 1.457287 2.322926 0.723254

C 0.019688 1.857333 0.865689

C -0.57753 1.457439 -0.50025

C -1.8281 0.642819 -0.40168

C -1.8327 -0.68376 0.338325

C 4.510896 -1.73141 -0.29558

C 0.192515 -2.05827 2.235286

O 2.246164 2.22707 1.937748

H -1.05916 2.677594 0.78695

H -0.26402 1.211758 1.352627

H 0.039676 0.554864 -1.12569

H -1.02059 1.899331 -1.54555

H -1.57125 -0.81004 1.295689

H 4.330471 -0.46393 1.648621

H 4.684559 -2.04594 0.945959

H 5.66568 -0.63938 0.518883

H 1.606176 -1.65628 1.65427

H 0.918362 -3.21889 2.084462

H -0.06746 -1.75497 2.215514

H -4.65719 -1.21731 -1.15456

H -3.16901 2.314247 -1.97471

H -4.44815 1.205203 -2.5066

H -4.62722 2.088452 -0.9919

H 0.350234 4.391202 -0.52147

H 2.093884 4.141696 -0.68492

H 0.968571 3.237857 -1.71612

H -4.30335 -0.33154 2.241276

1e (7.11%)

C -3.04127 -1.52013 0.897882

C -3.81622 -0.89724 -0.00652

C -0.96994 -2.99903 1.152824

C -2.48038 -2.91405 0.844946

C 0.970324 -1.56412 0.598851

C -0.04982 -2.31933 0.159207

C -4.35599 0.491301 0.27491

C -3.86087 1.584507 -0.69635

C -2.35306 1.630044 -0.73548

C -1.51494 2.273559 0.289758

C -0.12608 1.725517 0.58878

C 0.712073 1.330941 -0.64022

C 1.968749 0.619346 -0.24063

C 2.031634 -0.89418 -0.22324

C -4.23893 -1.49222 -1.32719

C -0.3373 -2.60502 -1.2927

O -1.69902 2.890783 -1.01338

H 0.021578 -1.12505 -1.54446

H -0.55338 -2.65622 -0.88894

H 0.336633 -0.29153 0.845908

H -0.39912 -1.7827 1.430774

H -1.78663 0.611281 -1.43502

H 4.041239 0.029292 2.615396

H 2.421254 0.73823 2.647716

H 2.69812 -0.82418 1.870298

H 1.448011 1.64729 -1.91771

H 0.488709 2.962433 -2.58042

H -0.22029 1.347728 -2.42038

H -4.68528 0.462559 1.206857

H -3.77731 -1.73864 2.702245

H -3.90632 -2.73738 1.255956

H -2.34885 -2.59352 2.089661

H 1.114333 -4.13088 0.401233

H 2.821848 -3.67253 0.453468

H 1.652905 -2.86809 1.518493

H -5.47981 0.774906 -1.51943

1f (2.61%)

C 2.907984 -1.50161 -0.97547

C 3.736673 -0.61556 -0.39828

C 1.202647 -3.36009 -0.64672

C 2.689109 -2.94091 -0.59072

C -0.82042 -1.95998 -0.41131

C 0.275409 -2.48041 0.164573

C 3.840888 0.80413 -0.92021

C 3.569555 1.893638 0.13968

C 2.185794 1.766898 0.723748

C 1.003917 2.541259 0.297463

C -0.35651 1.895025 0.47658

C -0.81134 1.143399 -0.79317

C -1.9901 0.257326 -0.54457

C -1.83473 -1.05002 0.209029

C 4.634886 -0.93632 0.77267

C 0.67509 -2.26968 1.602538

O 1.683435 2.848923 1.542476

C	-3.03442	0.869766	-0.93963
C	-3.94203	-0.27549	-0.56805
O	-3.11343	-1.27328	-0.01628
H	2.163069	0.290545	1.075243
C	-3.53525	2.022663	-1.74919
C	1.661663	3.592071	-0.0735
O	-4.85292	0.242447	0.4078
O	-5.84879	-0.78577	0.674677
H	1.629312	-0.66994	-1.93382
H	1.007183	-4.09742	0.29255
H	2.171131	-2.92493	0.863365
H	0.965765	-2.96739	-1.95279
H	2.559525	-3.51059	-1.48392
H	-0.65245	-1.74861	-1.14831
H	4.813721	0.672247	-1.63799
H	3.102027	1.055427	-1.83158
H	4.164391	2.378012	-0.00079
H	4.608911	0.90728	0.846486
H	-0.59029	2.644412	1.325578
H	0.009465	1.002229	1.54707
H	-0.75894	2.348596	-1.10689
H	0.174465	0.85848	-1.03076
H	-1.81843	-0.51201	1.420046
H	4.319547	-2.80198	-0.39388
H	5.513549	-1.52646	-0.6899
H	4.542306	-1.49994	0.776771
H	-0.705	-1.59445	2.646411
H	0.293396	-3.05325	2.68571
H	1.061016	-1.47571	2.569196
H	-4.53358	-0.71575	-1.37726
H	-2.75079	2.760781	-1.93172
H	-3.91556	1.681478	-2.71996
H	-4.36505	2.523129	-1.23808
H	1.038518	4.39349	0.338632
H	2.700674	3.921779	-0.04217
H	1.375285	3.446628	-1.12035
H	-5.35507	-1.37586	1.2719

C	3.143756	1.132793	0.146498
C	4.097458	-0.00446	0.405345
O	3.325071	-1.18477	0.367825
H	-1.90548	0.793205	-1.27121
C	3.565968	2.561786	0.271474
C	-2.12344	3.031342	1.449616
O	5.047313	0.02608	-0.66543
O	6.082506	-0.95408	-0.36876
H	-2.8072	-0.97375	1.813411
H	-0.77735	-2.60429	2.156974
H	-0.70134	-4.06559	1.179898
H	-2.99533	-3.52659	1.598884
H	-2.687	-3.38918	-0.11732
H	1.083859	-1.4043	1.670256
H	-5.45361	0.463967	0.229284
H	-4.08719	0.780692	1.296367
H	-4.25958	2.554633	-0.38846
H	-4.23946	1.400705	-1.70871
H	0.434216	2.471049	1.166426
H	-0.25015	0.854196	1.24245
H	0.132985	0.671187	-1.29363
H	0.941412	2.227661	-1.22201
H	2.020281	-1.27127	-1.25387
H	-3.85767	-0.89824	-2.16726
H	-3.89572	-2.51838	-1.46474
H	-5.3328	-1.48834	-1.41561
H	-1.24726	-2.08296	-1.61131
H	-0.51607	-3.67605	-1.44676
H	0.469953	-2.29566	-1.9584
H	4.656521	0.020889	1.346478
H	2.760813	3.241907	-0.01719
H	3.855576	2.796229	1.303467
H	4.436186	2.768022	-0.36112
H	-1.50985	3.909339	1.679123
H	-3.137	3.371869	1.23495
H	-2.14892	2.397499	2.342973
H	5.639773	-1.78066	-0.63197

C	-3.27235	0.426353	-0.88896
C	-4.07692	-0.72685	-0.34682
O	-3.14778	-1.66059	0.129365
H	1.975679	0.784476	1.150659
C	-3.93176	1.547604	-1.62645
C	1.086496	3.619212	-0.76031
O	-5.03014	-0.34391	0.661528
O	-4.3242	0.303728	1.759125
H	2.313319	-1.15651	-1.82279
H	0.866215	-3.37245	-1.68944
H	1.129149	-4.39277	-0.27687
H	3.243206	-3.59909	-1.27524
H	3.085537	-3.14066	0.408233
H	-1.01501	-2.18422	-1.45993
H	4.854139	0.966455	-1.31399
H	3.148577	0.936551	-1.75966
H	3.70789	2.880513	-0.30872
H	4.301081	1.818886	0.953146
H	-1.0996	2.658432	0.73885
H	-0.29835	1.20273	1.321889
H	0.023942	0.52647	-1.14919
H	-1.04318	1.860222	-1.58622
H	-1.61048	-0.85901	1.264415
H	4.305789	-0.42065	1.68376
H	4.679798	-2.00334	0.993489
H	5.657329	-0.59224	0.572808
H	1.579776	-1.6535	1.664063
H	0.913775	-3.23128	2.073037
H	-0.10143	-1.78729	2.197685
H	-4.75671	-1.20238	-1.06718
H	-3.20981	2.303947	-1.94365
H	-4.45454	1.178154	-2.51736
H	-4.68752	2.040987	-1.00307
H	0.301909	4.365436	-0.59254
H	2.049248	4.130791	-0.73451
H	0.943778	3.19783	-1.76086
H	-4.54007	1.237643	1.599478

1g (4.58%)

C	3.095869	-1.41592	-0.89531
C	3.813341	-0.76188	0.034098
C	1.098847	-2.98504	-1.18533
C	2.601568	-2.83533	-0.86438
C	-0.91045	-1.6414	-0.65002
C	0.142188	-2.3443	-0.20013
C	4.282065	0.657384	-0.21871
C	3.706774	1.706988	0.75608
C	2.198048	1.681958	0.751185
C	1.359199	2.321653	-0.2758
C	0.006141	1.717182	-0.62816
C	-0.86581	1.296681	0.568571
C	-2.04999	0.491383	0.127035
C	-2.0113	-1.01992	0.159507
C	4.233188	-1.34695	1.360282
C	0.433069	-2.61043	1.254864
O	1.481087	2.902529	1.051216
C	-3.23588	0.90677	-0.33004
C	-4.09545	-0.29083	-0.63303
O	-3.28895	-1.42358	-0.39873
H	1.773701	0.806927	1.244131
C	-3.75863	2.29599	-0.50115
C	1.962147	3.142533	-1.39463
O	-5.31319	-0.35688	0.116539
O	-5.00197	-0.30573	1.53678
H	2.856382	-0.87403	-1.8121
H	0.876306	-4.06213	-1.21452
H	0.89807	-2.5988	-2.19117
H	2.82155	-3.31137	0.094439
H	3.150004	-3.41475	-1.62077
H	-1.02393	-1.49452	-1.72355
H	4.019326	0.946466	-1.24191
H	5.378286	0.689757	-0.14891
H	4.067679	2.700584	0.477995
H	4.064234	1.521734	1.775867
H	0.191848	0.847742	-1.26981
H	-0.56013	2.435193	-1.23396

H	-0.27839	0.697845	1.271496
H	-1.18511	2.189708	1.112423
H	-1.96794	-1.36274	1.201279
H	5.319834	-1.26308	1.48808
H	3.963522	-2.3981	1.469739
H	3.779078	-0.79836	2.195101
H	1.319108	-2.05059	1.576121
H	-0.39002	-2.33133	1.914433
H	0.653971	-3.6727	1.415275
H	-4.50567	-0.31749	-1.65306
H	-4.6642	2.441631	0.09864
H	-4.03064	2.491114	-1.54614
H	-3.02013	3.041972	-0.1976
H	2.035995	2.539648	-2.30662
H	1.316476	4.00047	-1.61169
H	2.954808	3.518664	-1.14451
H	-4.85944	-1.2494	1.725209

Table S2. Conformers and Boltzmann populations of compound **2**.

2a (35.30%)

C	2.357358	-1.89476	-1.13654
C	3.447088	-1.22765	-0.72097
C	0.359502	-3.32451	-0.47438
C	1.888679	-3.25516	-0.69206
C	-1.20754	-1.49027	0.064586
C	-0.18149	-2.26248	0.458799
C	3.779423	0.1424	-1.27941
C	3.948621	1.242997	-0.20959
C	2.690094	1.428015	0.598972
C	1.675249	2.476141	0.375582
C	0.247478	2.159066	0.778658
C	-0.55627	1.561798	-0.39548
C	-1.84463	0.927849	0.023753
C	-1.84338	-0.36129	0.820773
C	4.431764	-1.76395	0.290949
C	0.505186	-2.16571	1.797248
O	2.603211	2.566843	1.48742
C	-3.10291	1.300563	-0.24878

2b (49.49%)

C	2.423086	-1.81003	-1.10284
C	3.475003	-1.07619	-0.7029
C	0.510196	-3.34085	-0.42216
C	2.032892	-3.18838	-0.63898
C	-1.14752	-1.58845	0.118168
C	-0.08944	-2.3147	0.51496
C	3.730022	0.297936	-1.29115
C	3.84158	1.42972	-0.24712
C	2.580644	1.559106	0.568406
C	1.496204	2.528727	0.319154
C	0.096971	2.138131	0.755163
C	-0.69763	1.466899	-0.38522
C	-1.94085	0.785459	0.09273
C	-1.85397	-0.50698	0.878406
C	4.490754	-1.53789	0.314729
C	0.580068	-2.19681	1.860082
O	2.427392	2.721812	1.415867
C	-3.22244	1.1204	-0.09392

2c (1.79%)

C	2.394449	2.353902	0.551937
C	3.037064	1.276027	1.03318
C	0.148245	3.421669	-0.03225
C	1.152033	3.019437	1.077655
C	-1.38046	1.51241	-0.3955
C	-0.35105	2.244922	-0.85014
C	4.284845	0.767098	0.337586
C	4.227029	-0.7181	-0.08727
C	2.955823	-1.04116	-0.84398
C	2.01185	-2.13053	-0.51182
C	0.549861	-1.94508	-0.87593
C	-0.25463	-1.34793	0.297842
C	-1.65065	-0.9485	-0.06212
C	-1.90562	0.233401	-0.97749
C	2.628723	0.523095	2.273966
C	0.400858	1.970282	-2.12754
O	2.911581	-2.24797	-1.64057
C	-2.81504	-1.45692	0.365435

C	-4.04056	0.263285	0.312918
O	-3.25156	-0.61219	1.085697
H	2.323131	0.508152	1.058307
C	-3.61647	2.479019	-1.01271
C	1.838357	3.542767	-0.68416
O	-4.60263	-0.41133	-0.81824
O	-5.61241	-1.33558	-0.32434
H	1.722932	-1.40964	-1.8799
H	-0.15083	-3.24793	-1.44079
H	0.121577	-4.31712	-0.066
H	2.147665	-4.00677	-1.45136
H	2.400011	-3.56143	0.224253
H	-1.63206	-1.65074	-0.92604
H	3.002248	0.445967	-1.99026
H	4.720375	0.078716	-1.84385
H	4.767645	0.983617	0.471878
H	4.235825	2.17986	-0.69324
H	0.279289	1.452155	1.613229
H	-0.24888	3.066043	1.145239
H	0.059924	0.790184	-0.87686
H	-0.74638	2.33177	-1.14805
H	-1.36829	-0.22445	1.796222
H	4.375399	-1.2115	1.237532
H	5.458223	-1.64523	-0.07764
H	4.278388	-2.82039	0.514061
H	-0.02216	-1.52659	2.506811
H	0.599287	-3.16209	2.245897
H	1.52214	-1.77352	1.680499
H	-4.86903	0.629477	0.928567
H	-2.80338	3.125241	-1.35153
H	-4.19039	2.156498	-1.8883
H	-4.29074	3.080377	-0.39073
H	2.886601	3.807912	-0.82594
H	1.432355	3.205967	-1.64375
H	1.297	4.447742	-0.38687
H	-5.05414	-2.06293	0.005235

C	-4.10537	0.09211	0.561392
O	-3.24092	-0.86638	1.130593
H	2.280065	0.63428	1.064768
C	-3.82071	2.274568	-0.82989
C	1.578016	3.562703	-0.78147
O	-5.05415	-0.53059	-0.30652
O	-4.35306	-1.12766	-1.43139
H	1.761273	-1.37353	-1.85252
H	-0.00223	-3.28854	-1.38924
H	0.325286	-4.34601	-0.01721
H	2.559993	-3.45417	0.281042
H	2.334566	-3.93365	-1.38879
H	-1.54104	-1.75234	-0.88474
H	2.935207	0.542815	-2.0051
H	4.671227	0.273316	-1.85829
H	4.679786	1.235187	0.432684
H	4.068791	2.370842	-0.75384
H	0.184372	1.455325	1.605944
H	-0.44391	3.024179	1.110136
H	-0.94768	2.206454	-1.15085
H	-0.05277	0.716924	-0.86223
H	-1.38768	-0.34514	1.855548
H	4.419022	-0.95861	1.243946
H	5.507919	-1.38694	-0.06783
H	4.385069	-2.59234	0.572306
H	0.015359	-1.59095	2.569992
H	0.721041	-3.19118	2.300649
H	1.576854	-1.75255	1.754666
H	-4.78777	0.500173	1.322265
H	-3.05341	2.933872	-1.24224
H	-4.45062	1.917905	-1.65249
H	-4.46269	2.87075	-0.16964
H	2.60494	3.890907	-0.94564
H	1.186127	3.163334	-1.72273
H	0.980494	4.440677	-0.51203
H	-4.04673	-1.96136	-1.03233

C	-3.93649	-0.62557	-0.20224
O	-3.34909	0.25717	-1.13089
H	2.526408	-0.18613	-1.3684
C	-3.08888	-2.60188	1.287518
C	2.29049	-3.11327	0.604319
O	-4.49483	0.089451	0.905922
O	-5.66912	0.80229	0.425501
H	2.823277	2.830795	-0.33098
H	0.624397	4.155937	-0.69404
H	-0.6993	3.925059	0.445627
H	1.428902	3.93642	1.61893
H	0.635967	2.376313	1.796364
H	-1.8641	1.803553	0.537125
H	4.473219	1.384936	-0.54766
H	5.152483	0.892708	1.00017
H	4.331885	-1.36641	0.787344
H	5.085436	-0.93119	-0.735
H	0.116141	-2.90873	-1.17075
H	0.493073	-1.28651	-1.74785
H	0.272194	-0.45407	0.655714
H	-0.27854	-2.05719	1.129247
H	-1.49423	0.05726	-1.9753
H	2.442101	-0.53365	2.05604
H	3.438468	0.550135	3.01494
H	1.732854	0.931396	2.743777
H	-0.01945	1.152638	-2.71509
H	0.407597	2.869395	-2.7555
H	1.447894	1.729599	-1.91437
H	-4.7513	-1.16671	-0.69471
H	-3.71563	-3.35533	0.794908
H	-2.16617	-3.08931	1.610798
H	-3.63235	-2.26463	2.176883
H	1.809193	-4.07122	0.378415
H	3.36081	-3.29197	0.715736
H	1.895488	-2.7571	1.561087
H	-5.25573	1.556589	-0.03215

2d (7.25%)

2e (6.18%)

C	-2.74397	-1.58878	1.064329	C	-2.81648	-1.44652	1.047554
C	-3.67526	-0.96095	0.32592	C	-3.68829	-0.76605	0.28383
C	-0.63485	-3.02537	0.8832	C	-0.80138	-3.01763	0.938912
C	-2.17805	-2.96886	0.874524	C	-2.33668	-2.86201	0.883497
C	1.128407	-1.51388	0.024866	C	1.060858	-1.6305	0.078447
C	0.059428	-2.28316	-0.24069	C	-0.02963	-2.36755	-0.19079
C	-4.17836	0.410726	0.731058	C	-4.11601	0.635354	0.673795
C	-3.87629	1.53429	-0.28381	C	-3.73222	1.733978	-0.34058
C	-2.39595	1.629718	-0.55949	C	-2.2472	1.72731	-0.60803
C	-1.42753	2.290964	0.330226	C	-1.23864	2.317112	0.287666
C	0.004254	1.775866	0.398353	C	0.155469	1.708627	0.36062
C	0.670636	1.490224	-0.95998	C	0.789977	1.33956	-0.99226
C	1.951891	0.732052	-0.78667	C	2.037	0.528076	-0.81147
C	1.986743	-0.76954	-0.95851	C	1.993891	-0.98051	-0.90114
C	-4.31521	-1.53442	-0.91458	C	-4.33096	-1.305	-0.97044
C	-0.50177	-2.5215	-1.61945	C	-0.54534	-2.65661	-1.57753
O	-1.8337	2.913789	-0.91941	O	-1.59528	2.969727	-0.96209
C	3.161787	1.186919	-0.4348	C	3.279525	0.93411	-0.52996
C	4.091081	0.010245	-0.29728	C	4.178075	-0.26641	-0.41129
O	3.388688	-1.11262	-0.78485	O	3.362029	-1.39553	-0.64005
H	-2.01078	0.811533	-1.16855	H	-1.9183	0.886238	-1.2184
C	3.608816	2.58578	-0.1526	C	3.807319	2.317219	-0.32804
C	-1.86697	3.016787	1.582769	C	-1.63368	3.068928	1.539861
O	4.359864	-0.12108	1.10317	O	4.87521	-0.38494	0.830897
O	5.358806	-1.16776	1.266097	O	3.914554	-0.38432	1.922246
H	-2.36002	-1.05858	1.937805	H	-2.42162	-0.93556	1.927618
H	-0.34622	-4.08485	0.814779	H	-0.58014	-4.09511	0.917782
H	-0.25946	-2.66141	1.846357	H	-0.42989	-2.64115	1.898921
H	-2.55704	-3.43013	-0.04082	H	-2.71873	-3.30762	-0.03838
H	-2.5264	-3.60693	1.69921	H	-2.74938	-3.4672	1.70331
H	1.446541	-1.39435	1.059866	H	1.331049	-1.46347	1.120226
H	-3.74114	0.683731	1.697336	H	-3.6789	0.886995	1.645873
H	-5.26676	0.363131	0.87497	H	-5.20751	0.652386	0.800578
H	-4.40625	1.355058	-1.22671	H	-4.26724	1.589063	-1.28658
H	-4.2485	2.486044	0.104028	H	-4.0417	2.709507	0.043389
H	-0.00496	0.860394	1.001386	H	0.090282	0.814617	0.991506
H	0.622028	2.499425	0.943529	H	0.824768	2.404914	0.880104
H	-0.00122	0.906998	-1.59653	H	0.082595	0.763593	-1.59681

H 0.847685 2.436231 -1.47951	H 1.002707 2.255767 -1.5503
H 1.724555 -1.03771 -1.98726	H 1.755162 -1.28704 -1.92609
H -4.08756 -0.92074 -1.79523	H -4.04846 -0.70663 -1.84566
H -5.40794 -1.53903 -0.81355	H -5.42432 -1.24793 -0.89523
H -3.9949 -2.55445 -1.13047	H -4.06328 -2.34186 -1.17796
H 0.153173 -2.16719 -2.41682	H 0.161042 -2.38423 -2.36308
H -0.68455 -3.5909 -1.77982	H -0.78023 -3.72235 -1.68505
H -1.46717 -2.01458 -1.73211	H -1.47563 -2.10663 -1.76204
H 5.053047 0.078054 -0.81646	H 5.034178 -0.26026 -1.103
H 2.823758 3.308678 -0.38857	H 3.051109 3.071397 -0.56014
H 3.880232 2.7062 0.902252	H 4.127892 2.460415 0.710401
H 4.497366 2.839468 -0.74314	H 4.683422 2.50186 -0.96162
H -2.9118 3.326099 1.537658	H -2.6541 3.450586 1.489221
H -1.72978 2.373074 2.458758	H -1.54717 2.415232 2.414919
H -1.25134 3.911616 1.725309	H -0.95797 3.918176 1.688732
H 4.813126 -1.96622 1.147954	H 3.654681 -1.32217 1.933855

Table S3. Conformers and Boltzmann populations of compound **3**.

**3a** (41.43%)	**3b** (2.34%)	**3c** (2.23%)
C 2.86077 -1.61058 -1.01197	C 2.312003 -1.37428 -1.43759	C 2.945159 2.032861 0.33857
C 3.758796 -0.81251 -0.4107	C 3.49085 -0.89511 -1.00739	C 3.452108 0.850156 0.726137
C 1.020082 -3.34561 -0.74016	C 1.320309 -3.04554 0.202913	C 0.870508 3.508457 0.128089
C 2.534051 -3.04179 -0.67632	C 1.791029 -2.77891 -1.241	C 1.957818 2.911954 1.056208
C -0.89368 -1.80786 -0.44898	C -0.71012 -1.59871 -0.06523	C -1.0409 1.940083 0.07754
C 0.162323 -2.42656 0.103644	C 0.19728 -2.17439 0.738561	C 0.016777 2.461342 -0.56378
C 3.967857 0.613796 -0.88039	C 3.837895 0.57059 -1.16479	C 4.443912 0.124515 -0.16211
C 3.782422 1.679637 0.221269	C 3.869797 1.341136 0.176645	C 4.03336 -1.31621 -0.54259
C 2.394892 1.633792 0.8085	C 2.524856 1.300755 0.858154	C 2.615698 -1.37807 -1.07063
C 1.272539 2.508771 0.418083	C 1.470359 2.324172 0.720602	C 1.545989 -2.26251 -0.55938
C -0.13195 1.959438 0.578491	C 0.032253 1.860446 0.86153	C 0.10769 -1.79525 -0.68518
C -0.64521 1.297261 -0.71833	C -0.56288 1.455689 -0.50374	C -0.37522 -1.07395 0.591916
C -1.89119 0.497254 -0.50369	C -1.81324 0.639508 -0.40662	C -1.72687 -0.44996 0.440971
C -1.83993 -0.84568 0.201808	C -1.82255 -0.6814 0.343355	C -1.91863 0.814508 -0.37839
C 4.636208 -1.24436 0.740147	C 4.523654 -1.7353 -0.29628	C 3.131189 0.172379 2.034138
C 0.581853 -2.29176 1.545308	C 0.208753 -2.05055 2.242095	C 0.462535 2.064849 -1.94825
O 1.977113 2.71796 1.670405	O 2.259613 2.229834 1.935749	O 2.206687 -2.54722 -1.81732

C	-3.15382	0.783888	-0.84301
C	-4.05717	-0.31384	-0.3425
O	-3.19233	-1.35243	0.090849
H	2.113888	0.654017	1.199279
O	-4.84846	0.206569	0.711064
C	-3.71507	1.98735	-1.52848
C	1.433875	3.616223	-0.59961
C	-5.81649	-0.7165	1.203894
H	2.288705	-1.18991	-1.84034
H	0.681973	-3.29751	-1.78131
H	0.870248	-4.38209	-0.40517
H	3.035929	-3.71609	-1.38509
H	2.915916	-3.30506	0.313624
H	-1.10633	-1.98198	-1.50368
H	3.283976	0.829462	-1.7094
H	4.988997	0.71443	-1.27448
H	3.991981	2.669705	-0.19104
H	4.509451	1.520298	1.02666
H	-0.81563	2.763834	0.876478
H	-0.12295	1.230036	1.394288
H	0.139728	0.632886	-1.10283
H	-0.82038	2.062342	-1.48029
H	-1.59852	-0.71347	1.263422
H	4.3529	-0.73854	1.671917
H	5.68112	-0.97305	0.544391
H	4.599591	-2.3191	0.92175
H	1.526155	-1.73986	1.620952
H	-0.15744	-1.77703	2.160569
H	0.757275	-3.28205	1.983239
H	-4.7296	-0.74179	-1.10275
H	-2.93049	2.683178	-1.83605
H	-4.40439	2.519231	-0.86305
H	-4.28721	1.700253	-2.41973
H	0.699907	4.406927	-0.40839
H	1.269348	3.240954	-1.6151
H	2.428794	4.060303	-0.55158
H	-6.40725	-0.1851	1.952227
H	-6.47865	-1.05875	0.395384

C	-3.01986	0.863106	-0.94153
C	-3.93932	-0.27059	-0.56311
O	-3.10166	-1.26845	-0.00055
H	2.173003	0.291786	1.076522
O	-4.89647	0.219408	0.35983
C	-3.52402	2.020023	-1.74206
C	1.677479	3.591518	-0.07834
C	-5.89005	-0.74091	0.710234
H	1.637055	-0.67409	-1.92586
H	2.182954	-2.92557	0.869825
H	1.018225	-4.09898	0.302128
H	0.97454	-2.97143	-1.94453
H	2.568954	-3.51497	-1.47689
H	-0.64766	-1.75772	-1.13965
H	3.111569	1.050758	-1.83056
H	4.823686	0.668	-1.63892
H	4.620095	0.90352	0.846499
H	4.178046	2.374319	-0.00211
H	-0.57804	2.649481	1.317878
H	0.019764	1.007651	1.545759
H	-0.74393	2.345421	-1.11315
H	0.192095	0.857834	-1.03162
H	-1.80133	-0.50081	1.424153
H	4.558623	-1.5026	0.775718
H	4.331086	-2.80585	-0.39255
H	5.525251	-1.53157	-0.69409
H	0.310819	-3.04357	2.696928
H	1.077733	-1.4661	2.571922
H	-0.6885	-1.58542	2.652146
H	-4.47489	-0.73114	-1.408
H	-2.74627	2.767699	-1.91562
H	-3.90066	1.687437	-2.71776
H	-4.35928	2.505648	-1.22497
H	1.389871	3.444617	-1.12464
H	2.717184	3.919325	-0.04816
H	1.055929	4.394822	0.332635
H	-6.60114	-0.23522	1.365988
H	-6.41662	-1.10368	-0.1844

C	-2.90378	-0.84693	0.940486
C	-3.98381	0.084319	0.452732
O	-3.30498	1.166506	-0.16604
H	2.279955	-0.45169	-1.53927
O	-4.80946	-0.6249	-0.45331
C	-3.23925	-2.03504	1.782842
C	1.810013	-3.28889	0.520578
C	-5.93469	0.126353	-0.90242
H	3.293495	2.430575	-0.61612
H	1.35529	4.1438	-0.62393
H	0.232479	4.162173	0.732918
H	2.49035	3.754127	1.523131
H	1.463469	2.367617	1.866055
H	-1.28431	2.307532	1.074897
H	5.419941	0.074182	0.340419
H	4.591444	0.707602	-1.07818
H	4.156323	-1.98431	0.314622
H	4.714496	-1.68164	-1.32009
H	-0.54341	-2.65384	-0.8911
H	0.035682	-1.1245	-1.54675
H	0.350791	-0.29084	0.842833
H	-0.38952	-1.77892	1.427996
H	-1.76751	0.606042	-1.4446
H	4.049164	0.024522	2.618024
H	2.701412	-0.82167	1.872356
H	2.432961	0.742654	2.648343
H	1.469044	1.633471	-1.9199
H	-0.19933	1.342414	-2.42817
H	0.520084	2.952232	-2.59053
H	-4.6215	0.506538	1.245531
H	-2.34623	-2.59555	2.070395
H	-3.76287	-1.7318	2.698211
H	-3.90914	-2.71159	1.240131
H	1.118715	-4.13078	0.403466
H	1.659	-2.86813	1.519763
H	2.827589	-3.67693	0.457204
H	-6.52355	-0.53665	-1.5388
H	-6.55062	0.454693	-0.05265

H	-5.33455	-1.58546	1.665518

3d (44.43%)

C	2.907258	-1.51144	-0.98211
C	3.742266	-0.62979	-0.40725
C	1.193848	-3.35993	-0.63851
C	2.683727	-2.95036	-0.59866
C	-0.81739	-1.945	-0.38606
C	0.281107	-2.47107	0.179432
C	3.849366	0.790673	-0.92663
C	3.589655	1.879067	0.137407
C	2.208114	1.758587	0.728036
C	1.02988	2.545257	0.314723
C	-0.3339	1.906231	0.494072
C	-0.79388	1.159425	-0.77623
C	-1.97422	0.273109	-0.53174
C	-1.82091	-1.02396	0.239477
C	4.645128	-0.95632	0.758591
C	0.698537	-2.259	1.612429
O	1.718521	2.837418	1.558871
C	-3.24827	0.423224	-0.91275
C	-4.07416	-0.73104	-0.39067
O	-3.13658	-1.62429	0.196175
H	1.992834	0.774178	1.147793
O	-5.08599	-0.34469	0.526613
C	-3.89462	1.529956	-1.68164
C	1.114958	3.631076	-0.73468
C	-4.62053	0.337631	1.690071
H	2.309372	-1.16213	-1.8254
H	0.846669	-3.37243	-1.67767
H	1.117558	-4.39138	-0.26563
H	3.2258	-3.61026	-1.29109
H	3.090362	-3.15512	0.395173
H	-1.02555	-2.17272	-1.43139
H	3.152355	0.928082	-1.76135
H	4.860834	0.949444	-1.32641
H	3.731402	2.866186	-0.30937
H	4.324955	1.798046	0.946876

H	-5.44696	-1.59362	1.236524

3e (5.45%)

C	-3.0531	-1.52279	0.897978
C	-3.82817	-0.9009	-0.007
C	-0.97974	-2.99988	1.151386
C	-2.49086	-2.91617	0.846034
C	0.959888	-1.56466	0.592268
C	-0.06184	-2.31952	0.155885
C	-4.36909	0.48741	0.273203
C	-3.87496	1.580454	-0.6987
C	-2.36713	1.629419	-0.73672
C	-1.53114	2.273684	0.289743
C	-0.14174	1.727635	0.589249
C	0.702296	1.338508	-0.63716
C	1.956577	0.622438	-0.23564
C	2.019381	-0.89117	-0.23071
C	-4.24962	-1.4967	-1.32774
C	-0.35267	-2.60601	-1.2955
O	-1.71666	2.892293	-1.01304
C	3.134487	1.127526	0.148121
C	4.096168	-0.00555	0.397094
O	3.312226	-1.18951	0.346095
H	-1.91664	0.794257	-1.27266
O	5.09314	0.025359	-0.60867
C	3.565942	2.553929	0.262641
C	-2.14299	3.028919	1.449591
C	6.124227	-0.93966	-0.41639
H	-2.81925	-0.97536	1.812959
H	-0.71127	-4.06655	1.179092
H	-0.78596	-2.6043	2.155084
H	-3.00469	-3.52821	1.601272
H	-2.6984	-3.39231	-0.11556
H	1.076269	-1.40686	1.663934
H	-4.10061	0.777833	1.294462
H	-5.46677	0.459054	0.227528
H	-4.25253	1.394898	-1.71117
H	-4.27584	2.550145	-0.39209

H	-5.61941	1.003795	-1.4778

3f (2.34%)

C	2.992593	1.926968	0.279854
C	3.424661	0.728908	0.70888
C	1.011476	3.517095	0.011386
C	2.060831	2.890122	0.963285
C	-0.99008	2.064379	0.004742
C	0.100866	2.496921	-0.64686
C	4.369899	-0.08675	-0.15181
C	3.87525	-1.51413	-0.47925
C	2.455022	-1.51368	-1.00397
C	1.337767	-2.3149	-0.45855
C	-0.07091	-1.76799	-0.59912
C	-0.49818	-0.96062	0.645617
C	-1.80731	-0.25653	0.474022
C	-1.92382	0.968929	-0.4139
C	3.063428	0.118878	2.039538
C	0.531991	2.019535	-2.01049
O	1.975891	-2.6847	-1.70561
C	-2.99726	-0.52622	1.024236
C	-4.02723	0.458217	0.51725
O	-3.29432	1.397353	-0.25914
H	2.171887	-0.58793	-1.50723
O	-5.07695	-0.14115	-0.22572
C	-3.39568	-1.62439	1.956607
C	1.544945	-3.31329	0.659296
C	-4.66189	-0.87548	-1.37657
H	3.365396	2.269011	-0.68704
H	1.533422	4.097167	-0.76013
H	0.411086	4.226727	0.591103
H	2.643606	3.714892	1.400157
H	1.534316	2.406948	1.791509
H	-1.2207	2.486069	0.983491
H	5.342237	-0.17624	0.352383
H	4.550054	0.452544	-1.0886
H	3.961676	-2.15645	0.401759
H	4.532114	-1.94637	-1.24319

H -1.07272 2.672696 0.759473
H -0.27613 1.211568 1.337728
H 0.040012 0.542006 -1.13561
H -1.02458 1.879211 -1.56683
H -1.56618 -0.82524 1.288104
H 4.322093 -0.44188 1.672572
H 5.667685 -0.61494 0.554732
H 4.687801 -2.02403 0.976557
H 1.610265 -1.65237 1.662782
H -0.06632 -1.76588 2.214043
H 0.931579 -3.22137 2.084387
H -4.63451 -1.26119 -1.1688
H -3.16972 2.282228 -2.00227
H -4.65986 2.027877 -1.07431
H -4.40413 1.141565 -2.5722
H 0.335564 4.380605 -0.55799
H 0.965324 3.218169 -1.73775
H 2.081019 4.136465 -0.7089
H -5.50089 0.523701 2.30706
H -3.91058 -0.28065 2.250552
H -4.14593 1.294114 1.437953

H -0.26581 0.85354 1.239417
H 0.41553 2.471854 1.171701
H 0.12502 0.683917 -1.29767
H 0.934685 2.238432 -1.21314
H 1.99687 -1.2592 -1.2651
H -3.8663 -0.90391 -2.16774
H -5.34338 -1.49182 -1.41792
H -3.90698 -2.52324 -1.46379
H -0.53269 -3.67715 -1.44858
H 0.454872 -2.29844 -1.96157
H -1.26233 -2.08321 -1.61407
H 4.594726 0.014718 1.379153
H 3.859593 2.795656 1.292282
H 2.768052 3.240732 -0.03122
H 4.438205 2.742877 -0.37305
H -1.53131 3.907806 1.680963
H -2.16806 2.393929 2.342184
H -3.15705 3.367727 1.234272
H 5.729729 -1.95927 -0.48949
H 6.861778 -0.77529 -1.2041
H 6.605455 -0.8089 0.563753

H -0.7736 -2.59488 -0.76163
H -0.10841 -1.13464 -1.49071
H 0.277985 -0.21449 0.855981
H -0.54999 -1.62375 1.513689
H -1.7621 0.70181 -1.46589
H 3.970759 -0.06102 2.631031
H 2.576293 -0.85365 1.913241
H 2.399645 0.751319 2.630642
H 1.504918 1.519528 -1.95432
H -0.17439 1.328747 -2.47337
H 0.658133 2.876751 -2.68303
H -4.55976 0.990407 1.313361
H -2.5436 -2.2446 2.245411
H -3.84981 -1.21751 2.86861
H -4.14943 -2.27163 1.492613
H 0.805394 -4.11729 0.575401
H 1.422823 -2.84685 1.641986
H 2.537863 -3.76227 0.609278
H -5.57477 -1.22968 -1.85773
H -4.11106 -0.2346 -2.07383
H -4.03716 -1.73595 -1.10636

3g (1.79%)

C 2.359886 -1.32162 -1.39436
C 3.496888 -0.74407 -0.97242
C 1.480018 -3.01008 0.2926
C 1.940572 -2.75374 -1.15656
C -0.64592 -1.71924 -0.02504
C 0.29988 -2.19819 0.797692
C 3.738887 0.736827 -1.17755
C 3.706494 1.55259 0.136817
C 2.362682 1.439951 0.81231
C 1.239941 2.381156 0.634578
C -0.16264 1.820178 0.78141
C -0.71121 1.324397 -0.57292
C -1.89507 0.415315 -0.46201
C -1.81686 -0.86208 0.353187
C 4.581766 -1.48353 -0.22769

C	0.304123	-2.01428	2.29512
O	2.023219	2.383035	1.856799
C	-3.09336	0.499127	-1.05233
C	-3.93456	-0.69737	-0.66597
O	-3.06072	-1.54468	0.068757
H	2.08106	0.416432	1.062322
O	-5.10334	-0.36713	0.068562
C	-3.65763	1.560107	-1.94118
C	1.363194	3.632704	-0.20551
C	-4.87475	0.333878	1.28984
H	1.6404	-0.68881	-1.91035
H	2.328924	-2.81489	0.959226
H	1.246614	-4.07769	0.420598
H	1.145179	-3.0261	-1.85778
H	2.771241	-3.43843	-1.36592
H	-0.57494	-1.91772	-1.09242
H	2.985022	1.141122	-1.86281
H	4.718461	0.888743	-1.65016
H	4.480621	1.191696	0.824914
H	3.942374	2.598412	-0.07543
H	-0.83224	2.579695	1.203917
H	-0.12003	0.994219	1.49656
H	-0.95285	2.175595	-1.2154
H	0.093544	0.768886	-1.07299
H	-1.79209	-0.6293	1.424497
H	4.591104	-1.21446	0.836356
H	4.46781	-2.56774	-0.29007
H	5.569291	-1.22083	-0.62608
H	0.475426	-2.97926	2.78775
H	1.130498	-1.3583	2.59895
H	-0.6233	-1.59811	2.690457
H	-4.3303	-1.25231	-1.52374
H	-2.94791	2.373133	-2.11196
H	-3.9411	1.143248	-2.91578
H	-4.56922	1.984294	-1.50388
H	1.098473	3.430412	-1.24862
H	2.376132	4.035771	-0.17771
H	0.68097	4.402232	0.17258

H -5.85547 0.483896 1.743791

H -4.24864 -0.25474 1.96959

H -4.40232 1.308811 1.116369

Table S4. Conformers and Boltzmann populations of compound **4**

**4a** (40.13%)	**4b** (1.99%)	**4c** (32.40%)
C 2.373868 -1.89584 -1.13776	C 2.429723 2.34545 0.553226	C 2.444087 -1.80987 -1.09878
C 3.466708 -1.23022 -0.72811	C 3.065138 1.264767 1.037666	C 3.494779 -1.06746 -0.71174
C 0.376275 -3.32228 -0.46758	C 0.190295 3.423796 -0.03846	C 0.545115 -3.34903 -0.3969
C 1.904879 -3.25485 -0.68941	C 1.189551 3.018674 1.074419	C 2.065592 -3.18716 -0.62205
C -1.19469 -1.4912 0.073138	C -1.35876 1.530727 -0.40445	C -1.13239 -1.61134 0.130402
C -0.16346 -2.25776 0.463957	C -0.31724 2.248803 -0.8539	C -0.06052 -2.31577 0.528914
C 3.797041 0.140316 -1.28634	C 4.310236 0.745876 0.344689	C 3.735373 0.306453 -1.30633
C 3.966983 1.23987 -0.21551	C 4.241944 -0.7395 -0.07801	C 3.84278 1.442151 -0.26595
C 2.709785 1.422431 0.59584	C 2.970514 -1.05364 -0.83809	C 2.584328 1.563918 0.554695
C 1.692168 2.468006 0.373991	C 2.014694 -2.13235 -0.5056	C 1.491935 2.525194 0.307899
C 0.265681 2.149007 0.77922	C 0.555758 -1.93476 -0.8746	C 0.096883 2.125392 0.74885
C -0.54166 1.551795 -0.39232	C -0.24895 -1.32693 0.29348	C -0.69579 1.444756 -0.3874
C -1.83286 0.925344 0.031467	C -1.64439 -0.92873 -0.0727	C -1.93793 0.76382 0.095455
C -1.83859 -0.36452 0.827795	C -1.90002 0.256415 -0.98435	C -1.852 -0.5304 0.880447
C 4.455686 -1.76787 0.27897	C 2.649675 0.51617 2.278722	C 4.521573 -1.51816 0.299814
C 0.52828 -2.15539 1.799725	C 0.439885 1.959654 -2.1255	C 0.616639 -2.17087 1.867807
O 2.623093 2.561487 1.484051	O 2.918461 -2.26278 -1.63061	O 2.426044 2.727062 1.400632
C -3.08929 1.296718 -0.24405	C -2.80969 -1.43446 0.350747	C -3.21965 1.113776 -0.06257
C -4.04143 0.271719 0.316152	C -3.93979 -0.61239 -0.21332	C -4.11527 0.085261 0.591436
O -3.24296 -0.6218 1.0769	O -3.33928 0.297002 -1.12314	O -3.2324 -0.89386 1.127728
H 2.345693 0.501987 1.056148	H 2.55116 -0.19655 -1.36714	H 2.292388 0.637938 1.053876
O -4.69144 -0.37221 -0.76452	O -4.59063 0.047339 0.857236	O -5.07419 -0.49192 -0.27549
C -3.59722 2.464928 -1.02602	C -3.08437 -2.57129 1.281536	C -3.81071 2.299116 -0.75539
C 1.851981 3.534243 -0.68672	C 2.280826 -3.11398 0.614629	C 1.563002 3.558131 -0.79455
C -5.67982 -1.31319 -0.353	C -5.7385 0.78937 0.452702	C -4.54228 -1.17257 -1.41077
H 1.735633 -1.40949 -1.87701	H 2.862486 2.817578 -0.33036	H 1.77282 -1.3812 -1.8446
H -0.13636 -3.24792 -1.43302	H 0.672804 4.153157 -0.70147	H 0.028108 -3.31411 -1.36241
H 0.139076 -4.31427 -0.05688	H -0.65426 3.934764 0.436805	H 0.370422 -4.35064 0.021319
H 2.162004 -4.00842 -1.4476	H 0.668196 2.379433 1.79282	H 2.370358 -3.93687 -1.36628
H 2.418214 -3.55922 0.226493	H 1.470418 3.935101 1.614878	H 2.59838 -3.44047 0.29825
H -1.625 -1.65804 -0.91393	H -1.8479 1.833617 0.521482	H -1.53551 -1.80538 -0.86283
H 3.018018 0.443926 -1.99513	H 4.504297 1.360917 -0.54132	H 2.934553 0.542543 -2.01661

H 4.736996 0.078193 -1.85272
H 4.787776 0.980601 0.463959
H 4.252028 2.177747 -0.69855
H -0.23035 3.055523 1.147792
H 0.29931 1.441387 1.613223
H 0.072202 0.777971 -0.87321
H -0.72947 2.32174 -1.14586
H -1.36781 -0.2298 1.806554
H 5.480738 -1.6479 -0.09323
H 4.303542 -2.82483 0.500569
H 4.402462 -1.21736 1.226897
H 0.003606 -1.51119 2.506718
H 0.621854 -3.1495 2.253978
H 1.54572 -1.76544 1.678865
H -4.81255 0.679774 0.989515
H -4.17454 2.124581 -1.8929
H -4.26894 3.08092 -0.41494
H -2.78366 3.102774 -1.38001
H 1.307541 4.437658 -0.3902
H 2.899363 3.802719 -0.82899
H 1.44653 3.195111 -1.6457
H -6.16414 -1.67927 -1.26013
H -5.22547 -2.15216 0.185541
H -6.43157 -0.83515 0.291731

4d (1.53%)

C 1.735701 -1.63265 -1.43443
C 3.029623 -1.32406 -1.24769
C 0.824213 -3.04059 0.478645
C 1.05627 -2.92414 -1.0416
C -0.97554 -1.29555 0.461639
C -0.01915 -1.95803 1.130727
C 3.556534 0.055111 -1.58569
C 3.95429 0.88443 -0.34139
C 2.768866 1.102109 0.565131
C 1.876727 2.277242 0.532434
C 0.433996 2.060422 0.951262
C -0.44408 1.680446 -0.25832

H 5.17803 0.866003 1.0082
H 5.100632 -0.96045 -0.72284
H 4.338999 -1.38717 0.797993
H 0.114837 -2.89573 -1.16801
H 0.506936 -1.2785 -1.74883
H -0.27594 -2.03162 1.129019
H 0.281225 -0.43329 0.646925
H -1.49451 0.079811 -1.98554
H 1.760404 0.93611 2.750751
H 2.447661 -0.53754 2.059866
H 3.461479 0.531264 3.017843
H 0.012235 1.14445 -2.71121
H 0.461727 2.855602 -2.75795
H 1.482597 1.706254 -1.90585
H -4.69359 -1.18983 -0.77255
H -2.16356 -3.04972 1.623899
H -3.64201 -2.22281 2.157956
H -3.701 -3.33573 0.792055
H 1.791208 -4.06809 0.390197
H 3.349088 -3.30232 0.730245
H 1.885559 -2.751 1.568665
H -6.19513 1.185147 1.361689
H -5.46002 1.616758 -0.20927
H -6.46166 0.141769 -0.0639

4e (7.39%)

C -2.76317 -1.58424 1.070385
C -3.69788 -0.95192 0.340205
C -0.65923 -3.02759 0.876006
C -2.20215 -2.96517 0.872849
C 1.117089 -1.53007 0.017826
C 0.036097 -2.28271 -0.24589
C -4.19342 0.420665 0.751246
C -3.89293 1.544833 -0.26345
C -2.41432 1.631975 -0.55181
C -1.43364 2.279045 0.334833
C -0.00496 1.754839 0.388174
C 0.648834 1.460724 -0.97392

H 4.673733 0.288151 -1.87842
H 4.685408 1.255748 0.410658
H 4.060658 2.383621 -0.77615
H -0.44987 3.008375 1.102509
H 0.190817 1.445309 1.601031
H -0.04796 0.694075 -0.8592
H -0.94705 2.180332 -1.15694
H -1.38301 -0.35831 1.855838
H 5.534777 -1.35656 -0.08884
H 4.428388 -2.57364 0.557924
H 4.449192 -0.93973 1.229545
H 0.050267 -1.55914 2.571326
H 0.768652 -3.15712 2.323063
H 1.609033 -1.71967 1.750554
H -4.73211 0.501363 1.398071
H -4.51142 1.984308 -1.53724
H -4.38313 2.915171 -0.05057
H -3.04359 2.930362 -1.21058
H 0.959562 4.431958 -0.52476
H 2.586972 3.893863 -0.96194
H 1.171655 3.154444 -1.73421
H -5.4021 -1.51588 -1.98815
H -3.9294 -0.50751 -2.03254
H -3.9399 -2.03372 -1.10445

4f (1.41%)

C -2.76317 -1.58424 1.070385
C -3.69788 -0.95192 0.340205
C -0.65923 -3.02759 0.876006
C -2.20215 -2.96517 0.872849
C 1.117089 -1.53007 0.017826
C 0.036097 -2.28271 -0.24589
C -4.19342 0.420665 0.751246
C -3.89293 1.544833 -0.26345
C -2.41432 1.631975 -0.55181
C -1.43364 2.279045 0.334833
C -0.00496 1.754839 0.388174
C 0.648834 1.460724 -0.97392

C	-1.7594	1.054331	0.088584
C	-1.82212	-0.16912	0.980736
C	4.036427	-2.27704	-0.65036
C	0.322101	-1.73248	2.582872
O	2.850738	2.114295	1.596791
C	-2.98106	1.343027	-0.3776
C	-3.95685	0.306515	0.115293
O	-3.22951	-0.51457	1.016028
H	2.312313	0.177282	0.919463
O	-4.43774	-0.41882	-1.0014
C	-3.42761	2.424068	-1.30863
C	2.126465	3.452014	-0.38672
C	-5.43883	-1.37683	-0.66705
H	1.095038	-0.86294	-1.86029
H	1.80304	-3.04463	0.973368
H	0.375735	-4.0188	0.70822
H	1.658161	-3.78671	-1.35242
H	0.10475	-3.01538	-1.57517
H	-1.16467	-1.53954	-0.58155
H	4.439624	-0.03586	-2.23223
H	2.799066	0.609105	-2.15199
H	4.375537	1.841566	-0.65906
H	4.741691	0.368101	0.22121
H	0.03992	2.961726	1.437137
H	0.41907	1.260038	1.695821
H	-0.60392	2.553357	-0.89716
H	0.117376	0.951718	-0.85913
H	-1.54063	0.083423	2.007395
H	4.288741	-1.99731	0.380583
H	3.676144	-3.30775	-0.63068
H	4.973474	-2.25395	-1.21989
H	1.322572	-1.29046	2.678271
H	-0.38509	-1.08852	3.10715
H	0.35557	-2.69363	3.11072
H	-4.82385	0.713425	0.66133
H	-3.82621	1.992908	-2.23376
H	-4.23397	3.016608	-0.85856
H	-2.61206	3.103628	-1.56729

C	1.937808	0.712191	-0.8084
C	1.982931	-0.79115	-0.9646
C	-4.34795	-1.52047	-0.89732
C	-0.53648	-2.50274	-1.62335
O	-1.84666	2.915137	-0.90645
C	3.145775	1.173557	-0.46469
C	4.092055	0.012036	-0.31456
O	3.378038	-1.12885	-0.77161
H	-2.04016	0.816353	-1.17092
O	4.460291	-0.09563	1.048974
C	3.582317	2.575318	-0.18359
C	-1.85884	2.998437	1.596204
C	5.429534	-1.11094	1.296532
H	-2.37114	-1.057	1.942097
H	-0.27914	-2.67031	1.839909
H	-0.37593	-4.08827	0.802206
H	-2.5507	-3.60575	1.695675
H	-2.58571	-3.42077	-0.0435
H	1.445615	-1.42414	1.05102
H	-3.74888	0.689973	1.71521
H	-5.28114	0.377199	0.901973
H	-4.43227	1.371119	-1.2021
H	-4.25581	2.498006	0.129825
H	0.622333	2.47585	0.926038
H	-0.01352	0.840837	0.993528
H	0.812471	2.403238	-1.50439
H	-0.02651	0.866781	-1.59728
H	1.727649	-1.07105	-1.99294
H	-5.44003	-1.52234	-0.78888
H	-4.03169	-2.54073	-1.11793
H	-4.12449	-0.9052	-1.77795
H	-1.49877	-1.98743	-1.72525
H	0.116451	-2.14385	-2.42038
H	-0.72822	-3.56911	-1.79474
H	5.012682	0.087392	-0.91558
H	3.872198	2.686877	0.867525
H	4.458691	2.842438	-0.78727
H	2.787698	3.294316	-0.3994

C	1.937808	0.712191	-0.8084
C	1.982931	-0.79115	-0.9646
C	-4.34795	-1.52047	-0.89732
C	-0.53648	-2.50274	-1.62335
O	-1.84666	2.915137	-0.90645
C	3.145775	1.173557	-0.46469
C	4.092055	0.012036	-0.31456
O	3.378038	-1.12885	-0.77161
H	-2.04016	0.816353	-1.17092
O	4.460291	-0.09563	1.048974
C	3.582317	2.575318	-0.18359
C	-1.85884	2.998437	1.596204
C	5.429534	-1.11094	1.296532
H	-2.37114	-1.057	1.942097
H	-0.27914	-2.67031	1.839909
H	-0.37593	-4.08827	0.802206
H	-2.5507	-3.60575	1.695675
H	-2.58571	-3.42077	-0.0435
H	1.445615	-1.42414	1.05102
H	-3.74888	0.689973	1.71521
H	-5.28114	0.377199	0.901973
H	-4.43227	1.371119	-1.2021
H	-4.25581	2.498006	0.129825
H	0.622333	2.47585	0.926038
H	-0.01352	0.840837	0.993528
H	0.812471	2.403238	-1.50439
H	-0.02651	0.866781	-1.59728
H	1.727649	-1.07105	-1.99294
H	-5.44003	-1.52234	-0.78888
H	-4.03169	-2.54073	-1.11793
H	-4.12449	-0.9052	-1.77795
H	-1.49877	-1.98743	-1.72525
H	0.116451	-2.14385	-2.42038
H	-0.72822	-3.56911	-1.79474
H	5.012682	0.087392	-0.91558
H	3.872198	2.686877	0.867525
H	4.458691	2.842438	-0.78727
H	2.787698	3.294316	-0.3994

H 1.63843 3.30052 -1.35519
H 1.716432 4.365348 0.058548
H 3.192381 3.607912 -0.55694
H -5.78231 -1.81528 -1.60576
H -5.03231 -2.16276 -0.02111
H -6.28749 -0.89573 -0.15953

4g (1.91%)

C 1.777705 -1.58808 -1.39133
C 3.050498 -1.18991 -1.23093
C 0.987216 -3.00414 0.569107
C 1.192497 -2.91162 -0.95618
C -0.91729 -1.37444 0.523048
C 0.063982 -1.982 1.208103
C 3.478506 0.213654 -1.60511
C 3.83021 1.094562 -0.38274
C 2.642383 1.250629 0.53355
C 1.67289 2.362557 0.487196
C 0.253879 2.061914 0.933915
C -0.62101 1.605882 -0.25216
C -1.8873 0.911043 0.141781
C -1.85038 -0.31543 1.032297
C 4.126776 -2.05946 -0.62772
C 0.359266 -1.74653 2.668574
O 2.668633 2.289547 1.541077
C -3.14683 1.15611 -0.23865
C -4.06652 0.096688 0.327061
O -3.22254 -0.77987 1.063286
H 2.253623 0.305274 0.914345
O -4.81578 -0.6025 -0.65094
C -3.69615 2.241911 -1.10714
C 1.831074 3.529702 -0.46159
C -4.0428 -1.2952 -1.62981
H 1.082106 -0.87244 -1.82546
H 1.968391 -2.91523 1.05152
H 0.62344 -4.00915 0.830631
H 1.848744 -3.73875 -1.25239
H 0.243457 -3.0837 -1.47448

H -1.23549 3.887584 1.74104
H -2.90171 3.315848 1.561478
H -1.71971 2.347039 2.466203
H 5.690103 -1.04807 2.354672
H 5.024404 -2.10475 1.076017
H 6.33249 -0.94711 0.690651

4h (5.97%)

C -2.84695 -1.42961 1.048479
C -3.71621 -0.73314 0.296321
C -0.8544 -3.02901 0.917667
C -2.38717 -2.84993 0.869932
C 1.047407 -1.67908 0.084045
C -0.07346 -2.364 -0.19759
C -4.11918 0.673009 0.694913
C -3.72369 1.768401 -0.31857
C -2.241 1.736661 -0.59783
C -1.21615 2.302539 0.294917
C 0.169004 1.673114 0.353564
C 0.783853 1.283407 -1.00237
C 2.034832 0.477574 -0.82167
C 1.996524 -1.03391 -0.88489
C -4.37791 -1.25665 -0.95465
C -0.6147 -2.58231 -1.58768
O -1.57002 2.970586 -0.94688
C 3.284029 0.890397 -0.58182
C 4.197181 -0.30492 -0.43486
O 3.35529 -1.44262 -0.60218
H -1.93115 0.895043 -1.21716
O 4.90689 -0.35667 0.789288
C 3.814877 2.282334 -0.45602
C -1.59013 3.051339 1.555573
C 4.099646 -0.38141 1.964632
H -2.43714 -0.92902 1.927733
H -0.47436 -2.68233 1.885591
H -0.65026 -4.10911 0.870828
H -2.80629 -3.45782 1.684581
H -2.77842 -3.28022 -0.05534

H -1.23549 3.887584 1.74104
H -2.90171 3.315848 1.561478
H -1.71971 2.347039 2.466203
H 5.690103 -1.04807 2.354672
H 5.024404 -2.10475 1.076017
H 6.33249 -0.94711 0.690651

4i (7.27%)

C -1.51374 2.558539 0.725194
C -0.77532 2.106277 1.75153
C -2.99149 1.815105 -1.20216
C -2.89504 2.136286 0.299769
C -1.74706 -0.34055 -0.88768
C -2.31477 0.556276 -1.70747
C 0.626125 2.638717 1.970759
C 1.734363 1.610707 1.644305
C 1.642883 1.151279 0.212399
C 2.775026 0.802393 -0.66652
C 2.593852 -0.14937 -1.84486
C 1.162578 -0.60758 -2.13918
C 0.467858 -1.50295 -1.14893
C -1.04616 -1.61903 -1.26827
C -1.2145 1.025369 2.708934
C -2.34748 0.424455 -3.21098
O 2.079495 2.069036 -0.82213
C 0.883408 -2.24097 -0.10893
C -0.3182 -2.88788 0.537289
O -1.40031 -2.67321 -0.34773
H 0.684773 0.698014 -0.02135
O -0.51681 -2.27451 1.802929
C 2.220182 -2.44024 0.53711
C 4.210766 0.920366 -0.20286
C -1.59951 -2.83114 2.543235
H -1.06697 3.327202 0.09216
H -2.59355 2.668144 -1.77204
H -4.05296 1.754573 -1.48428
H -3.2582 1.287176 0.885855
H -3.5938 2.962462 0.494408

H -1.05912 -1.62086 -0.52649
H 4.359473 0.169053 -2.25926
H 2.67927 0.701944 -2.17446
H 4.181799 2.071209 -0.72472
H 4.656779 0.645551 0.181336
H -0.18865 2.945165 1.411044
H 0.300948 1.275543 1.691986
H -0.84785 2.455638 -0.90189
H -0.02694 0.90157 -0.85033
H -1.58577 -0.03162 2.056536
H 4.374655 -1.73877 0.392258
H 3.836589 -3.11104 -0.57861
H 5.051808 -1.98773 -1.21252
H 1.322741 -1.23339 2.786496
H -0.40358 -1.16078 3.182802
H 0.452434 -2.70739 3.189357
H -4.8419 0.501961 0.98932
H -4.18698 1.822471 -1.99311
H -4.45949 2.818361 -0.56989
H -2.91869 2.934138 -1.43914
H 1.342756 3.32308 -1.41973
H 1.365731 4.423575 -0.03154
H 2.881755 3.75309 -0.64998
H -4.75978 -1.77082 -2.30075
H -3.40709 -0.60991 -2.20512
H -3.41478 -2.06058 -1.16284
H 1.339551 -1.56852 1.127122
H -3.67166 0.913903 1.664997
H -5.2094 0.707647 0.828783
H -4.26841 1.636172 -1.26083
H -4.01288 2.748036 0.070838
H 0.854117 2.365395 0.858588
H 0.098687 0.784216 0.991652
H 0.984999 2.190667 -1.5789
H 0.06887 0.694135 -1.58489
H 1.764744 -1.34696 -1.91095
H -5.46958 -1.1837 -0.86918
H -4.12749 -2.29625 -1.16945
H -4.09444 -0.65843 -1.8297
H -1.52739 -1.99388 -1.73947
H 0.092124 -2.30287 -2.37047
H -0.88744 -3.63447 -1.73396
H 4.998532 -0.33111 -1.18408
H 4.210234 2.463516 0.550676
H 4.645662 2.447941 -1.15306
H 3.042398 3.028296 -0.65975
H -0.89875 3.887493 1.707001
H -2.60375 3.45158 1.513999
H -1.51086 2.389156 2.424972
H 4.791643 -0.36986 2.808197
H 3.440082 0.493547 2.023283
H 3.492247 -1.29149 2.003916
H -1.73421 -0.16052 0.181575
H 0.744113 2.951998 3.016921
H 0.785114 3.525178 1.347585
H 1.634979 0.728879 2.290181
H 2.709588 2.057953 1.853405
H 2.971823 0.37791 -2.73048
H 3.25166 -1.01293 -1.71267
H 0.535215 0.281831 -2.27866
H 1.15614 -1.11667 -3.11362
H -1.33967 -1.95799 -2.26737
H -0.67985 0.086715 2.51867
H -2.28287 0.81064 2.642654
H -0.99558 1.319755 3.742965
H -1.70145 1.18193 -3.67355
H -2.01773 -0.55314 -3.56711
H -3.36127 0.601411 -3.59046
H -0.22883 -3.97468 0.690063
H 2.256171 -1.91164 1.497198
H 2.382921 -3.50265 0.754592
H 3.049831 -2.09266 -0.07411
H 4.863206 1.129037 -1.05836
H 4.543993 -0.01818 0.253951
H 4.336358 1.725265 0.522094
H -1.61116 -2.328 3.511853
H -2.55312 -2.66366 2.030626
H -1.45637 -3.91037 2.696501
